# New Derivatives of 4-Piperidinylphenyl-Linked Thiazoles as VEGFR2 Inhibitors with Potential Cytotoxicity Against Renal Cancer

**DOI:** 10.3390/biom16030370

**Published:** 2026-02-28

**Authors:** Huda K. Mahmoud, Thoraya A. Farghaly, Hossa F. Alshareef, Amani M. R. Alsaedi, Afaf Y. Khormi, Hanan Gaber Abdulwahab, Alaa M. Abu Alnjaa, Shadia M. Hussein

**Affiliations:** 1 Department of Chemistry, Faculty of Science, University of Cairo, Giza 12613, Egypt; 2 Department of Chemistry, Faculty of Science, Umm Al-Qura University, Makkah 21955, Saudi Arabia; 3 Department of Chemistry, Collage of Science, Taif University, P.O. Box 11099, Taif 21944, Saudi Arabia; 4 Department of Chemistry, Faculty of Science, King Khalid University, P.O. Box 9004, Abha 61413, Saudi Arabia; 5 Department of Pharmaceutical Medicinal Chemistry and Drug Design, Faculty of Pharmacy (Girls), Al-Azhar University, Cairo 11754, Egypt; 6 Department of Chemistry, Jamoum University College, Umm Al-Qura University, Makkah 21955, Saudi Arabia; 7 Department of Chemical Pathology, Cairo University Hospital, University of Cairo, Giza 12613, Egypt

**Keywords:** thiazole, VEGFR2 inhibitors, cytotoxic, renal cancer, docking study

## Abstract

Herein, a novel series of 4-piperidinylphenyl-linked thiazoles was synthesized as VEGFR2 inhibitors with potential cytotoxic activity against renal cancer. Most of the target compounds inhibited VEGFR2 enzyme at sub-micromolar IC_50_ values. Compounds **7c** (IC_50_ = 0.073 ± 0.002 µM), **9b** (IC_50_ = 0.049 ± 0.002 µM), and **9c** (IC_50_ = 0.093 ± 0.003 µM) were the most potent, showing VEGFR2 inhibition superior to that of sunitinib (IC_50_ = 0.118 ± 0.003 µM). Furthermore, compounds **7c**, **9b**, and **9c** effectively inhibited the growth of A498 renal cancer cells, with compound **7c** being the most potent showing a one-digit IC_50_ value of 7.866 ± 0.27 µM. In addition, compound **7c** revealed a potentially improved safety profile against non-cancerous normal cells, relative to sunitinib. The treatment of A498 renal cancer cells with compound **7c** led to an apparent cell cycle arrest and a significant induction of apoptosis. A docking study was also conducted and revealed a proper orientation of compound **7c** into the active site of VEGFR2.

## 1. Introduction

Vascular endothelial growth factor-2 (VEGFR2), a transmembrane tyrosine kinase receptor, has a crucial role in facilitating angiogenesis [1,2]. Angiogenesis is a multifaceted process that entails the development of new vascular structures from established blood vessels. Angiogenesis is a crucial biological process that supplies nutrition and oxygen to growing cells, facilitating tumor growth, development, and invasion. VEGFR2 is evidently overexpressed and/or hyperactivated throughout the course of cancer progression. Therefore, targeting VEGFR2 signaling pathway is well-established as an important approach for targeted antiangiogenic therapy, and several VEGFR2 inhibitors have been clinically approved as anticancer agents [3,4,5,6,7,8].

**Figure 1:**
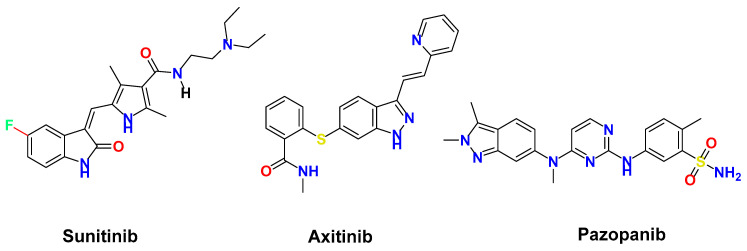
Clinically approved VEGFR2 inhibitors.

Moreover, kidney cancer ranks among the most prevalent malignancies in both men and women, exhibiting a steadily increasing global incidence along with significant morbidity and fatality rates worldwide [9]. Being a highly vascularized solid tumor, angiogenesis process is crucial in the progression and dissemination of renal cancer. Renal cell carcinoma has been molecularly linked to VEGF signaling pathway [2,5]. Accordingly, significant efforts have been undertaken to produce anti-angiogenic medications to treat renal cancer, specifically VEGFR2 inhibitors. As a result, small-molecule VEGFR2 inhibitors, including sunitinib, axitinib, and pazopanib, have been clinically approved for the treatment of advanced renal carcinoma [10,11] (Figure 1). Despite being efficacious in the treatment of renal cell carcinoma, the development of resistance to currently approved VEGFR2 inhibitors such as sunitinib remains a clinical challenge [12]. Therefore, there is an urgent need for the discovery and development of new treatments targeting VEGFR2.

**Figure 2:**
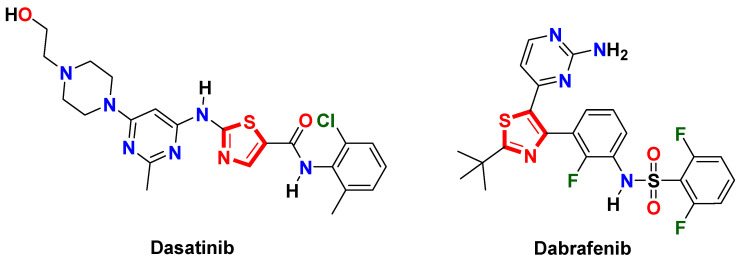
Clinically approved thiazole-containing tyrosine kinase inhibitors.

For decades, thiazole heterocycle has been an attractive core in medicinal chemistry for the design and discovery of numerous drug candidates, especially targeted anticancer drugs [13]. Several thiazole-based kinase inhibitors, such as dasatinib and dabrafenib, have reached clinical approval and resulted in a significant therapeutic response against different types of malignancies [14,15] (Figure 2).

**Figure 3:**
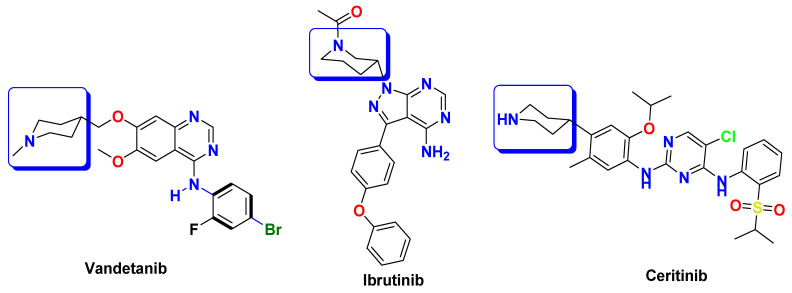
Clinically approved piperidine-containing tyrosine kinase inhibitors.

On the other hand, piperidine is a significant building block in medicinal chemistry and is extensively utilized for the synthesis of both natural and synthetic compounds. Piperidine moiety is found in various clinically used tyrosine kinase inhibitors, such as vandetanib, ibrutinib, and ceritinib [16,17,18] (Figure 3).

**Figure 4:**
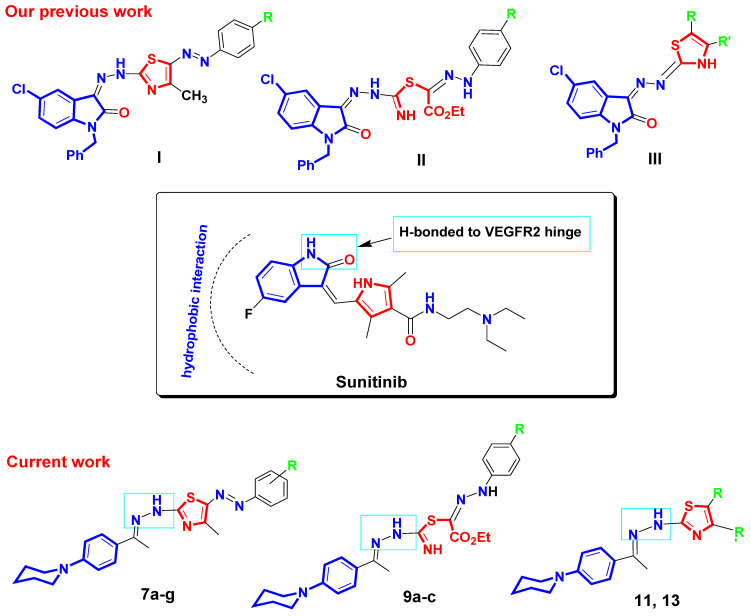
Design of target compounds as VEGFR2 inhibitors illustrating similarities between sunitinib, our previousy and currently reported compounds.

As mentioned before, sunitinib is an FDA-approved VEGFR2 inhibitor used for the treatment of renal cell carcinoma. Regarding VEGFR2–sunitinib interaction at molecular level, the NH group and ketone oxygen of the indolinone core of sunitinib form two hydrogen bonds with VEGFR2 hinge residues, Glu917 and Cys919. The indolinone core of sunitinib also makes hydrophobic contact with hydrophobic residues located in the active site of VEGFR2 [8]. It has been reported that the binding affinity towards the active site of VEGFR2 enzyme could be enhanced by the introduction of hydrophobic moieties that improve the interaction with hydrophobic pockets within the active site of VEGFR2 kinase [19,20]. In this context, we have previously reported a series of *N*-benzyl-indolinone-thiazole hybrids **I**, **II**, and **III** as sunitinib analogs with potential anti-VEGFR2 effects as well as anti-renal cancer activity [21]. Motivated by these findings and in a continuation of our efforts towards the discovery of VEGFR2 inhibitors [21,22]; herein, we synthesized a series of 4-piperidinylphenyl-linked thiazoles as VEGFR2 inhibitors with potential cytotoxic activity against renal carcinoma. As illustrated in Figure 4, the indolinone core in sunitinib and the *N*-benzylindolinone core in our previously reported compounds **I**, **II**, and **III** were replaced by the hydrophobic and simpler 4-piperidinylphenyl fragment in our currently reported compounds **7a–g**, **9a–c**, **11**, and **13**. The 4-piperidinylphenyl fragment in our target compounds is proposed to improve the binding affinity towards the binding pocket of VEGFR2 via hydrophobic interactions. In addition, the hydrazinyl linker in the target compounds is assumed to participate in H-bonding interactions with the VEGFR2 hinge residues. Moreover, the pyrrole ring in sunitinib is replaced by a thiazole ring in compounds **7a–g**, **11** and **13**. Compounds **9a–c** are designed as open thiazole analogs. Substituents of different sizes and electronic properties **R**, **R’** are also added to explore their impact on VEGFR2 inhibitory activity of target compounds.

In this work, all target compounds are evaluated in vitro for their activity against VEGFR2 kinase. The most active derivatives are further assessed for their cytotoxic effects on kidney cancer cells and normal non-cancerous cells. Furthermore, the impact of target compounds on cell cycle analysis and apoptosis is examined. A docking analysis is also conducted to predict the binding mode of target molecules within the active site of VEGFR2 enzyme.

## 2. Materials and Methods

### 2.1. Materials and Instruments

Each chemical and solvent were bought from commercial providers, such as Merck (Rahway, NJ, USA) and Aldrich (St. Louis, MO, USA), and were used without any additional purification. The melting points were determined with the help of a BUCHI 530 capillary apparatus, and the results have been provided without any corrections. For the purpose of obtaining infrared spectra, a Perkin Elmer FT-IR spectrometer (Spectrum 400, Shimadzu, Tokyo, Japan) equipped with an Attenuated Total Reflectance (ATR) accessory was utilized. The acquisition of these spectra encompassed a range of 4000–450 cm^−1^. A Bruker 400 MHz spectrometer (Varian, Inc., Karlsruhe, Germany) was utilized to obtain proton (^1^H) and carbon (^13^C) nuclear magnetic resonance (NMR) data. Tetramethylsilane (TMS) was utilized as the internal reference standard. According to the solubility of the sample, the samples were prepared by dissolving in either CDCl_3_ or DMSO-d_6_. Using a Costech ECS 4010 (CHN) analyzer (elementar Analysensysteme GmbH, Hanau, Germany), the elemental composition (representing carbon, hydrogen, and nitrogen) was determined. Mass spectra of the novel derivatives were recorded on Shimadzu GCMS-QP 1000 EX mass spectrometer (Tokyo, Japan) at 70 eV, and the heating rate was 40 °C/min. The renal cancer cells were obtained from American type culture collection. The hydrazonoyl chloride derivatives **6a–g** were prepared according to previously reported methods [23].

### 2.2. General Method for the Synthesis of Compounds ***7a–g***, ***9a–c***, ***11***, and ***13***

The reactions of *N*-phenylpiperidinethiosemicarbazone derivative **5** (0.005 moles) with α-halo-carbonyl derivatives **6a–g** or **8a–c** or **10** or **12** (0.005 moles) were carried out in dioxane (30 mL) with the addition of Et3N (0.005 moles) under the conditions of reflux for a duration of 6 h. TLC was used to track the progression of the reactions, and the results showed that only one derivative was produced in each case. Following the completion of each reaction, the colorful generated solid was collected using filtration, washed with methanol, and then crystallized from a mixture of dioxane and ethanol.

#### 2.2.1. 4-Methyl-5-(phenyldiazenyl)-2-(2-(1-(4-(piperidin-1-yl)phenyl)ethylidene-hydrazinyl) thiazole (**7a**)

Red solid (88% yield), mp 182–184 °C (Dioxane), IR (KBr) ν 3572 (NH), 2931 (CH), 1504 (C=N), 1242, 1172 cm^−1^. ^1^ H NMR (DMSO-d_6_): δ 1.17–1.75 (m, 4H, 2CH_2_), 2.47 (s, 3H, CH_3_), 2.59 (s, 3H, CH_3_), 3.39–3.57 (m, 6H, 3CH_2_), 6.98–7.94 (m, 9H, Ar-H), 10.53 (s, 1H, NH). MS *m*/*z* (%): 418 (M^+^, 22), 401 (10), 378 (20), 372 (13), 356 (13), 340 (13), 320 (12), 293 (20), 270 (21), 237 (26), 219 (13), 197 (21), 183 (21), 170 (14), 166 (16), 156 (23), 151 (19), 139 (17), 134 (8), 128 (11), 117 (20), 109 (10), 93 (17), 76 (15), 66 (39), 60 (47), 43 (100). Anal. calcd for C_23_H_26_N_6_S (418.56): C, 66.00; H, 6.26; N, 20.08. Found: C, 66.18; H, 6.06; N, 20.18%.

#### 2.2.2. 4-Methyl-2-(2-(1-(4-(piperidin-1-yl)phenyl)ethylidene)hydrazinyl)-5-(p-tolyldiazenyl)-thiazole (**7b**)

Brown solid (89% yield), mp 230–232 °C (Dioxane), IR (KBr) ν 3441 (NH), 2924, (CH), 1512 (C=N), 1242, 1180, 1026 cm^−1^. ^1^ H NMR (DMSO-d_6_): δ 1.59–1.79 (m, 4H, 2CH_2_), 2.26 (s, 3H, CH_3_), 2.44 (s, 3H, CH_3_), 2.57 (s, 3H, CH_3_), 3.43 (br., 6H, 3CH_2_), 6.93–7.88 (m, 9H, Ar-H, NH). MS *m*/*z* (%):432 Anal. calcd for C_24_H_28_N_6_S (432.58): C, 66.64; H, 6.52; N, 19.43. Found: C, 66.44; H, 6.41; N, 19.29%.

#### 2.2.3. 5-((4-Chlorophenyl)diazenyl)-4-methyl-2-(2-(1-(4-(piperidin-1-yl)phenyl)ethylidene)-hydrazinyl)thiazole (**7c**)

Red solid (90% yield), mp 174–176 °C (Dioxane), IR (KBr) ν 3217 (NH), 2924, (CH), 1581 (C=N), 1519, 1381, 1242, 1172, 1103, 1010 cm^−1^. ^1^ H NMR (DMSO-d_6_): δ 1.23–1.79 (m, 4H, 2CH_2_), 2.45 (s, 3H, CH_3_), 2.58 (s, 3H, CH_3_), 3.57 (br, 6H, 3CH_2_), 6.94 (d, Hz = 9 Hz, 2H, Ar-H), 7.35 (s, 4H, Ar-H), 7.82 (d, Hz = 9 Hz, 2H, Ar-H), 10.54 (s, 1H, NH). ^13^C NMR (DMSO-d_6_) δ 14.80, 16.42, 23.97, 25.00, 48.26, 113.89, 115.64, 120.67, 125.81, 128.32, 129.11, 139.25, 142.68, 152.84, 164.40, 169.50, 176.88, MS *m*/*z* (%): 455 (M^+^+2, 2), 454 (M^+^+1, 3), 453 (M^+^, 6), 444 (31), 440 (19), 424 (26), 415 (62), 4411 (76), 405 (20), 400 (23), 398 (26), 391 (79), 367 (13), 370 (38), 366 (37), 361 (50), 356 (34), 353 (50), 346 (50), 336 (100), 307 (67), 303 (80), 3291 (65), 272 (44), 214 (50), 197 (35), 181 (55), 176 (17), 139 (50), 127 (25), 111 (28), 103 (42), 85 (31), 76 (18), 61 (83). Anal. calcd for C_23_H_25_ClN_6_S (453.00): C, 60.98; H, 5.56; N, 18.55. Found: C, 60.89; H, 5.38; N, 18.70%.

#### 2.2.4. 4-Methyl-5-((4-nitrophenyl)diazenyl)-2-(2-(1-(4-(piperidin-1-yl)phenyl)ethylidene)-hydrazinyl)thiazole (**7d**)

Red solid (88% yield), mp 160–162 °C (Dioxane), IR (KBr) ν 3470–3610 (NH), 2924, (CH), 1589 (C=N), 1527, 1319, 1249, 1111, 1002 cm^−1^. ^1^H NMR (DMSO-d_6_): δ 1.60 (s, 4H, 2CH_2_), 2.46 (s, 3H, CH_3_), 2.61 (s, 3H, CH_3_), 3.56 (s, 6H, 3CH_2_), 6.95 (d, Hz = 9.3 Hz, 2H, Ar-H), 7.46 (d, Hz= 9 Hz, 2H, Ar-H), 7.84 (d, Hz = 9 Hz, 2H, Ar-H), 8.19 (d, Hz = 9.3 Hz, 2H, Ar-H), 11.04 (s, 1H, NH). ^13^C NMR (DMSO-d_6_): δ 15.33, 17.00, 24.41, 25.40, 48.62, 114.19, 114.26, 125.88, 126.23, 128.94, 141.31, 143.29, 149.80, 153.39, 165.79, 169.32, 177.81. MS *m*/*z* (%):463 (M^+^, 10), 455 (57), 445 (32), 411 (43), 386 (100), 371 (60), 338 (43), 312 (27), 287 (45), 270 (50), 258 (30), 253 (66), 245 (39), 239 (48), 220 (24), 208 (37), 194 (32), 186 (36), 134 (57), 102 (23), 77 (11), 82 (74), 69 (47). Anal. calcd for C_23_H_25_N_7_O_2_S (463.56): C, 59.59; H, 5.44; N, 21.15. Found: C, 59.69; H, 5.32; N, 21.31%.

#### 2.2.5. 4-Methyl-2-(2-(1-(4-(piperidin-1-yl)phenyl)ethylidene)hydrazinyl)-5-(m-tolyldiazenyl)-thiazole (**7e**)

Red solid (90% yield), mp 208–210 °C (Dioxane), IR (KBr) ν 3209 (NH), 3100, 2924, (CH), 1597 (C=N), 1519, 1435, 1373, 1327, 1257, 1180, 1118, 1010 cm^−1^. ^1^H NMR (CDCl_3_): δ 1.64–1.76 (m, 6H, 3CH_2_), 2.37 (s, 3H, CH_3_), 2.53 (s, 3H, CH_3_), 2.64 (s, 3H, CH_3_), 3.71 (t, 4H, 2CH_2_), 6.82 (d, J = 8 Hz, 2H, Ar-H), 6.99–7.27 (m, 4H, Ar-H), 7.60 (s, 1H, NH), 7.89 (d, J = 8 Hz, 2H, Ar-H). ^13^C NMR (CDCl_3_): δ 15.09, 16.64, 21.55, 24.18, 25.30, 49.33, 111.32, 114.55, 114.60, 123.37, 127.31, 128.48, 129.16, 139.25, 140.61, 142.83, 152.82, 165.18, 169.31, 176.84. MS *m*/*z* (%): 432 (M^+^, 46), 429 (32), 424 (57), 413 (36), 397 (46), 392 (37), 348 (22), 327 (33), 299 (40), 239 (39), 265 (84), 257 (84), 248 (22), 227 (72), 212 (53), 199 (40), 193 (40), 182 (44), 180 (40), 162 (25), 153 (60), 141 (64), 122 (34), 113 (100), 68 (86). Anal. calcd for C_24_H_28_N_6_S (432.58): C, 66.64; H, 6.52; N, 19.43. Found: C, 66.43; H, 6.48; N, 19.39%.

#### 2.2.6. 5-((3-Chlorophenyl)diazenyl)-4-methyl-2-(2-(1-(4-(piperidin-1-yl)phenyl)ethylidene)-hydrazinyl)thiazole (**7f**)

Red solid (87% yield), mp 220–222 °C (Dioxane), IR (KBr) ν 3433 (NH), 2924, (CH), 1566 (C=N), 1512, 1342, 1211, 1149, 1010 cm^−1^. ^1^H NMR (CDCl_3_): δ 1.37–1.77 (m, 4H, 2CH_2_), 2.52 (s, 3H, CH_3_), 2.64 (s, 3H, CH_3_), 3.06–3.71 (m, 6H, 3CH_2_), 6.95–7.90 (m, 9H, Ar-H, NH). ^13^C NMR (DMSO-d_6_) δ 9.03, 15.28, 24.41, 25.41, 48.67, 113.21, 113.91, 114.32, 121.75, 126.12, 128.81, 131.39, 134.31, 140.33, 145.60, 153.31, 165.09, 169.75, 177.50. MS *m*/*z* (%): 455 (M^+^+2, 15), 454 (M^+^+1, 10), 453 (M^+^, 44), 441 (28), 433 (56), 419 (50), 394 (45), 359 (33), 311 (28), 283 (57), 270 (34), 240 (35), 211 (100), 207 (45), 196 (62), 175 (42), 149 (45), 143 (39), 127 (36), 107 (23), 86 (57), 77 (36), 66 (77). Anal. calcd for C_23_H_25_ClN_6_S (453.00): C, 60.98; H, 5.56; N, 18.55. Found: C, 60.87; H, 5.39; N, 18.61%.

#### 2.2.7. 4-Methyl-5-((3-nitrophenyl)diazenyl)-2-(2-(1-(4-(piperidin-1-yl)phenyl)ethylidene)-hydrazinyl)thiazole (**7g**)

Reddish brown solid (85% yield), mp 210–212 °C (Dioxane), IR (KBr) ν 3410 (NH), 3093, 2924, (CH), 1597 (C=N), 1527, 1342, 1234, 1172, 1010 cm^−1^. ^1^H NMR (CDCl_3_): δ 1.33–1.78 (m, 4H, 2CH_2_), 2.51 (s, 3H, CH_3_), 2.66 (s, 3H, CH_3_), 3.07–3.71 (m, 6H, 3CH_2_), 7.03 (s, 1H, NH), 7.42–8.34 (m, 8H, Ar-H, NH). ^13^C NMR (DMSO-d_6_) δ 9.03, 15.26, 24.40, 25.41, 48.65, 108.57, 114.27, 116.21, 120.36, 126.06, 128.82, 131.08, 141.35, 145.31, 149.02, 153.32, 165.31, 169.60, 177.48. MS *m*/*z* (%): 463 (M^+^, 38), 454 (85), 449 (79), 423 (55), 407 (45), 401 (82), 383 (62), 355 (16), 325 (90), 310 (50), 305 (45), 255 (37), 234 (43), 194 (84), 175 (86), 157 (26), 114 (65), 102 (40), 81 (100), 68 (60). Anal. calcd for C_23_H_25_N_7_O_2_S (463.56): C, 59.59; H, 5.44; N, 21.15. Found: C, 59.65; H, 5.42; N, 21.21%.

#### 2.2.8. 2-Ethoxy-2-oxo-N’-phenylacetohydrazonic-2-(1-(4-(piperidin-1-yl)phenyl)ethylidene)-hydrazinecarbimidic thioanhydride (**9a**)

Yellow solid (85% yield), mp 204–206 °C (Dioxane), IR (KBr) ν 3263 (br, NH), 3055, 2931, (CH), 1735 (CO), 1581 (C=N), 1504, 1442, 1373, 1195, 1095, 1026 cm^−1^. ^1^H NMR (CDCl_3_): δ 1.40 (t, J = 7 Hz, 3H, CH_3_), 1.62–1.74 (d, 6H, 3CH_2_), 2.13 (s, 3H, CH_3_), 3.10–3.70 (m, 4H, 2CH_2_), 4.43 (q, J = 7 Hz, 2H, CH_2_), 6.80 (s, 1H, NH), 6.97 (d, J = 8 Hz, 2H, Ar-H), 7.72 (s, 1H, NH), 7.46 (d, J = 8 Hz, 2H, Ar-H), 7.73–7.85 (m, 5H, Ar-H), 8.19 (s, 1H, NH). ^13^C NMR (CDCl_3_): δ 12.09, 14.21, 23.99, 25.26, 50.02, 61.76, 113.11, 115.64, 125.46, 126.74, 127.95, 128.85, 138.55, 148.33, 151.81, 153.84, 159.96, 166.55. MS *m*/*z* (%): 466 (M^+^, 24), 457 (20), 454 (15), 440 (45), 431 (37), 423 (29), 413 (27), 403 (25), 398 (38), 395 (30), 383 (28), 373 (26), 359 (27), 341 (31), 325 (100), 311 (42), 302 (40), 258 (49), 242 (30), 226 (20), 87 (24), 166 (68), 91 (75), 77 (64), 66 (40). Anal. calcd for C_24_H_30_N_6_O_2_S (466.60): C, 61.78; H, 6.48; N, 18.01. Found: C, 61.90; H, 6.35; N, 18.18%.

#### 2.2.9. 2-Ethoxy-2-oxo-N’-(p-tolyl)acetohydrazonic-2-(1-(4-(piperidin-1-yl)phenyl)ethylidene)-hydrazinecarbimidic thioanhydride (**9b**)

Buff solid (83% yield), mp 178–200 °C (Dioxane), IR (KBr) ν 3263 (br, NH), 3070, 2924, (CH), 1735 (CO), 1589 (C=N), 1519, 1458, 1373, 1327, 1195, 1111, 1018 cm^−1^. ^1^ H NMR (CDCl_3_): δ 1.26 (t, J = 7 Hz, 3H, CH_3_), 1.63–2.13 (m, 4H, 2CH_2_), 2.30 (s, 3H, CH_3_), 2.45 (s, 3H, CH_3_), 3.12–3.70 (m, 6H, 3CH_2_), 4.36 (q, J = 7 Hz, 2H, CH_2_), 6.82 (s, 1H, NH), 7.02 (d, J = 8 Hz, 2H, Ar-H), 7.12 (d, J = 8 Hz, 2H, Ar-H), 7.25 (d, J = 8 Hz, 2H, Ar-H), 7.38 (d, J = 8 Hz, 2H, Ar-H), 7.79 (s, 1H, NH), 11.23 (s, 1H, NH). ^13^C NMR (DMSO-d_6_) δ 13.39, 14.54, 21.18, 24.36, 25.44, 49.22, 61.39, 114.53, 115.06, 125.59, 127.08, 129.77, 130.25, 140.69, 148.91, 151.87, 154.96, 160.27, 163.40. MS *m*/*z* (%): 480 (M^+^, 8), 478 (36), 470 (39), 459 (46), 452 (68), 432 (56), 413 (53), 403 (50), 392 (43), 372 (90), 361 (50), 354 (64), 348 (28), 334 (80), 317 (41), 311 (44), 286 (28), 282 (46), 271 (48), 264 (50), 247 (42), 242 (51), 232 (45), 219 (77), 208 (51), 173 (100), 161 (42), 154 (86), 151 (63), 143 (49), 135 (42), 129 (34), 121 (46), 119 (32), 107 (80), 103 (50), 96 (22), 88 (52), 76 (17), 68 (53), 57 (84). Anal. calcd for C_25_H_32_N_6_O_2_S (480.63): C, 62.47; H, 6.71; N, 17.49. Found: C, 62.58; H, 6.67; N, 17.55%.

#### 2.2.10. N’-(4-Chlorophenyl)-2-ethoxy-2-oxoacetohydrazonic-2-(1-(4-(piperidin-1-yl)phenyl)-ethylidene)hydrazinecarbimidic thioanhydride (**9c**)

Buff solid (81% yield), mp 170–172 °C (Dioxane), IR (KBr) ν 3441, 3263 (br, NH), 3090, 2931, (CH), 1728 (CO), 1597 (C=N), 1504, 1381, 1219, 1103, 1018 cm^−1^. ^1^H NMR (CDCl_3_): δ 1.37 (t, J = 7 Hz, 3H, CH_3_), 1.66–1.8 (m, 4H, 2CH_2_), 2.15 (s, 3H, CH_3_), 2.51–3.70 (m, 6H, 3CH_2_), 4.43 (q, J = 7 Hz, 2H, CH_2_), 6.96 (d, J = 8 Hz, 2H, Ar-H), 7.07 (d, J = 8 Hz, 2H, Ar-H), 7.24 (d, J = 8 Hz, 2H, Ar-H), 7.45 (s, 1H, NH), 7.76 (d, J = 8 Hz, 2H, Ar-H), 8.50 (s, 1H, NH), 12.15 (s, 1H, NH). ^13^C NMR (DMSO-d_6_) δ 9.00, 14.53, 24.35, 25.42, 49.19, 61.49, 114.54, 115.77, 126.95, 127.77, 128.27, 129.36, 143.34, 151.92, 155.04, 160.15, 162.16, 163.78. MS *m*/*z* (%): 503 (M^+^+2, 4), 502 (M^+^+1, 3), 501 (M^+^, 15), 472 (34), 456 (78), 395 (25), 387 (30), 345 (23), 298 (24), 258 (100), 232 (35), 214 (23), 145 (32), 102 (33), 87 (24), 71 (22). Anal. calcd for C_24_H_29_ClN_6_O_2_S (501.04): C, 57.53; H, 5.83; N, 16.77. Found: C, 57.77; H, 5.92; N, 16.68%.

#### 2.2.11. 4-(4-Chlorophenyl)-2-(2-(1-(4-(piperidin-1-yl)phenyl)ethylidene)hydrazinyl)thiazole (**11**)

Brown solid (82% yield), mp 242–244 °C (Dioxane), IR (KBr) ν 3441 (br, NH), 3086, 2924, (CH), 1597 (C=N), 1527, 1450, 1388, 1319, 1219, 1095, 1018 cm^−1^. ^1^ H NMR (DMSO-d_6_): δ 1.15 (t, J = 7 Hz, 1H, CH-aliph), 1.57 (s, 3H, CH &CH_2_ aliph), 2.25 (s, 3H, CH_3_), 3.04–3.56 (m, 6H, 3CH_2_ aliph), 6.91(d, J = 8 Hz, 2H, Ar-H), 7.33 (s, 1H, NH), 7.44 (d, J = 8 Hz, 2H, Ar-H), 7.56 (s, 1H, thiazole-H), 7.61 (d, J = 8 Hz, 2H, Ar-H), 7.89 (d, J = 8 Hz, 2H, Ar-H). ^13^C NMR (DMSO-d_6_) δ 14.25, 24.41, 25.47, 49.24, 104.92, 113.37, 115.11, 127.13, 127.67, 129.06, 130.67, 132.25, 134.26, 147.56, 152.09, 170.64. MS *m*/*z* (%): 412 (M^+^+2, 2), 411 (M^+^+1, 1), 410 (M^+^, 5), 391 (12), 378 (91), 376 (54), 374 (50), 317 (24), 303 (38), 273 (95), 269 (100), 254 (34), 246 (13), 231 (21), 213 (31), 186 (22), 183 (27), 106 (18), 81 (32). Anal. calcd for C_22_H_23_ClN_4_S (410.96): C, 64.30; H, 5.64; N, 13.63. Found: C, 64.48; H, 5.46; N, 13.44%.

#### 2.2.12. 1-(4-Methyl-2-(2-(1-(4-(piperidin-1-yl)phenyl)ethylidene)hydrazinyl)thiazol-5-yl)ethanone (**13**)

Brown solid (80% yield), mp 212–214 °C (Dioxane), IR (KBr) ν 3441 (br, NH), 3078, 2931, (CH), 1680 (CO), 1604 (C=N), 1512, 1527, 1442, 1365, 1311, 1234, 1111, 1026 cm^−1^. ^1^ H NMR (CDCl_3_): δ 1.38–1.72 (m, 6H, 3CH_2_ aliph), 2.22 (s, 3H, CH_3_), 2.46 (s, 3H, CH_3_), 2.58 (s, 3H, CH_3_), 3.08–3.69 (m, 4H, 2CH_2_ aliph), 6.91 (d, J = 8 Hz, 2H, Ar-H), 7.17 (s, 1H, NH), 7.68 (d, J = 8 Hz, 2H, Ar-H). ^13^C NMR (DMSO-d_6_) δ 9.06, 14.43, 24.41, 25.28, 25.44, 29.91, 49.10, 113.37, 114.95, 127.25, 127.48, 152.36, 189.23. MS *m*/*z* (%): 356 (M^+^, 53), 353 (47), 348 (38), 346 (36), 344 (47), 337 (43), 324 (45), 299 (40), 281 (100), 271 (27), 265 (20), 250 (33), 238 (43), 214 (25), 187 (17), 180 (34), 155 (19), 136 (31), 128 (16), 113 (16), 105(19), 92 (42), 85 (56), 77 (42), 74 (37), 60 (62). Anal. calcd for C_19_H_24_N_4_OS (356.49): C, 64.01; H, 6.79; N, 15.72. Found: C, 64.20; H, 6.90; N, 15.66%.

### 2.3. In Vitro Assessment of VEGFR2 Enzyme

The target compounds were screened in vitro against VEGFR2 kinase using VEGFR2 kinase assay kit, Bioscience (San Diego, CA, USA). VEGFR2 Kinase Assay Kit is designed to measure VEGFR2 kinase activity for screening applications using Kinase-Glo^®^ MAX as a detection reagent. Sunitinib was utilized as the positive control. In brief, VEGFR2 enzyme, substrate, ATP, and assay buffer were added to the plate wells, treated with different concentrations of the test compounds/positive control, and incubated at 30 °C for 45 min. Then, Kinase-Glo^®^ MAX reagent was added, and the plate was incubated at room temperature for 15 min. Luminescence was measured using a microplate reader. The IC_50_ values were calculated from the concentration–inhibition response curve.

### 2.4. In Vitro Assessment of Cytotoxicity

Target compounds were screened against A498 renal cancer cells and WI38 non-cancerous normal cells using in vitro Toxicological MTT-Based Assay Kit, Sigma (Saint Louis, MO, USA). Sunitinib was selected as the positive control. Briefly, A498 and WI38 cells were grown in DMEM supplemented with streptomycin, penicillin, and FBS. The cells were kept under CO_2_ (5%) at 37 °C. The cells were washed with PBS, collected, plated, and incubated overnight under CO_2_ (5%) at 37 °C. The tested cells were treated with different concentrations of the target compounds and positive control and incubated for 48 h at 37 °C. After that, the MTT reagent was added to plate wells and cells were re-incubated for 4 h under dark conditions. The absorbance was measured by a plate reader and cell viability was estimated. The IC_50_ values were obtained from the concentration-inhibition curve (*n* = 3) [24,25,26,27].

### 2.5. Cell Cycle Analysis and Apoptotic Study

Cell cycle analysis was performed using Propodium Iodide Flow Cytometry Kit for Cell Cycle Analysis, abcam (Cambridge, UK). Briefly, A498 renal cancer cells were treated with **7c** at its IC_50_ value and incubated for 24 h. The cells were collected, washed with PBS, and fixed with ethanol. The cells were stained with propidium iodide. The distribution of cells in each phase of cell cycle was calculated using a flow cytometer.

Apoptotic study was conducted using Annexin V-FITC Apoptosis Detection Kit, Biovision (Mountain View, CA, USA). Shortly, A498 renal cancer cells were treated with the IC_50_ value of **7c** and incubated for 24 h. The cells were collected, washed with PBS, and fixed with ethanol. The cells were stained with Annexin V-FITC and propodium iodide. The cells were analyzed using a flow cytometer [28,29].

### 2.6. Docking Protocol

A high-resolution VEGFR2 kinase co-crystallized with sunitinib (PDB: 4AGD) was retrieved from a protein data bank website [30]. The target compounds were docked into the active site of VEGFR2 using Molecular Operating Environment MOE software. First, to prepare the protein for docking, water molecules were removed from the crystal structure of VEGFR2 and hydrogen atoms were added. The target protein was subjected to energy minimization. The binding site of the target protein was identified. The investigated compounds were drawn on MOE and subjected to energy minimization too. Finally, the target compounds were docked into the binding pocket of VEGFR2 enzyme and the binding scores were calculated.

## 3. Results and Discussion

**Scheme 1:**
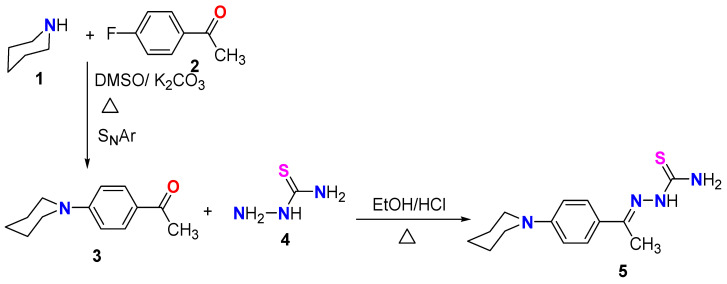
Synthesis of thiosemicarbazone **5**.

In a continuation of our work in the synthesis of reactive thiosemicarbazone derivatives [31,32,33], and as shown in Scheme 1, our research started with the synthesis of the starting *N*-phenylpiperidinethiosemicarbazone **5** via the reaction of piperidine **1** with 4-fluoroacetophenepone according to the published procedure to produce 1-(4-piperidin-1-yl-phenyl)-ethanone (**3**) [34]. The acetyl derivative **3** was reacted with thiosemicarbazide (**4**) according to the literature to produce the final product thiosemicarbazone derivative **5** [35].

**Scheme 2:**
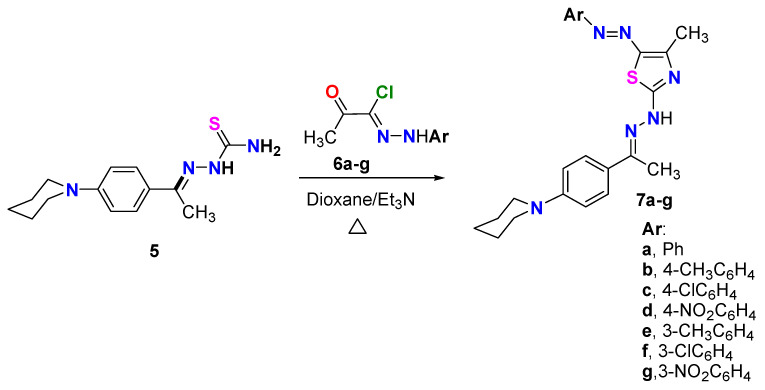
Synthesis of thiazole derivatives **7a–g**.

The reaction of *N*-phenylpiperidinethiosemicarbazone derivative **5** with hydrazonoyl chlorides **6a–g** (the hydrazonoyl halides **6a–g** were prepared according to reported literature [23]) in dioxane in a basic medium using Et_3_N under reflux for 6 h resulted in the synthesis of a novel series of thiazoles incorporating an *N*-phenylpiperidine fragment. TLC analysis was performed on all the reaction mixtures, and the results showed that each reaction produced only a single product (Scheme 2). Based on the spectral data of products **7a–g**, as well as the elemental analyses of those products, the thiazole structure of those compounds was determined. For instance, the ^1^H NMR spectrum of compound **7e** indicated the presence of three CH_3_ groups at δ = 2.37, 2.53 and 2.64 ppm, in addition to 5CH_2_ groups appearing as a multiple and a triplet at δ = 1.64 (3CH_2_) and 3.71 (2CH_2_) ppm, respectively. The eight aromatic protons and one NH appeared at δ = 6.83–7.92 ppm. Also, the disappearance of the NH_2_ signal in ^1^H NMR spectra of all derivatives **7a–g** was evidence of the formation of such products. The ^13^C NMR spectrum of the same derivative **7e** showed 20 carbon signals: six signals were located in the aliphatic region for 3CH_3_ and 3 CH_2_ at δ = 15.09, 16.64, 21.55, 24.18, 25.30, and 49.33 ppm; and 14 carbon signals for the thiazole and aromatic rings at δ =111.32, 114.55, 114.60, 123.37, 127.31, 128.48, 129.16, 139.25, 140.61, 142.83, 152.82, 165.18, 169.31, 176.84. Another tool for evidence of the suggested structure was the IR data for all synthesized series that revealed NH absorption bands near 3400 cm^−1^.

**Scheme 3:**
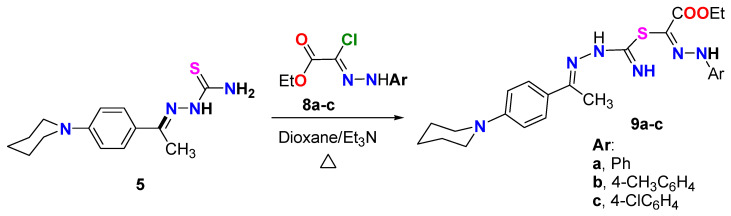
Reaction of thiosemicarbazone derivative **5** with hydrazonoyl chlorides **8a–c**.

Under the same reaction condition, *N*-phenylpiperidine-thiosemicarbazone derivative **5** was treated with ester-hydrazonoyl chloride derivatives **8a–c** (Scheme 3), but in this case the reaction proceeded to produce the uncyclized derivatives **9a–c** and all attempts to cyclize such products failed. The structure of the formed derivatives **9a–c** was confirmed based on spectral data and elemental analyses. For instance, the IR spectrum of derivative **9a** revealed the characteristic absorption band for the carbonyl ester C=O at 1735 cm^−1^. Moreover, the ^1^H NMR spectrum of the same derivative **9a** showed the triplet and quartet of the CH_3_CH_2_ group of the ester moiety at 1.40 and 4.43 ppm, in addition to the aliphatic and aromatic protons as follows: 1.62 (d, 3CH_2_), 2.13 (s, CH_3_), 3.10–3.70 (m, 3CH_2_), 6.97 (d, Ar-H), 7.46 (dAr-H), 7.73–7.85 (m, Ar-H), 6.80, 7.72, 8.19 (3s, 3NH) ppm. ^13^C NMR spectrum of **9a** showed 18 carbon signals at δ = 12.09 (CH_3_-ester), 14.21 (CH_3_), 23.99, 25.26, 50.02 (5CH_2_-piperidine), 61.76 (CH_2_-ester), 113.11, 115.64, 125.46, 126.74, 127.95, 128.85, 138.55, 148.33, 151.81, 153.84, 159.96 (3C=N and aromatic C), 166.55 (C=O ester) ppm. It is important to note that the formation of the open-chain **9a–c** ester derivatives will have promising activity as anticancer agents. This activity could be attributed to the presence of nitrogen atoms (=N and NH) and the ester group, which are known to have the ability to bind within cancer cell proteins.

**Scheme 4:**
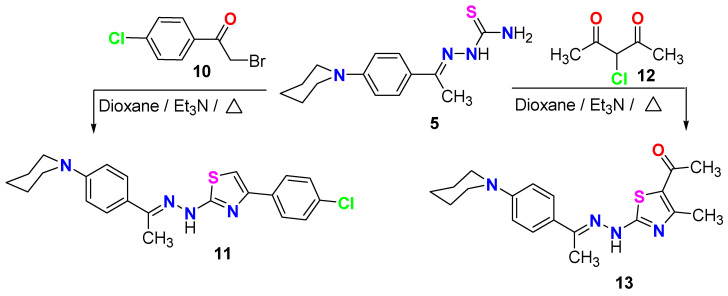
Reaction of thiosemicarbazone derivative **5** with phenacyl bromide derivative **10** and α-chloroacetyl acetone **12**.

Scheme 4 illustrates the synthesis of two new thiazole compounds, **11** and **13**, through the reaction of *N*-phenylpiperidine-thiosemicarbazone derivative **5** with p-chlorophenacyl bromide **10** and α-chloroacetyl acetone **12** in refluxing dioxane, utilizing a stoichiometric amount of Et_3_N. The spectral data presented in the experimental section confirmed the structures of the two new thiazole derivatives, **11** and **13**.

### 3.1. In Vitro Assessment of VEGFR2 Enzyme

In the present work, the newly synthesized compounds were evaluated in vitro for their ability to inhibit the kinase activity of VEGFR2 enzyme. Sunitinib was used as a reference standard.

As shown in Table 1, the tested compounds exhibited varying degrees of VEGFR2 enzyme inhibition with IC_50_ values spanning from 0.049 to 1.868 µM, relative to sunitinib (IC_50_ = 0.118 ± 0.003 µM). Except for compounds **7a** and **7f**, all tested compounds inhibited VEGFR2 at sub-micromolar levels ranging from 0.049 to 0.858 µM. Among the tested compounds, **7c** (IC_50_ = 0.073 ± 0.002 µM), **9b** (IC_50_ = 0.049 ± 0.002 µM), and **9c** (IC_50_ = 0.093 ± 0.003 µM) were the most potent, showing VEGFR2 enzyme inhibition superior to that of sunitinib. In addition, compounds **7b** (IC_50_ = 0.134 ± 0.004 µM) and **7d** (IC_50_ = 0.13 ± 0.004 µM) were nearly equipotent to sunitinib. Compounds **7g** (IC_50_ = 0.279 ± 0.009 µM) and **13** (IC_50_ = 0.181 ± 0.006 µM) showed moderate enzyme inhibition. Mild VEGFR2 inhibitory activity was observed for compounds **7e** (IC_50_ = 0.762 ± 0.024 µM) and **9a** (IC_50_ = 0.6 ± 0.019 µM).

**Figure 5:**
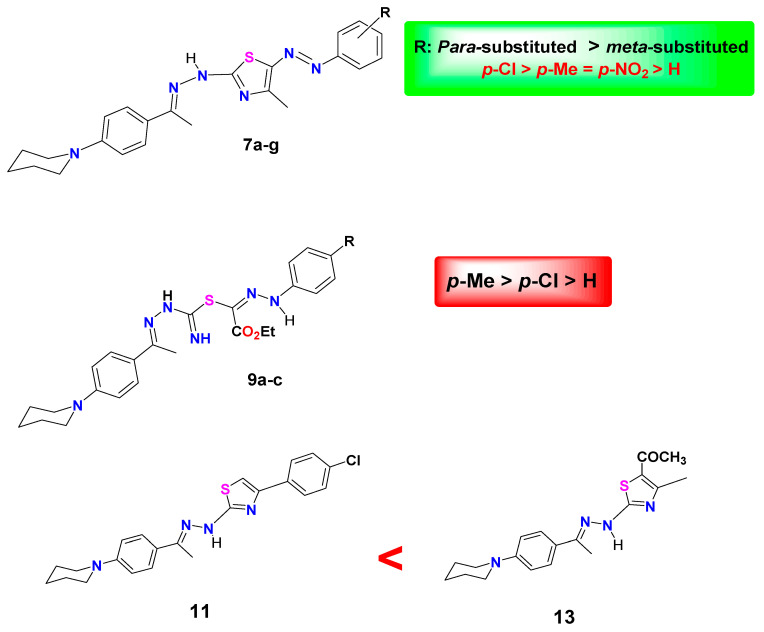
Summary of SAR regarding VEGFR2 inhibition.

Regarding 5-arylazothiazole derivatives **7a–g**, it was observed that the VEGFR2 kinase inhibitory activity was remarkably affected by the nature and the position of substituents on phenyl ring (**R**). As clearly observed, the introduction of substituent in *para* position of phenyl ring (compounds **7b**, **7c**, **7d**) resulted in an increase in VEGFR2 inhibitory activity, as compared to the unsubstituted derivative **7a**, in the following order: *p*-Cl > *p*-Me = *p*-NO_2_ > H. Among the *para*-substituted derivatives, the *para-*chloro analog **7c** (IC_50_ = 0.073 ± 0.002 µM) was the most potent showing enzyme inhibition superior to that of sunitinib (IC_50_ = 0.118 ± 0.003 µM). On the contrary, the introduction of a substituent in the *meta* position of phenyl ring led to a great decline in VEGFR2 inhibitory activity. The *meta-*substituted derivatives **7e**, **7f**, and **7g** were much less potent than their corresponding *para-*substituted analogs **7b**, **7c**, and **7d**, respectively (Figure 5).

On the other hand, the ethyl carboxylate derivatives **9a**, **9b**, and **9c** displayed potent VEGFR2 inhibitory activity in the following order: *p*-Me > *p*-Cl > H. The *para*-methyl **9b** (IC_50_ = 0.049 ± 0.002 µM) and the *para*-chloro **9c** (IC_50_ = 0.093 ± 0.003 µM) analogs were 2.4-fold and 1.2-fold more potent than sunitinib (IC_50_ = 0.118 ± 0.003 µM) against VEGFR2 enzyme, respectively. Concerning thiazole derivatives **11** and **13**, the 4-chlorophenyl thiazole **11** inhibited VEGFR2 enzyme at an IC_50_ value of 0.858 ± 0.027 µM, whereas a much better (about 4-fold) activity was observed for the 4-methyl-5-acetylthiazole **13** (IC_50_ = 0.181 ± 0.006 µM) (Figure 5).

### 3.2. In Vitro Assessment of Cytotoxicity

As mentioned before, given the highly vascularized nature of renal cell carcinoma, sunitinib has been among the tyrosine kinase inhibitors approved by FDA as a first-line treatment for renal cell carcinomas. The effectiveness of sunitinib against renal cell carcinomas has been mainly attributed to the inhibition of VEGFR [10,11]. With this in mind, the most potent compounds in the VEGFR2 enzyme assay, namely **7c**, **9b**, and **9c** were further assessed in vitro for their ability to inhibit the growth of renal cancer cells A498, using sunitinib as a reference drug. As seen in Table 2, the *para*-chlorophenylazothiazole derivative **7c** was the most active against A498 cells exhibiting one-digit IC_50_ value of 7.866 ± 0.27 µM, relative to sunitinib (IC_50_ = 2.955 ± 0.1 µM). While compounds **9b** and **9c** were less active and showed two-digit IC_50_ values against A498 cells with the *para*-chlorophenylhydrazinyl derivative **9c** (IC_50_ = 17.81 ± 0.6 µM) being more potent than its *para*-methyl analog (IC_50_ = 22.67 ± 0.77 µM). Although compounds **7c**, **9b**, and **9c** exhibited excellent inhibitory activity against VEGFR2 in the enzymatic assay, their IC_50_ values against A498 renal cancer cells were noticeably higher than those observed at the enzyme level. These discrepancies between enzyme-based inhibition and cellular activity are common in drug discovery. This phenomenon is driven by the huge difference between the simple isolated environment in enzyme assay and the complex environment of a living cell. A high affinity of an inhibitor for the enzyme does not guarantee cellular efficacy due to different contributing factors, such as membrane permeability, substrate concentration, metabolic stability, protein binding, intracellular target engagement, co-factor and co-substrate availability, or non-specific cytotoxicity [36,37].

### 3.3. In Vitro Assessment of Cytotoxicity Against Normal Non-Cancerous Cells

In this work, the most promising compound **7c** was subjected to further evaluation against normal non-cancerous WI38 cells. As presented in Table 2, compound **7c**, with an IC_50_ value of 65.28 ± 2.31 µM, demonstrated a potentially improved safety profile against WI38 cells in comparison to sunitinib (IC_50_ = 24.93 ± 0.87 µM).

### 3.4. Cell Cycle Analysis

**Figure 6:**
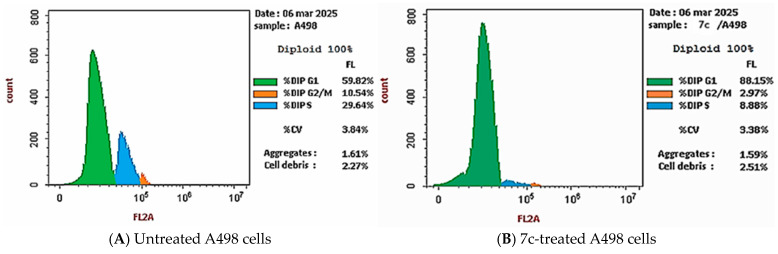
Cell cycle analyses of untreated (**A**) and **7c**-treated (**B**) A498 cells.

A further investigation was conducted to determine the impact of compound **7c** on the percentage of cells in different phases of cell cycle. As shown in Table 3 and Figure 6, the treatment of A498 renal cancer cells with compound **7c** resulted in an obvious increase in the percentage of cells in the G0–G1 phase, which increased from 60% to 88%. Additionally, the percentage of cells in both the S and G2–M phases decreased by approximately 60% compared to the untreated A498 cells, resulting in cell cycle arrest at the G1 phase.

### 3.5. Apoptotic Study

**Figure 7:**
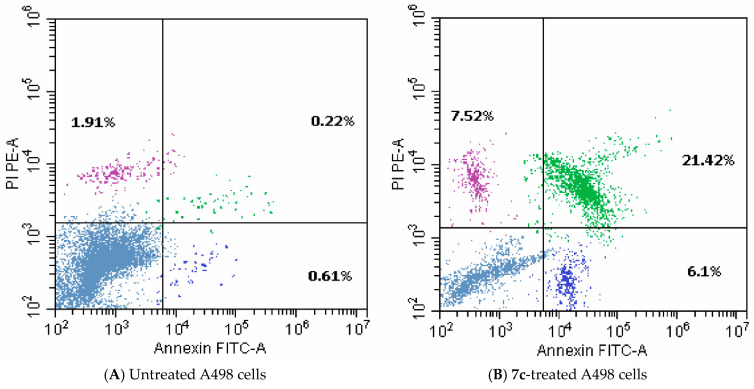
Flow cytometric analysis of apoptosis and necrosis in untreated (**A**) and **7c**-treated (**B**) A498 cells.

As displayed in Table 4 and Figure 7, the treatment of A498 renal cancer cells with compound **7c** resulted in a significant (33-fold) elevation in overall apoptosis, relative to the untreated cells. Almost a four-fold increase in necrotic cells from 1.91% to 7.52% was also detected. Thus, compound **7c** could be considered as an apoptotic inducer.

### 3.6. Docking Study

**Figure 8:**
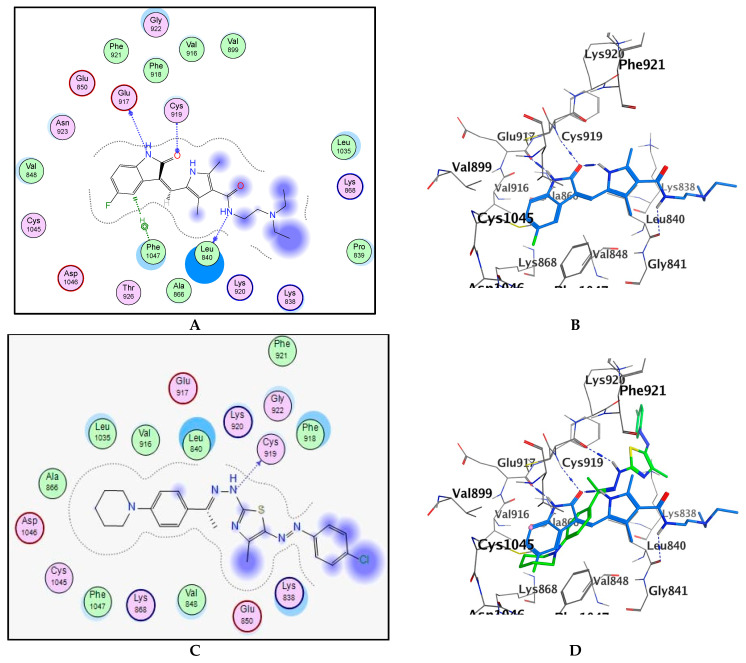
Sunitinib docked into the active site of VEGFR2 enzyme: 2D (**A**), 3D (**B**), compound **7c** docked into the active site of VEGFR2 enzyme (**C**), overlay of **7c** (green) and sunitinib (blue) docked into the active site of VEGFR2 enzyme (**D**).

To gain an insight into the binding interactions of target compounds with VEGFR2 enzyme, the most promising compound **7c** was docked into the active site of VEGFR2 kinase using MOE (Molecular Operating Environment) (2014.0901) software. A high-resolution VEGFR2 kinase co-crystallized with sunitinib (PDB: 4AGD) was utilized in docking study [29]. First, for validation purposes, the co-crystallized sunitinib was re-docked into the binding site of VEGFR2 (PDB: 4AGD). The docked sunitinib reproduced a binding mode similar to that of the co-crystalized ligand with an RMSD=1.43. The NH group and the carbonyl oxygen of indolinone core in sunitinib formed two hydrogen bonds with the hinge residues Glu917 and Cys919, respectively. Also, the NH group of the side chain donated a hydrogen bond to Leu840. In addition, the indolinone core of sunitinib participated in hydrophobic interactions with Leu840, Ala866, the gatekeeper Val916, and Leu1035. Regarding compound **7c**, it was properly oriented into the active site of VEGFR2 enzyme with the docking score = –6.7 kcal/mol, relative to sunitinib (–6.9 kcal/mol). Compound **7c** revealed a binding pose similar to that of sunitinib. Compound **7c** was stabilized into the active site of VEGFR2 via hydrogen bonding as well as hydrophobic interactions. As proposed in our design, the hydrazinyl NH in **7c** donated a hydrogen bond to the hinge region key residue Cys919. In addition, the piperidinylphenyl fragment was properly oriented into a hydrophobic pocket lined with Leu840, Val848, Ala866, the gate keeper Val916, Leu1035, and Phe1047. This hydrophobic interaction could probably enhance the binding affinity of **7c** towards VEGFR2 and therefore could rationalize its potent VEGFR2 inhibitory activity (Figure 8).

## 4. Conclusions

Finally, in this article we synthesized a novel series of 4-piperidinylphenyl-linked thiazoles as VEGFR2 inhibitors with potential cytotoxic activity against renal cancer. The structures of all synthesized 4-piperidinylphenyl-linked thiazoles were proved based on their spectral and elemental data. Target compounds were screened against VEGFR2 enzyme. Most of the target compounds inhibited VEGFR2 enzyme at sub-micromolar IC_50_ values. Compounds **7c** (IC_50_ = 0.073 ± 0.002 µM), **9b** (IC_50_ = 0.049 ± 0.002 µM), and **9c** (IC_50_ = 0.093 ± 0.003 µM) were the most potent, showing VEGFR2 inhibition superior to that of the sunitinib reference drug (IC_50_ = 0.118 ± 0.003 µM). Moreover, compounds **7c**, **9b**, and **9c** effectively inhibited the growth of A498 renal cancer cells, with compound **7c** being the most potent showing one-digit IC_50_ value of 7.866 ± 0.27 µM. In addition, compound **7c** revealed a potentially improved safety profile against non-cancerous normal cells compared to sunitinib. The treatment of A498 renal cancer cells with compound **7c** resulted in an apparent cell cycle arrest at the G1 phase as well as a significant induction of apoptosis. A docking study was also conducted. Compound **7c** was properly oriented and stabilized into the active site of VEGFR2 via hydrogen bonding and hydrophobic interactions with the key residues were located in the binding pocket of VEGFR2. Supplementary Figures S1–S51: represent the H NMR, C NMR, IR, Mass charts of the new synthesized derivatives. Also, it contains the biological reports in pages 43–49.

## Figures and Tables

**Table 1:** In vitro assessment of target compounds against VEGFR2 enzyme. VEGFR2 enzyme was treated with different concentrations of test compounds. IC50 values were calculated from the concentration–inhibition response curve.

Code	VEGFR2 IC_50_ µM
**7a**	1.030 ± 0.032
**7b**	0.134 ± 0.004
**7c**	0.073 ± 0.002
**7d**	0.130 ± 0.004
**7e**	0.762 ± 0.024
**7f**	1.868 ± 0.058
**7g**	0.279 ± 0.009
**9a**	0.600 ± 0.019
**9b**	0.049 ± 0.002
**9c**	0.093 ± 0.003
**11**	0.858 ± 0.027
**13**	0.181 ± 0.006
**Sunitinib**	0.118 ± 0.003

**Table 2:** In vitro assessment of cytotoxicity. Cells were treated with five different concentrations of test compound. IC50 values were calculated from the concentration-inhibition response curve, n = 3.

Code	Cytotoxicity IC_50_ µM	
	A498	WI38
**7c**	7.866 ± 0.27	65.280 ± 2.31
**9b**	22.670 ± 0.77	----
**9c**	17.810 ± 0.6	---
**Sunitinib**	2.955 ± 0.1	24.930 ± 0.87

**Table 3:** Cell cycle analysis of untreated and **7c**-treated A498 cells.

Code	DNA Content		
	%G0-G1	%S	%G2/M
**7c/A498**	88.15	8.88	2.97
**cont. A498**	59.82	29.64	10.54

**Table 4:** Flow cytometric analysis of apoptosis and necrosis in untreated and **7c**-treated A498 cells.

Code	Apoptosis			Necrosis
	Early	Late	Total	
**7c/A498**	6.100	21.420	27.520	7.520
**cont. A498**	0.610	0.220	0.830	1.910

## Data Availability

The original contributions presented in this study are included in the article/Supplementary Material. Further inquiries can be directed to the corresponding author(s).

## Supplementary Materials

The following supporting information can be downloaded at: https://www.mdpi.com/article/10.3390/biom16030370/s1.
