# Supplementary material for: New Derivatives of 4-Piperidinylphenyl-Linked Thiazoles as VEGFR2 Inhibitors with Potential Cytotoxicity Against Renal Cancer
Source: Biomolecules. 2026 Feb 28;16(3):370. doi: 10.3390/biom16030370 (PMC13023839; doi:10.3390/biom16030370)
Supplement: Supplementary file 1 [file biomolecules-16-00370-s001.zip › biomolecules-4153155-SupM.pdf]

Figure

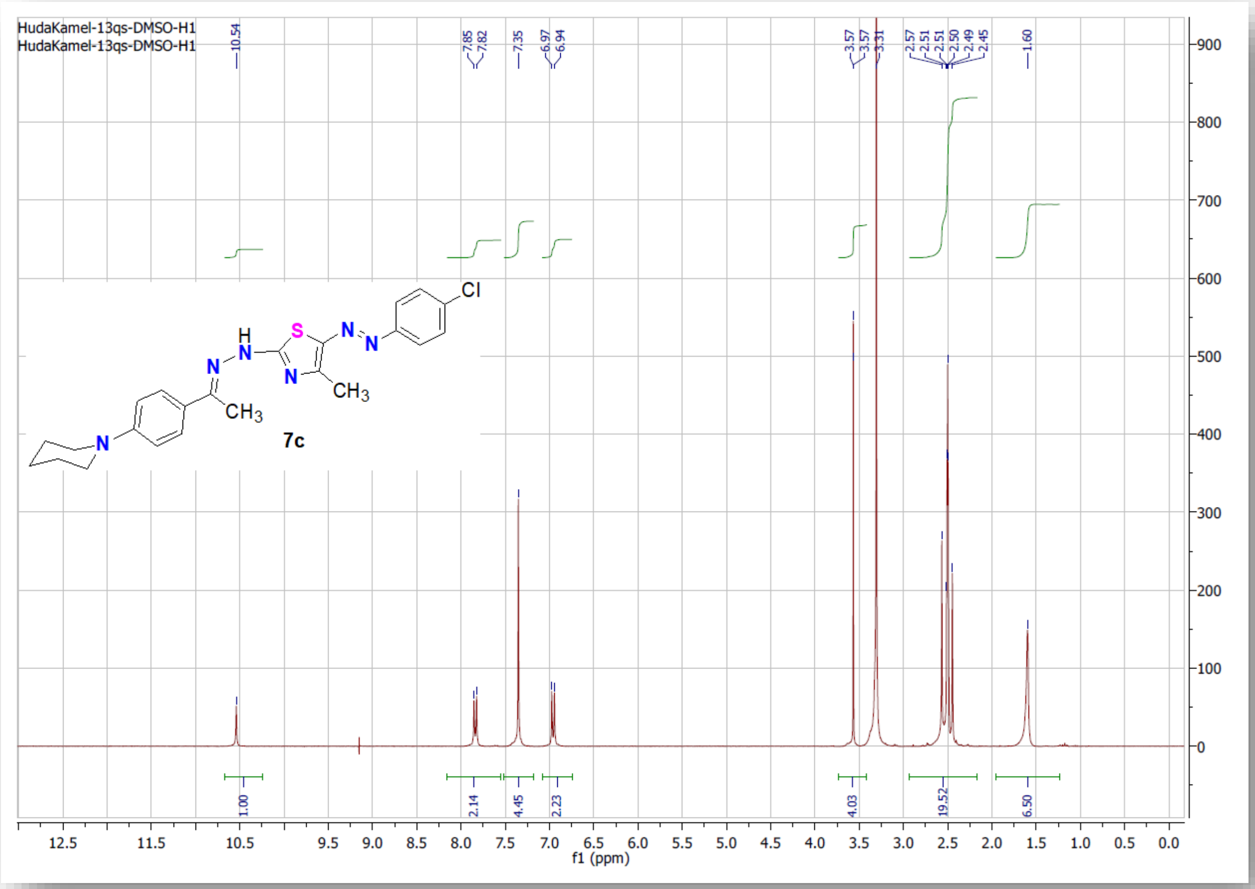

Figure S1. ^{1}H NMR spectrum of compound **7c.**

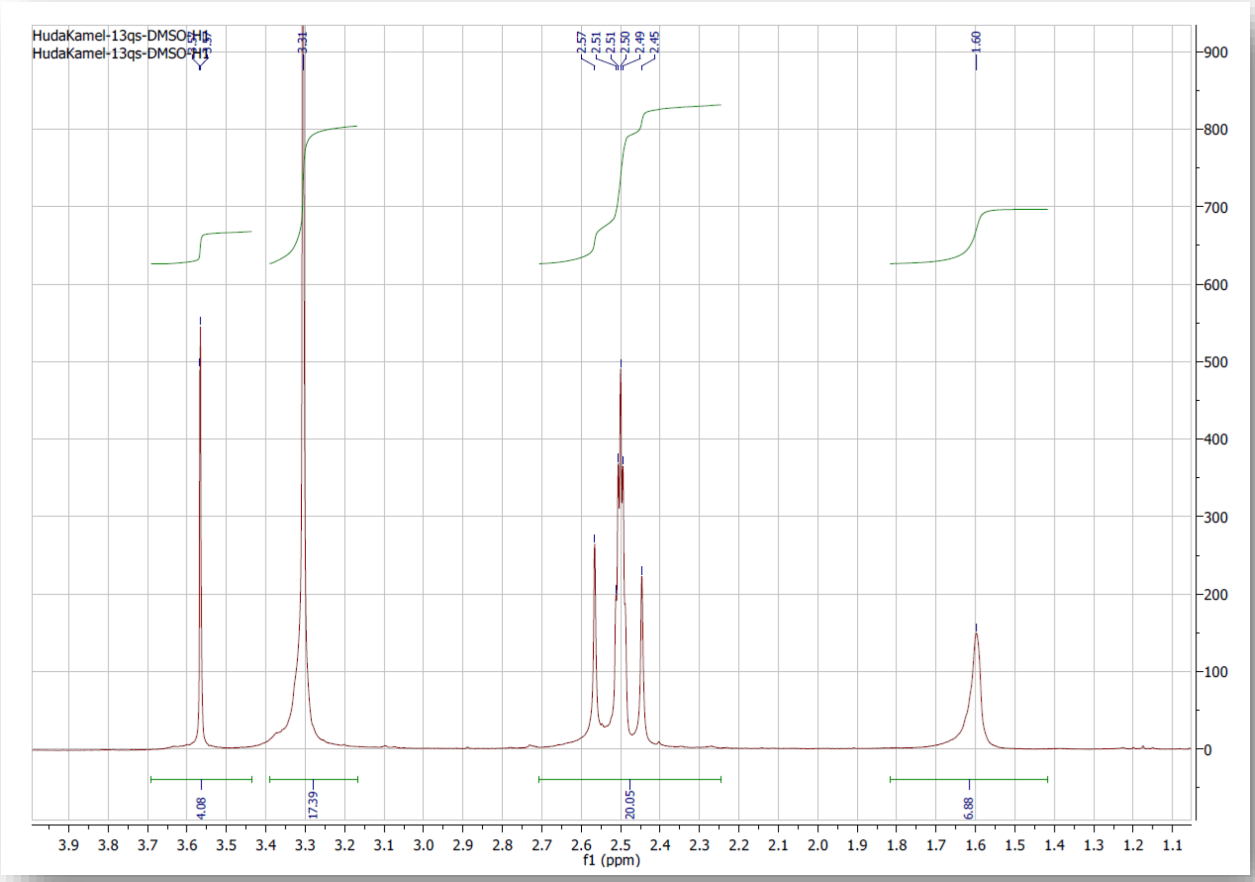

Figure S2. ^{1}H NMR spectrum of compound **7c.**

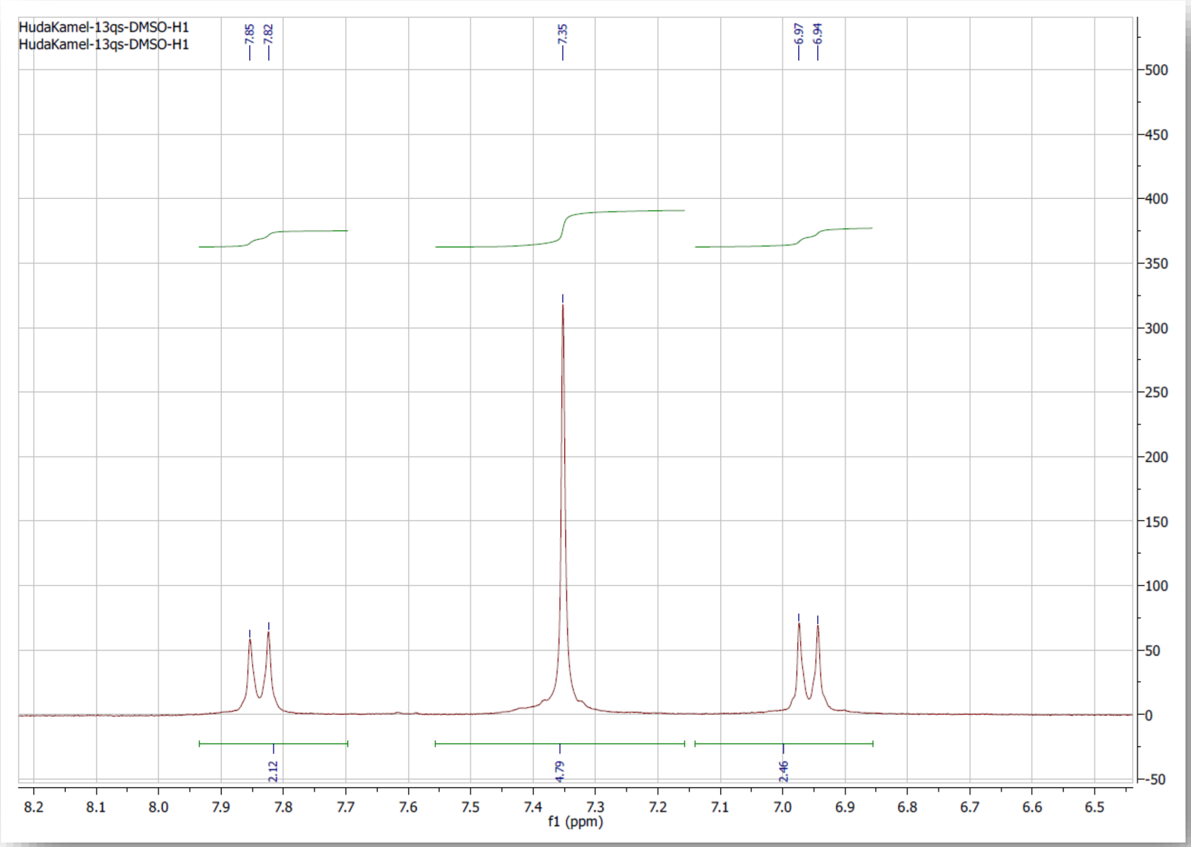

Figure S3. ^{1}H NMR spectrum of compound **7c**

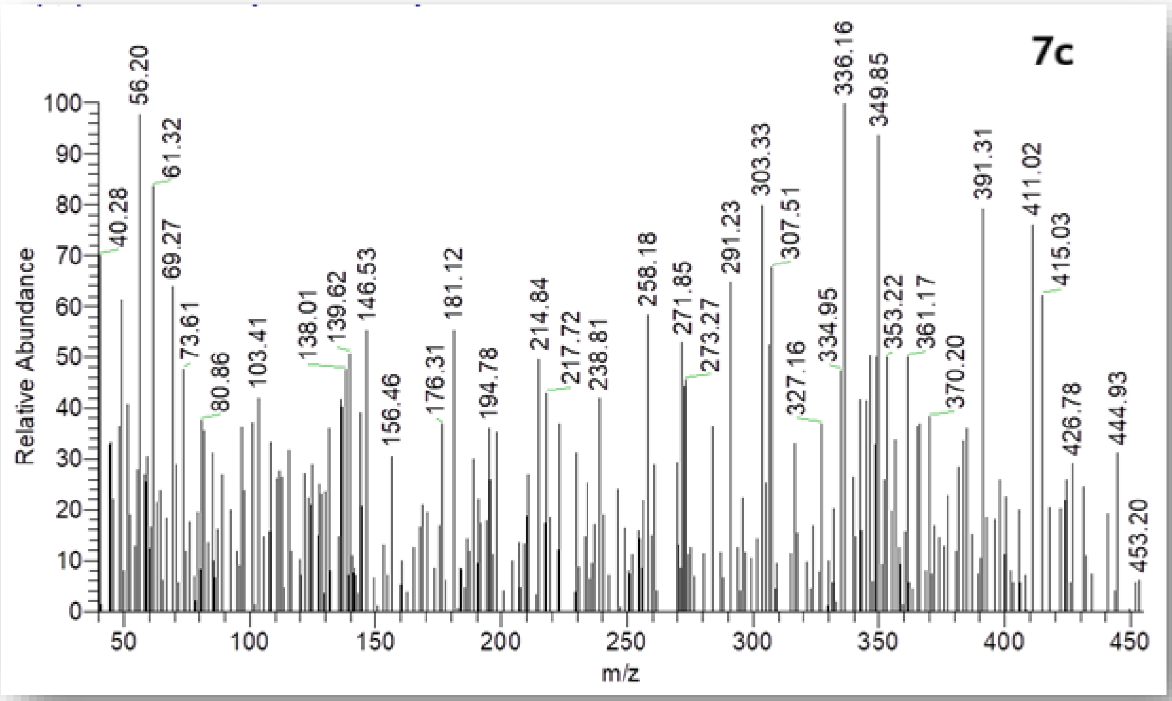

Figure S4. Mass spectrum of compound **7c**

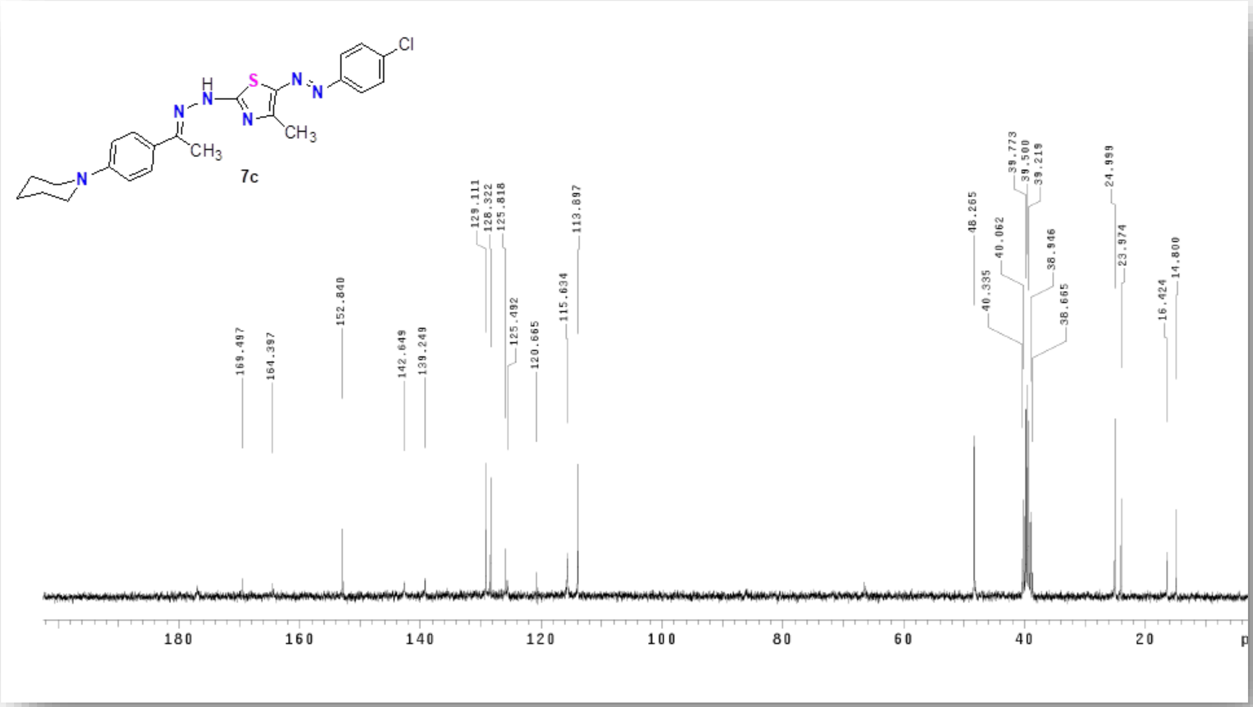

Figure S5. ^{13}C NMR of compound **7c**

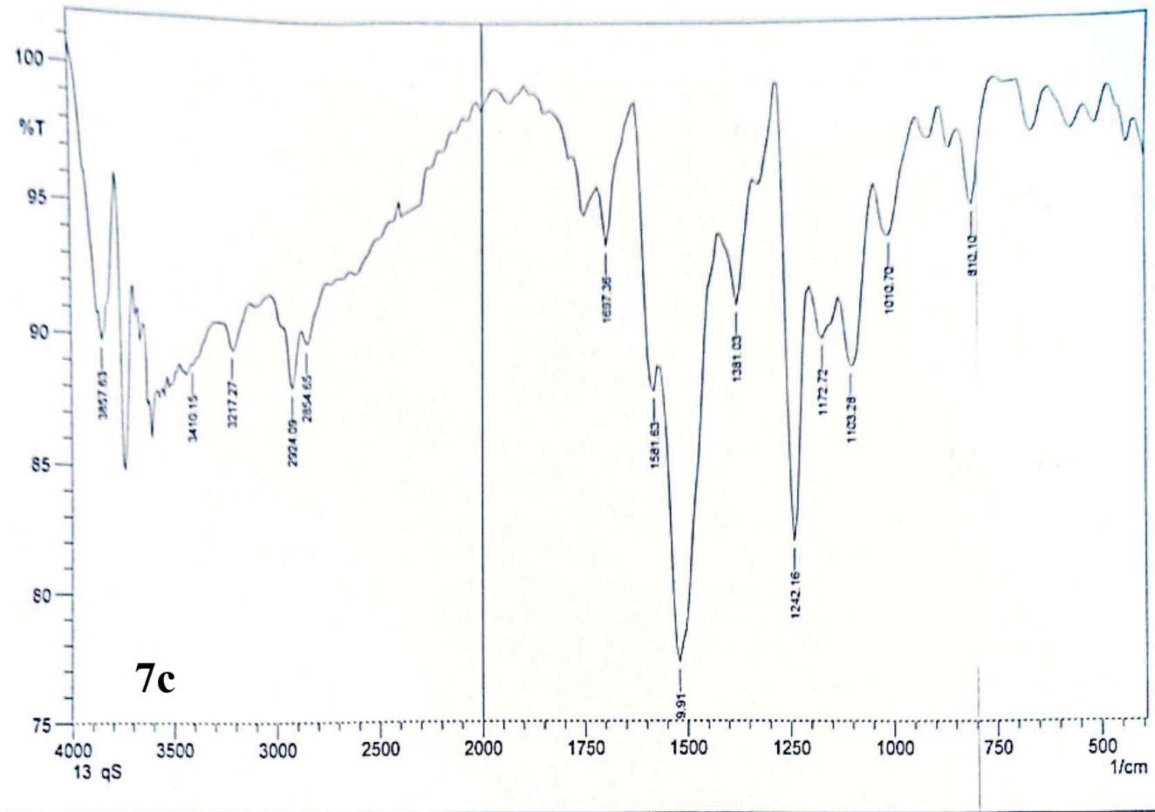

	Peak	Intensity	Corr. Intensity	Base (H)	Base (L)	Area	Corr. Area
1	810.1	94.881	3.645	848.68	756.1	1.074	0.54
2	1010.7	93.768	2.898	1049.28	948.98	2.007	0.656
3	1103.28	88.772	4.199	1134.14	1049.28	3.401	0.937
4	1172.72	89.836	1.804	1203.58	1134.14	2.978	0.337
5	1242.16	81.934	13.416	1288.45	1203.58	3.974	2.325
6	1381.03	91.088	3.861	1427.32	1342.46	2.608	0.659
7	1519.91	77.249	13.125	1566.2	1427.32	10.064	4.604
8	1581.63	87.729	2.412	1627.92	1573.91	2.041	0.499
9	1697.36	93.407	3.026	1720.5	1627.92	1.499	0.369
10	2854.65	89.603	0.89	2877.79	2762.06	4.896	0.218
11	2924.09	87.864	2.63	3032.1	2885.51	6.833	0.683
12	3217.27	89.39	1.316	3255.84	3132.4	5.497	0.368
13	3410.15	88.842	0.133	3417.86	3309.85	5.113	0.046
14	3857.63	89.779	7.7	4004.22	3788.19	5.572	3.875

Figure S6. IR spectrum of compound **7c**

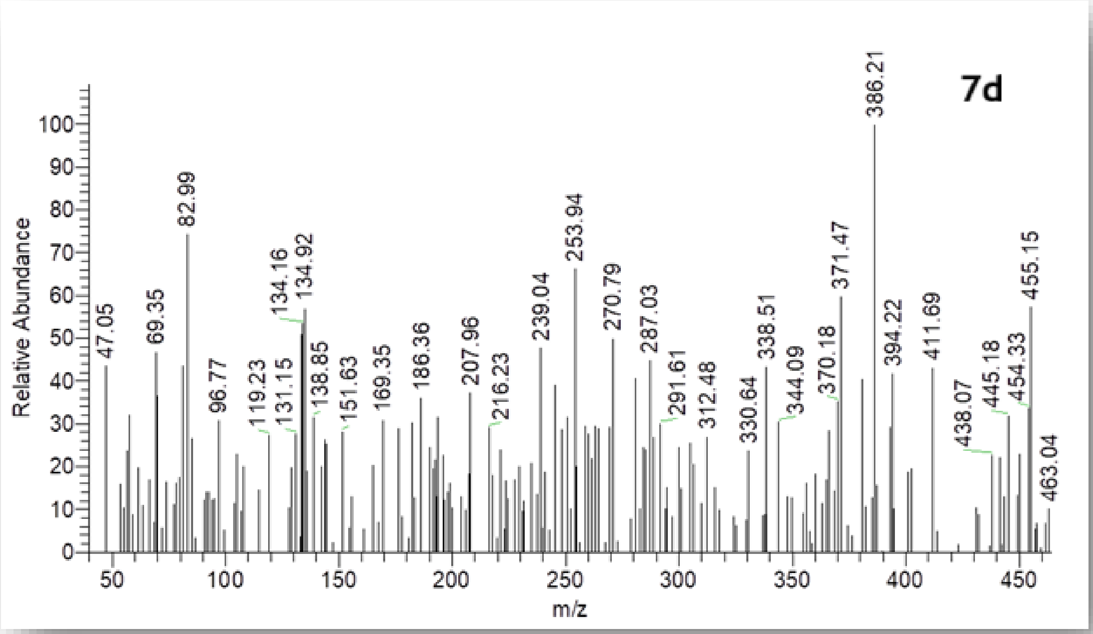

Figure S7. Mass spectrum of compound **7d**

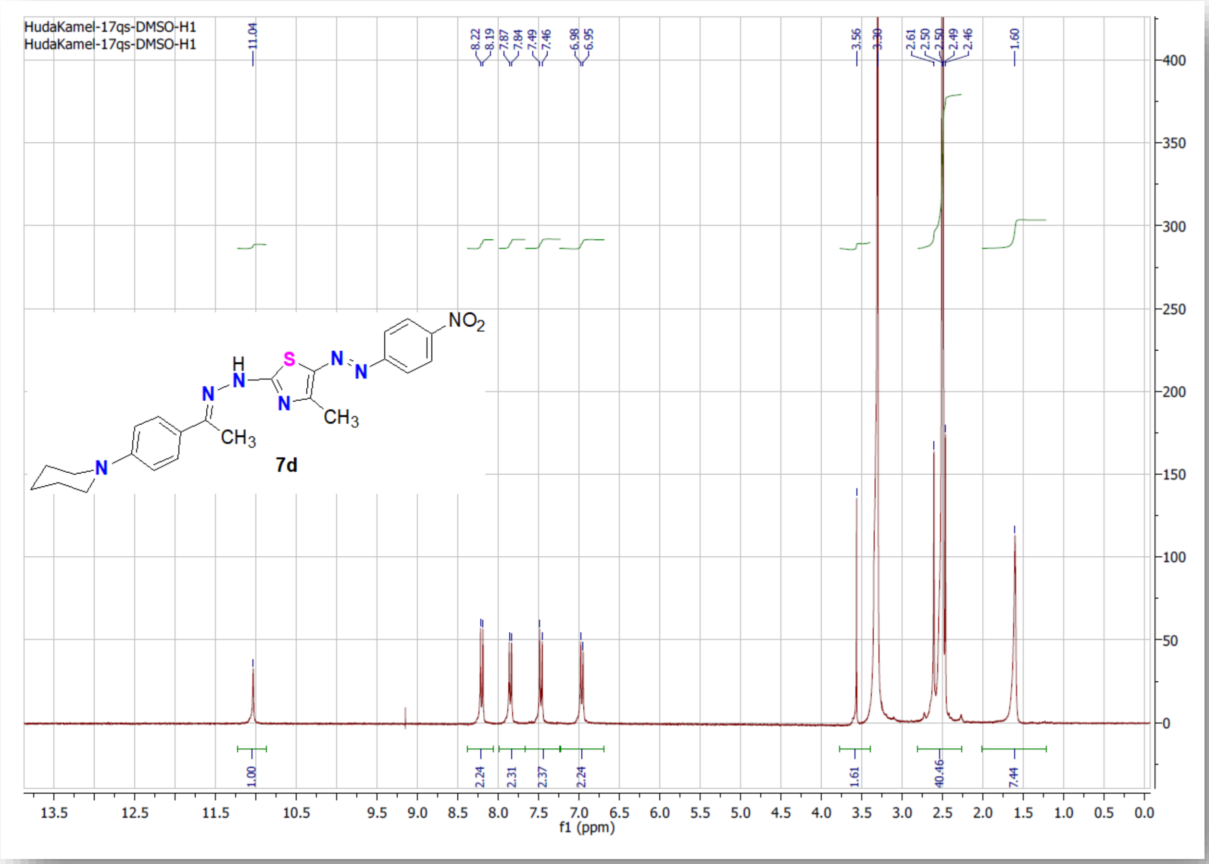

Figure S8. ^{1}H NMR spectrum of compound **7d**

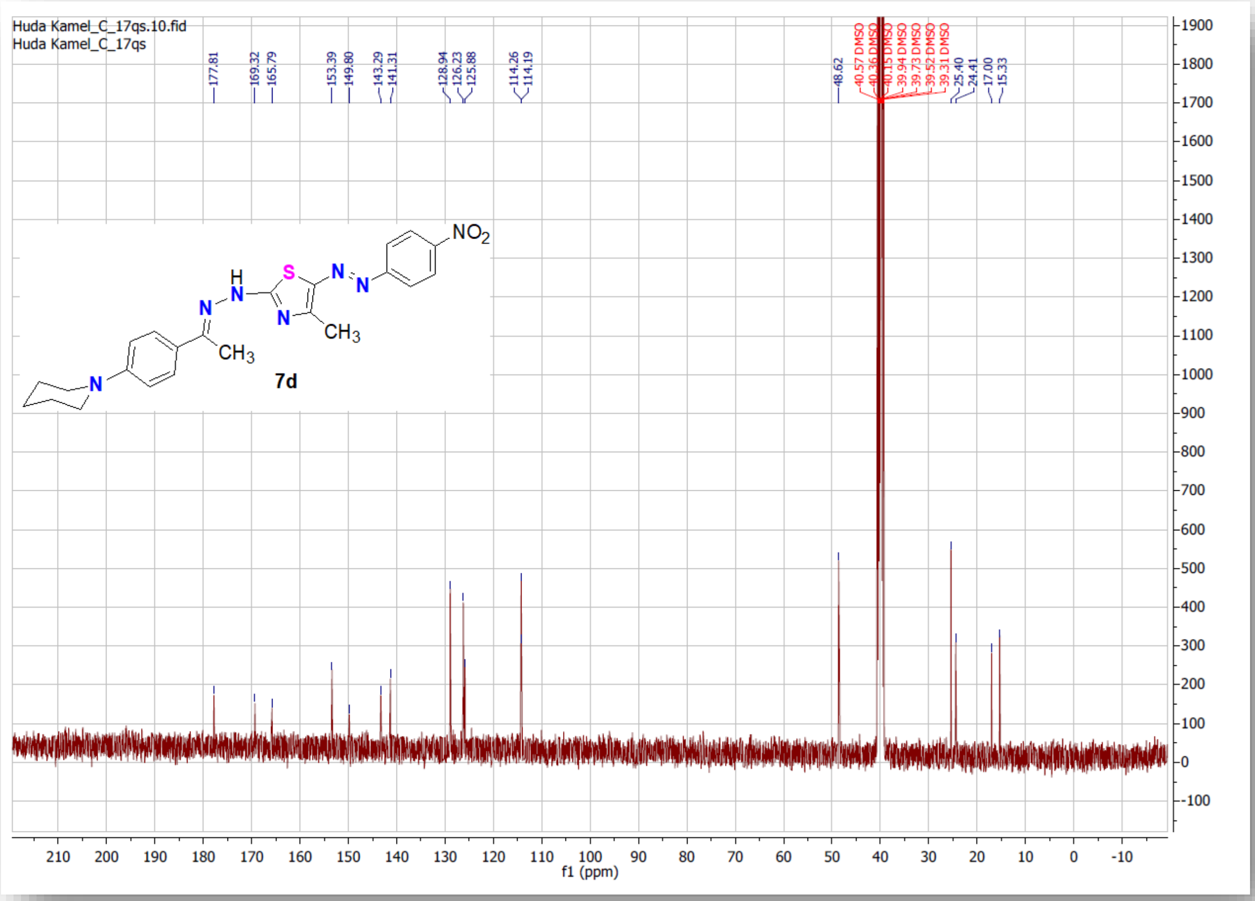

Figure S9. ^{13}C NMR spectrum of compound **7d.**

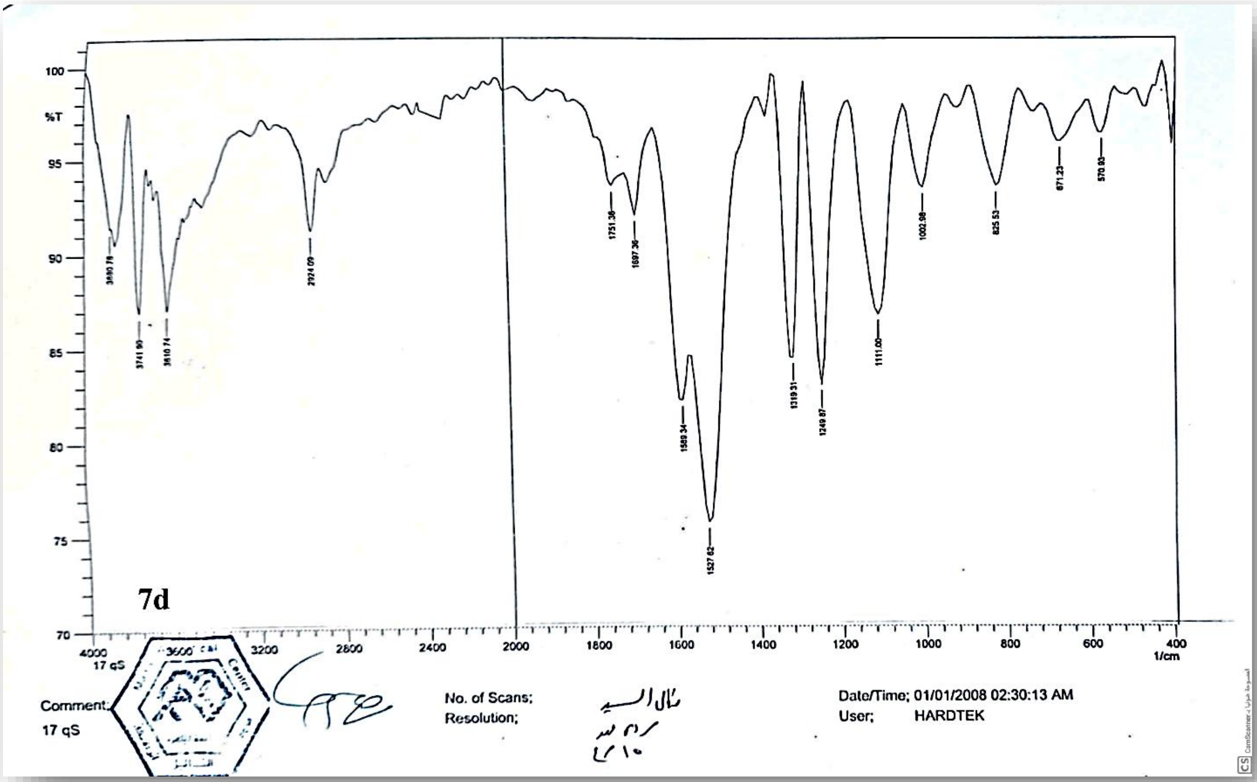

Figure S10. IR spectrum of compound **7d**

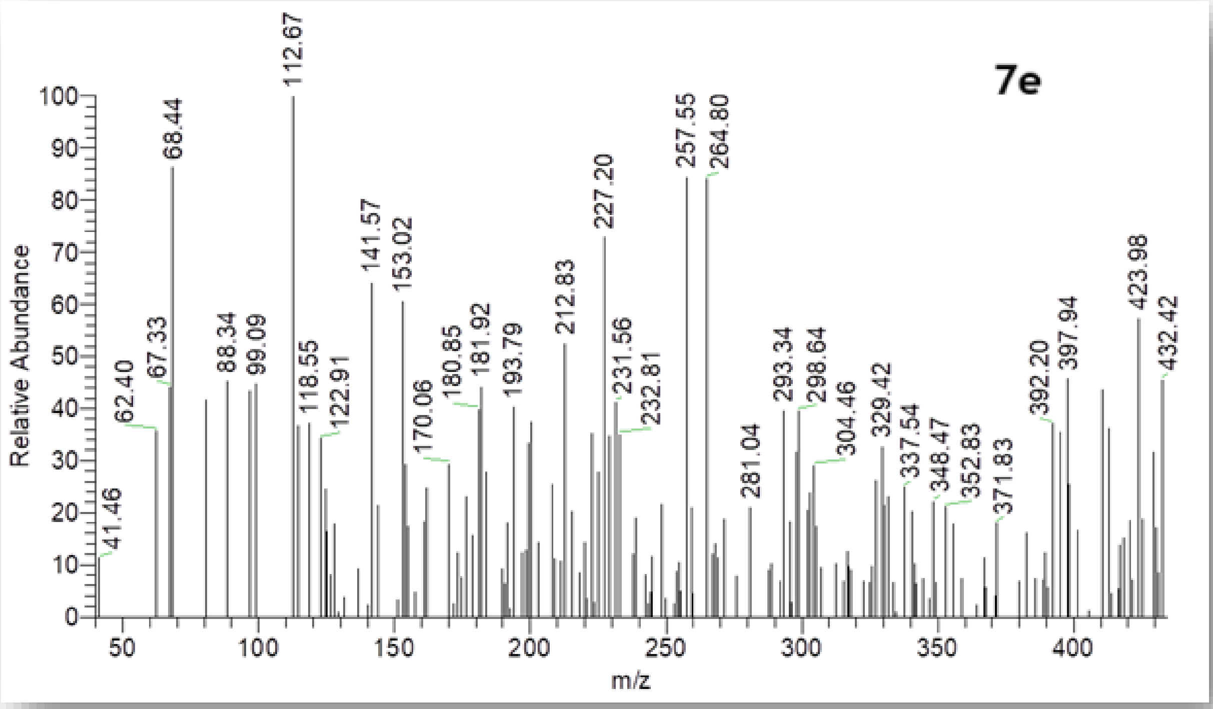

Figure S11. Mass spectrum of compound **7e**

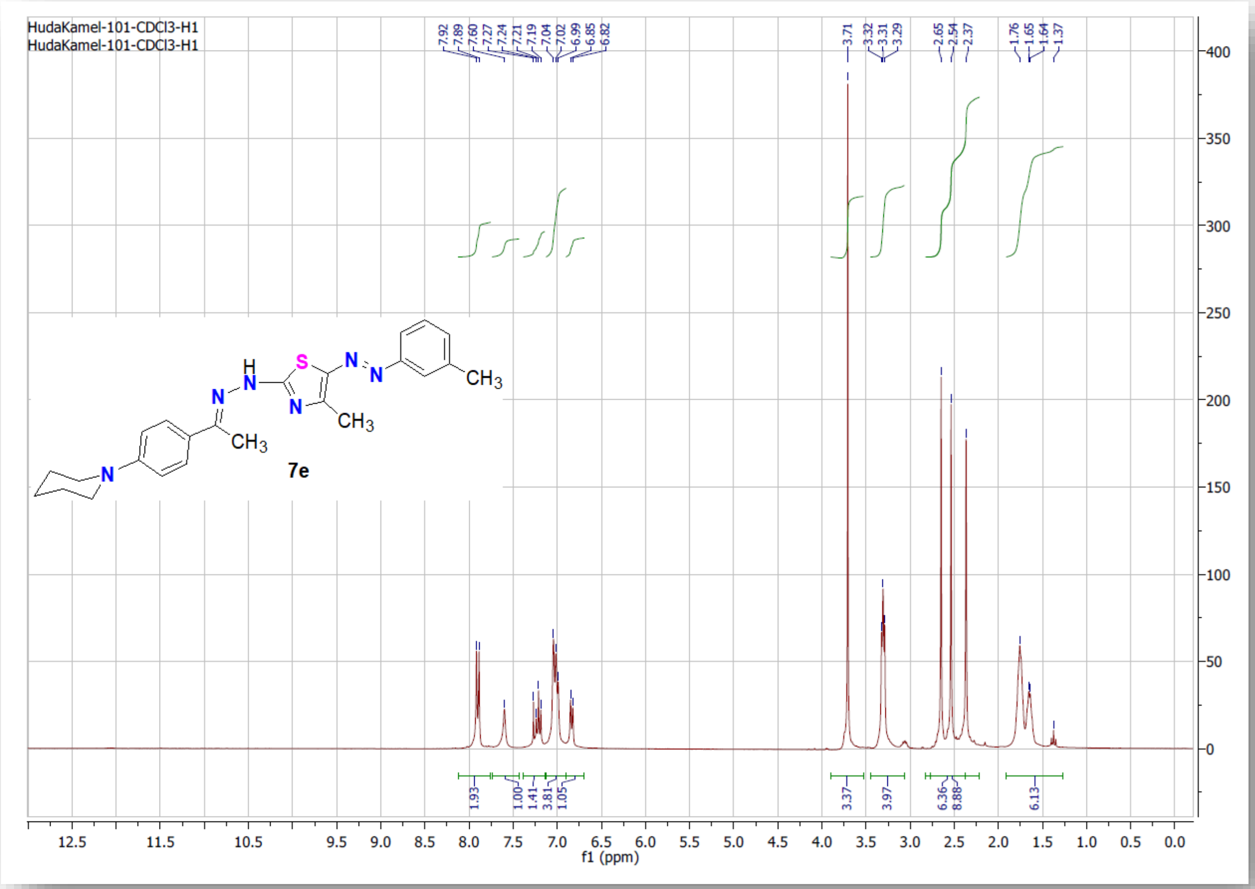

Figure S12. ^{1}H NMR spectrum of compound **7e**

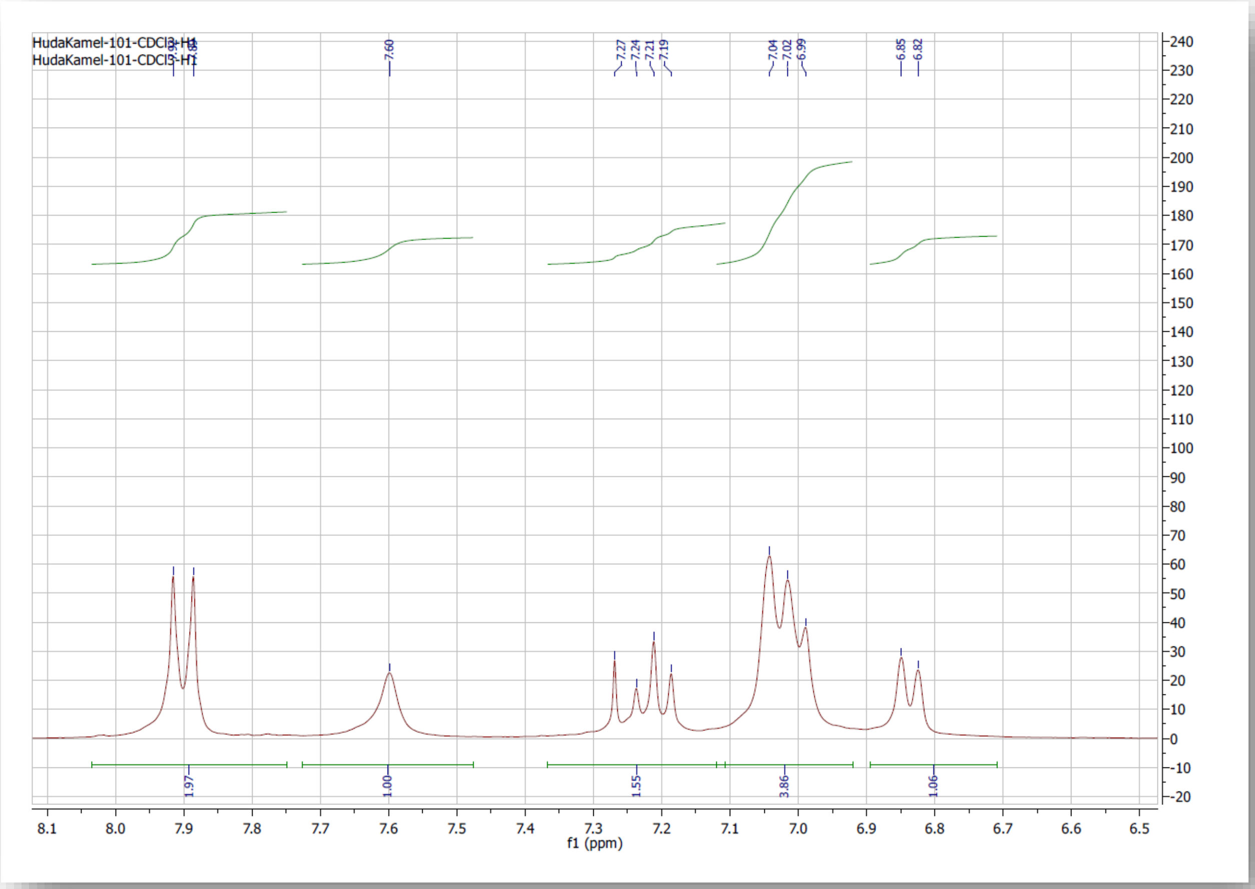

Figure S13. ^{1}H NMR spectrum of compound **7e**

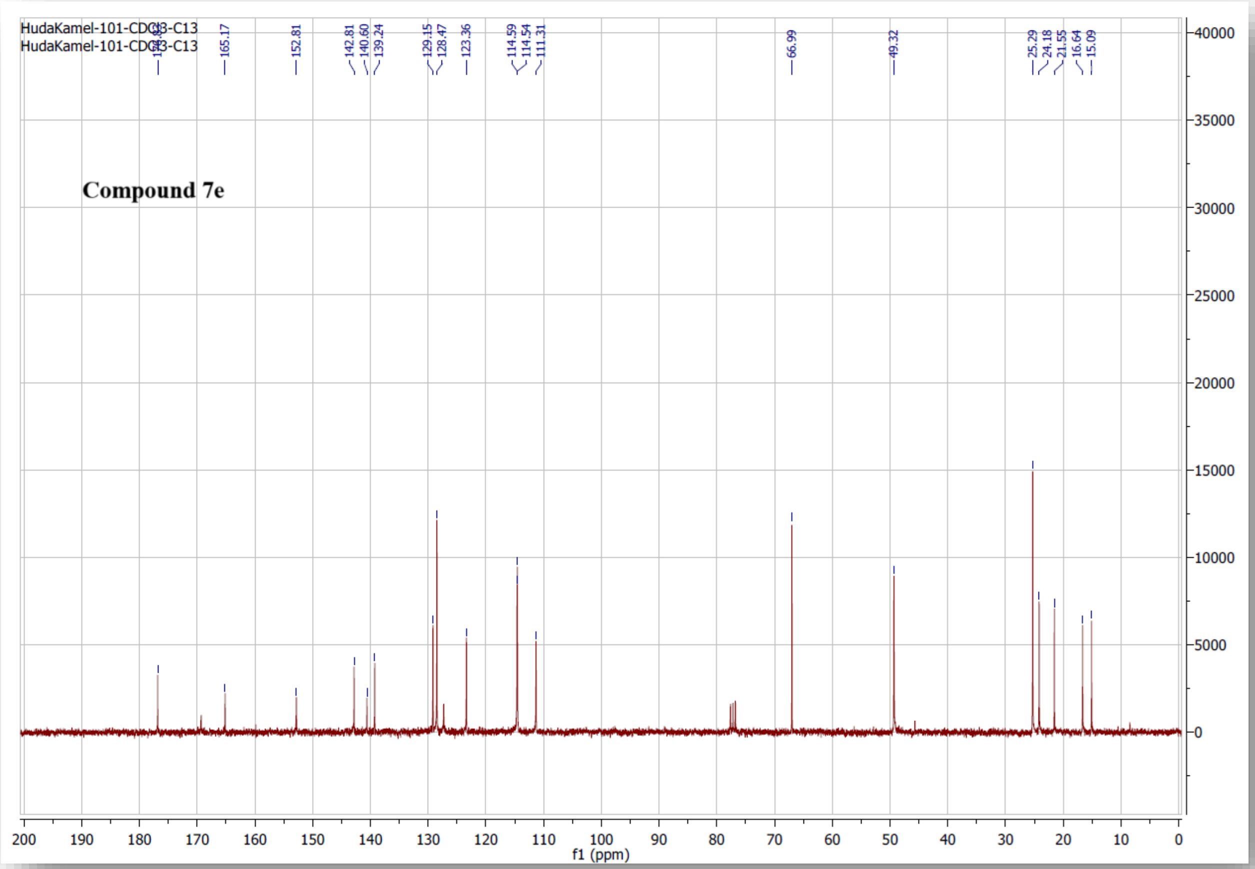

Figure S14. ^{13}C NMR spectrum of compound **7e**

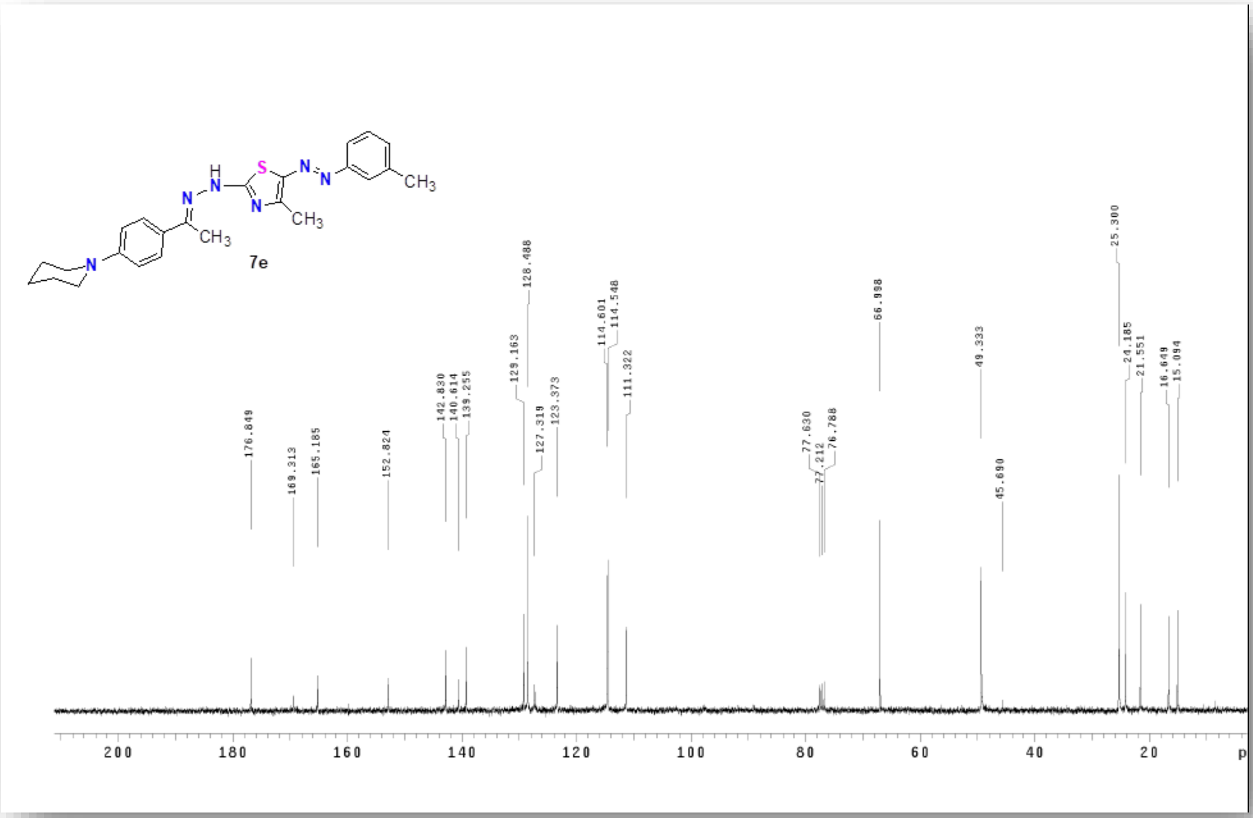

Figure S15. ^{13}C NMR spectrum of compound **7e**

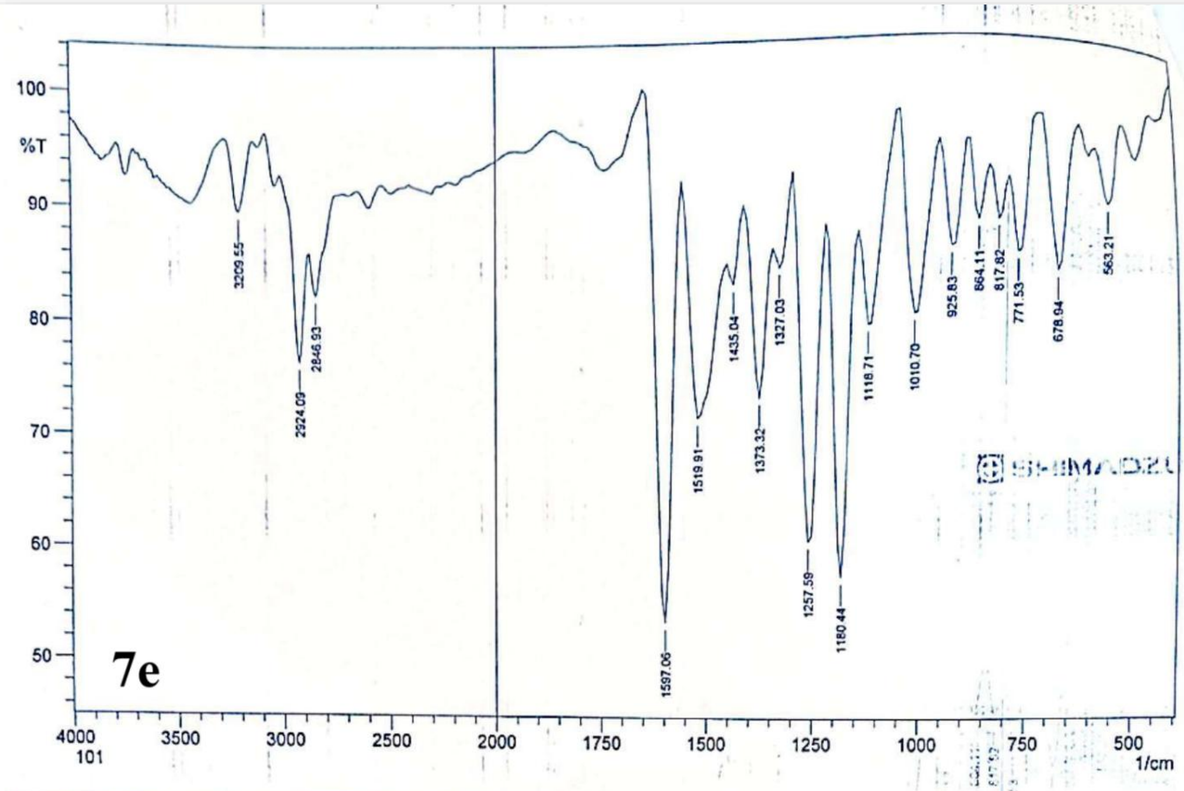

	Peak	Intensity	Corr. Intensity	Base (H)	Base (L)	Area	Corr. Area
1	563.21	89.502	5.981	594.08	524.64	2.277	0.921
2	678.94	83.869	13.064	725.23	632.65	3.768	2.515
3	771.53	85.465	8.267	794.67	725.23	2.854	1.181
4	817.82	88.311	4.102	840.96	794.67	2.002	0.415
5	864.11	88.34	5.721	887.26	840.96	1.917	0.684
6	925.83	86.217	9.041	956.69	887.26	2.941	1.474
7	1010.7	80.67	15.645	1049.28	964.41	5.196	3.683
8	1118.71	79.869	10.573	1141.86	1056.99	4.999	2.354
9	1180.44	57.689	29.092	1211.3	1149.57	9.454	5.641
10	1257.59	60.836	29.774	1296.16	1219.01	10.136	6.738
11	1327.03	84.804	3.762	1342.46	1296.16	2.741	0.529
12	1373.32	73.58	14.105	1404.18	1342.46	5.875	2.301
13	1435.04	83.418	3.742	1450.47	1411.89	2.561	0.347
14	1519.91	71.723	16.505	1550.77	1458.18	10.231	4.653
15	1597.06	53.542	42.222	1643.35	1558.48	11.381	9.756
16	2846.93	82.218	5.028	2877.79	2754.35	7.867	1.427
17	2924.09	76.322	11.615	3008.95	2885.51	9.172	2.89
18	3209.55	89.561	6.305	3271.27	3147.83	4.096	1.828

Figure S16. IR spectrum of compound **7e**

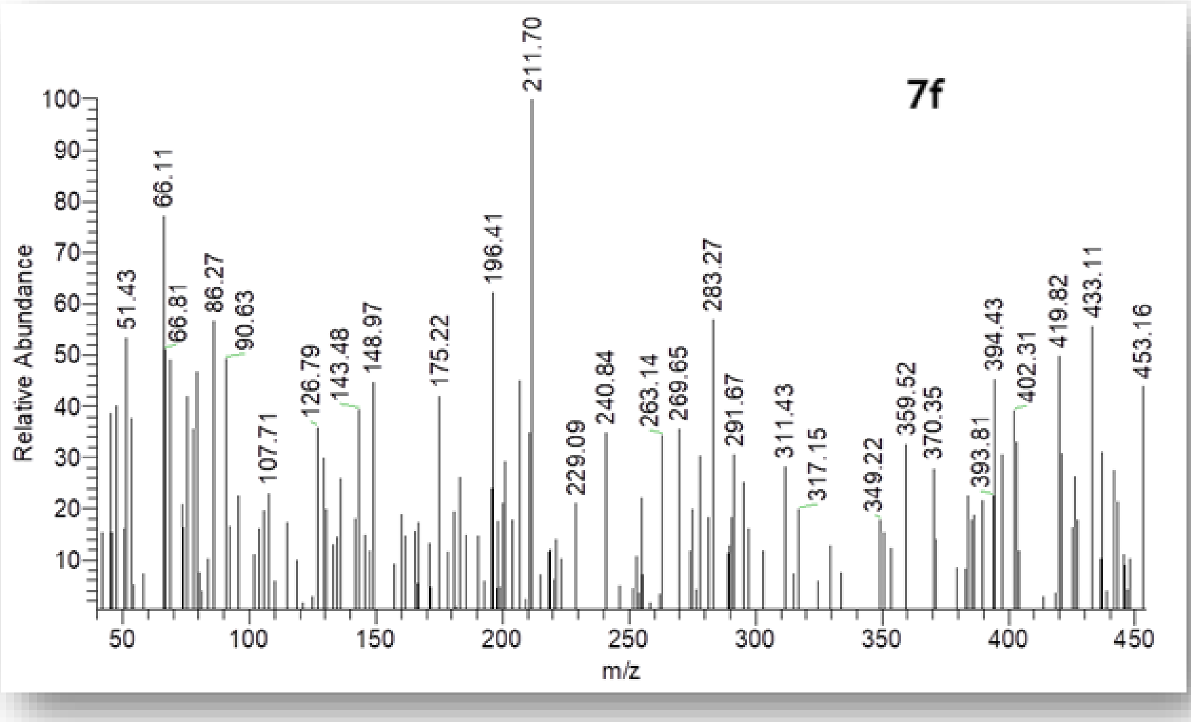

Figure S17. Mass spectrum of compound **7f**

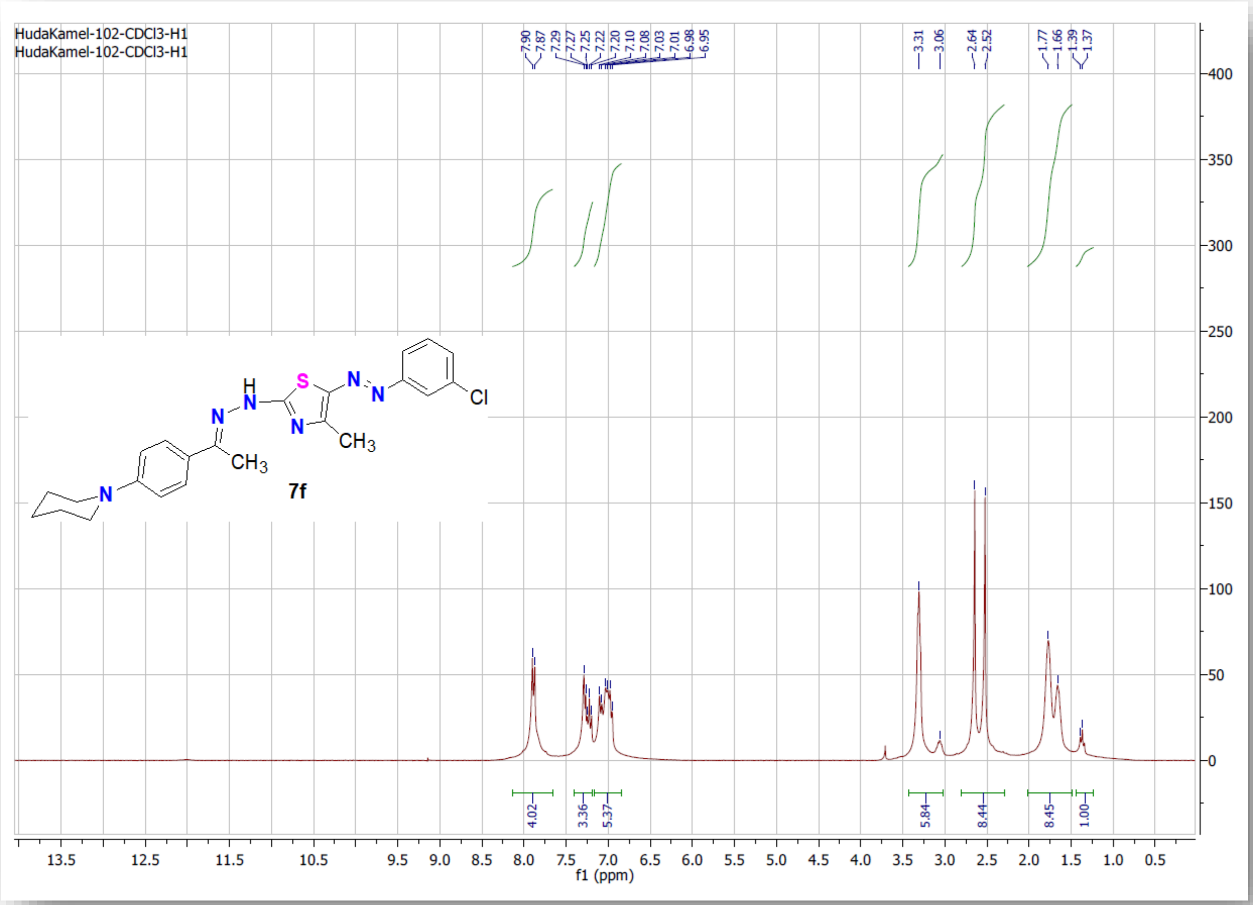

Figure S18. ^{1}H NMR spectrum of compound **7f**

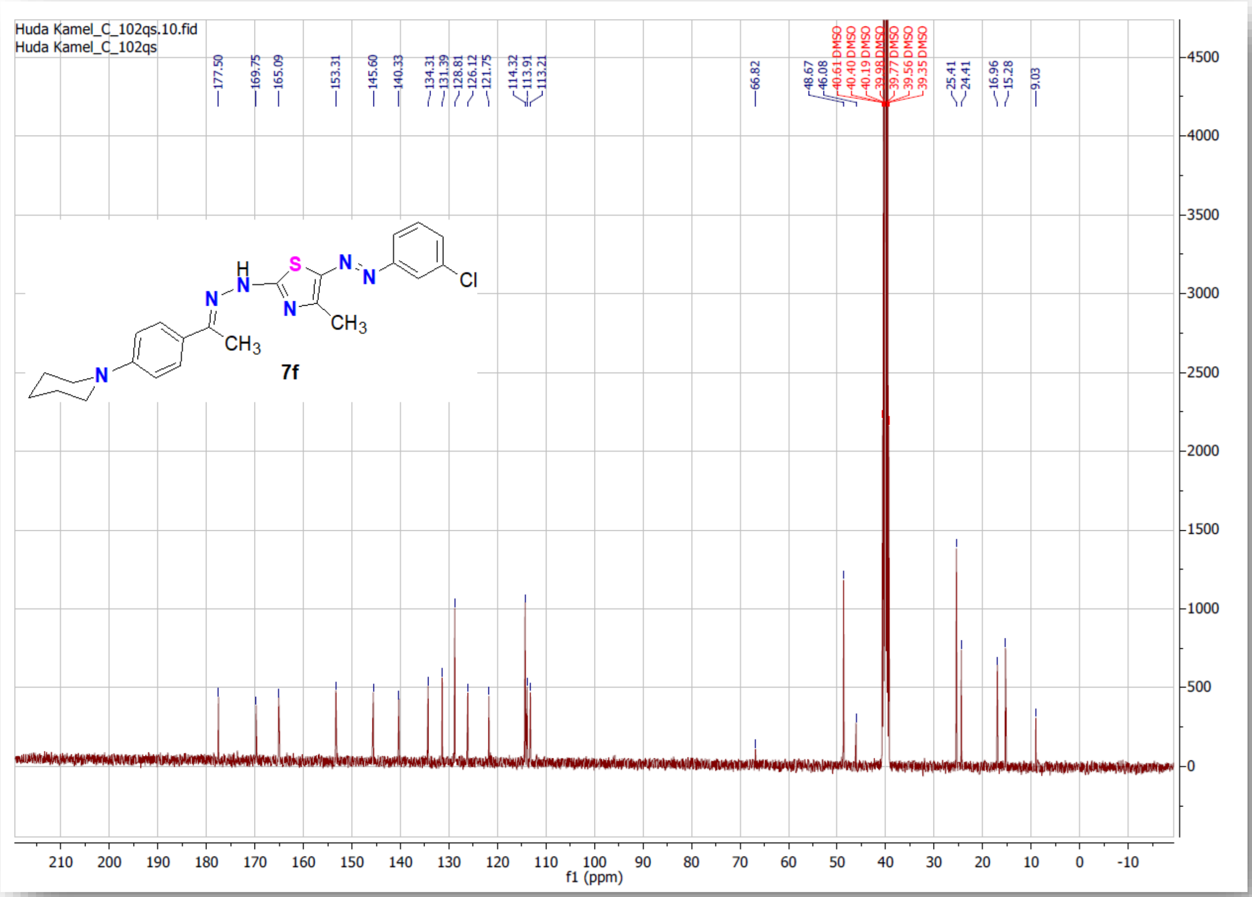

Figure S19. ^{13}C NMR spectrum of compound **7f**

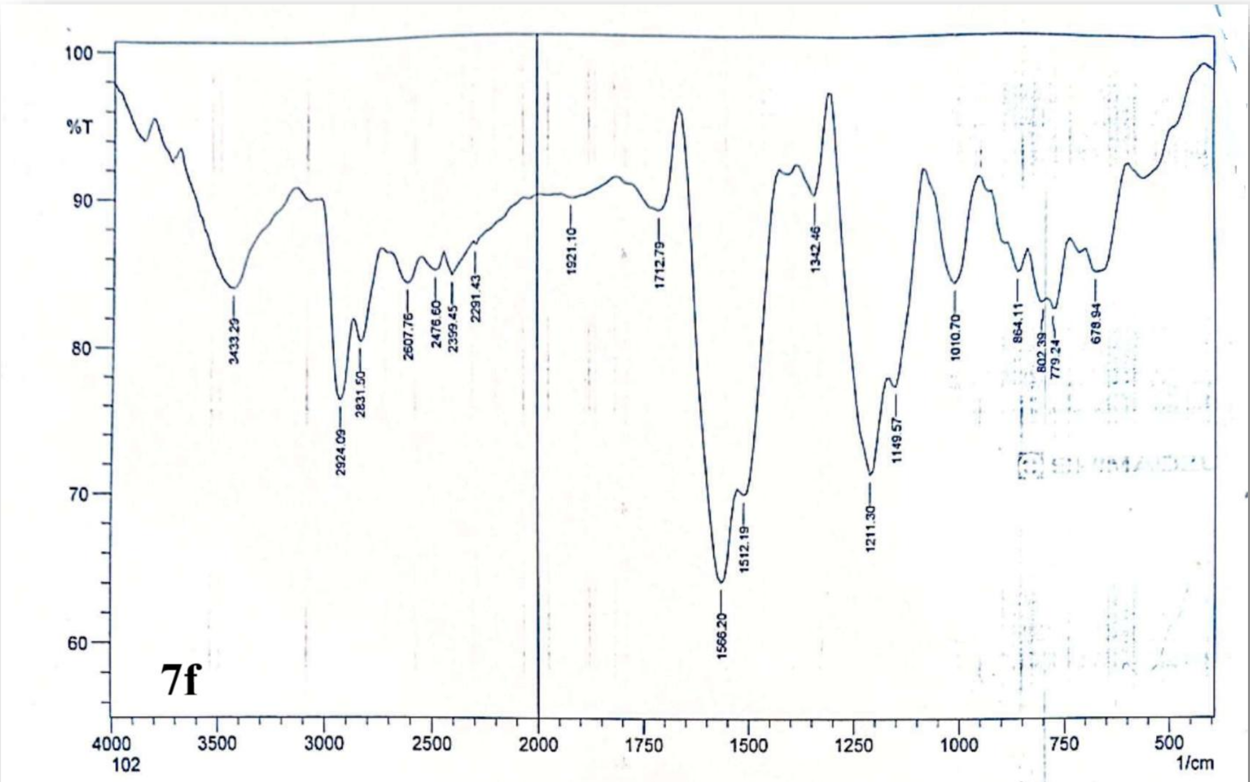

	Peak	Intensity	Corr. Intensity	Base (H)	Base (L)	Area	Corr. Area
1	678.94	84.773	2.965	702.09	609.51	5.64	1.098
2	779.24	82.455	1.814	794.67	740.67	4.07	0.281
3	802.39	82.925	0.771	840.96	794.67	3.481	0.155
4	864.11	84.936	1.673	887.26	840.96	3.11	0.219
5	1010.7	84.267	7.238	1080.14	956.69	7.066	2.314
6	1149.57	77.467	3.329	1165	1087.85	6.174	0.87
7	1211.3	71.498	12.059	1303.88	1172.72	13.602	5.268
8	1342.46	90.134	4.942	1388.75	1311.59	2.798	0.924
9	1512.19	70.044	2.002	1519.91	1427.32	9.008	0.839
10	1566.2	64.212	13.626	1658.78	1527.62	17.899	6.12
11	1712.79	88.921	5.379	1805.37	1666.5	5.967	1.987
12	1921.1	89.694	0.463	1944.25	1813.09	5.831	0.207
13	2291.43	86.519	0.305	2299.15	2067.69	12.723	0.127
14	2399.45	84.508	1.476	2430.31	2306.86	8.437	0.474
15	2476.6	84.811	1.064	2538.32	2438.02	6.968	0.323
16	2607.76	84.02	1.834	2684.91	2546.04	9.894	0.702
17	2831.5	80.219	2.631	2862.36	2731.2	10.727	0.933
18	2924.09	76.312	8.335	3016.67	2870.08	13.514	3.502
19	3433.29	84.018	8.191	3687.9	3140.11	31.192	11.565

Figure S20. IR spectrum of compound **7f**

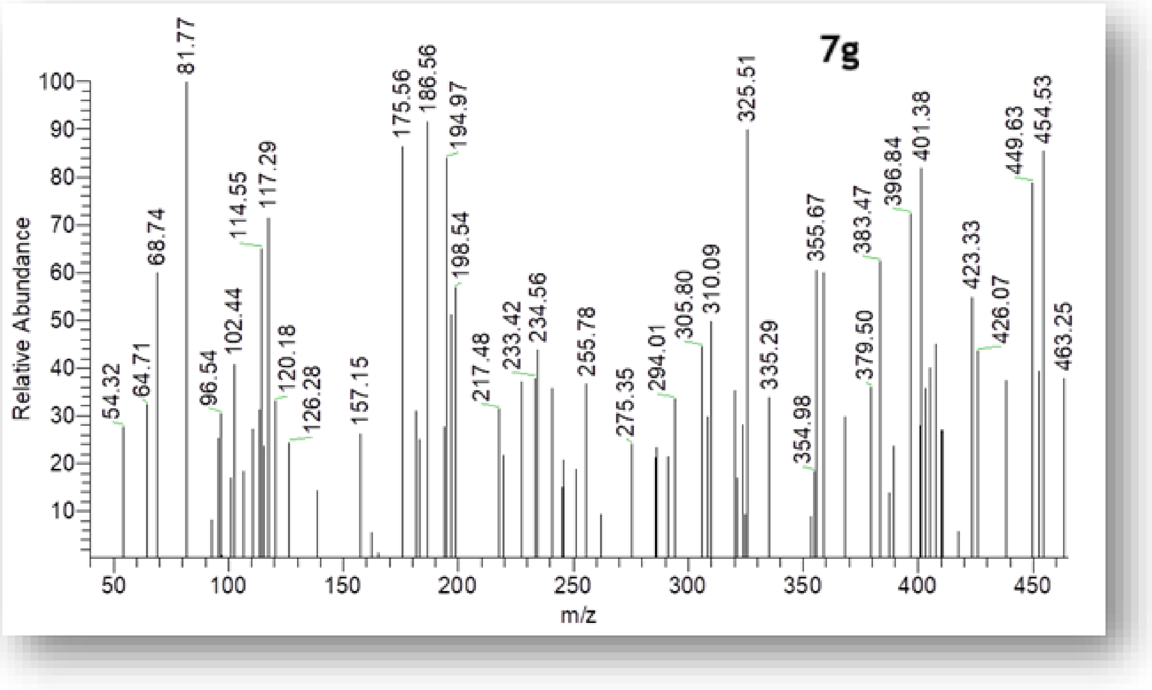

Figure S21. Mass spectrum of compound **7g**

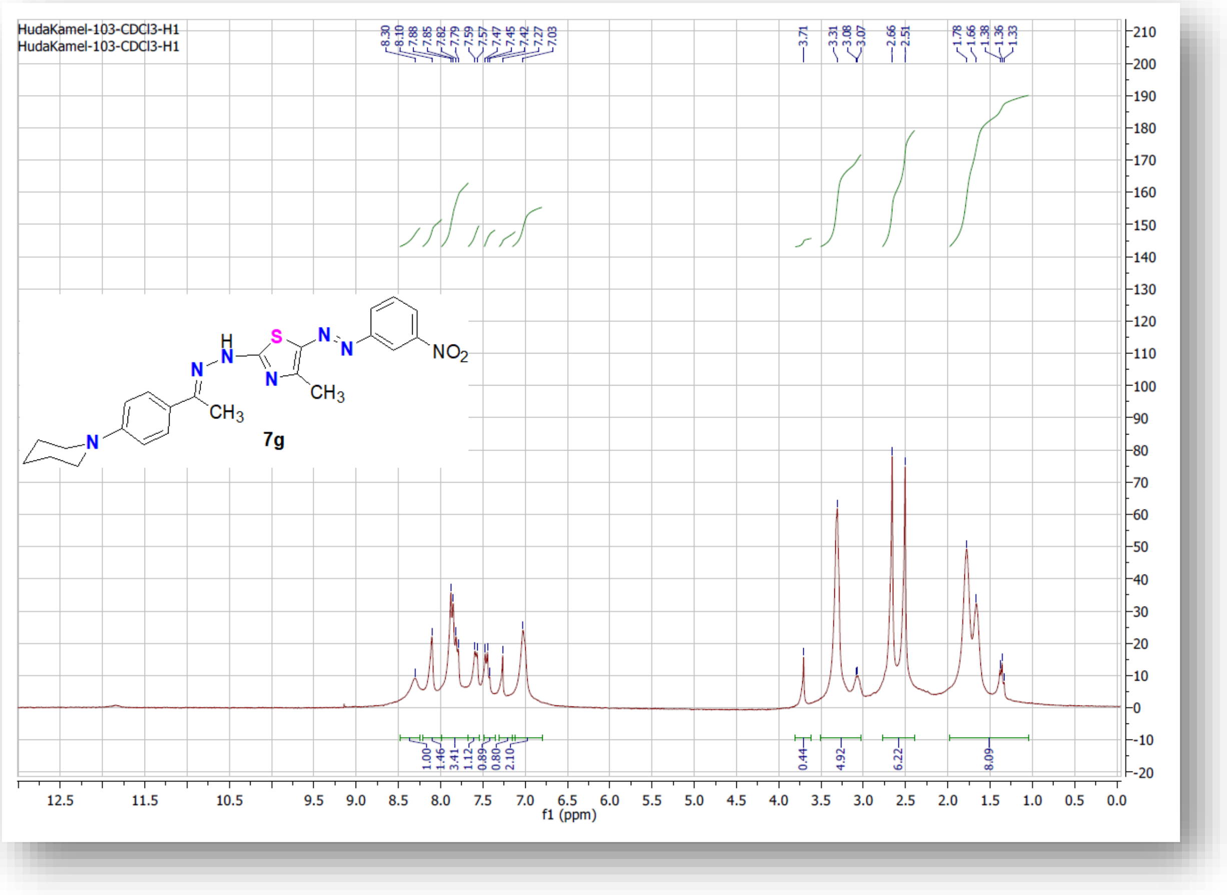

Figure S22. ^{1}H NMR spectrum of compound **7g**

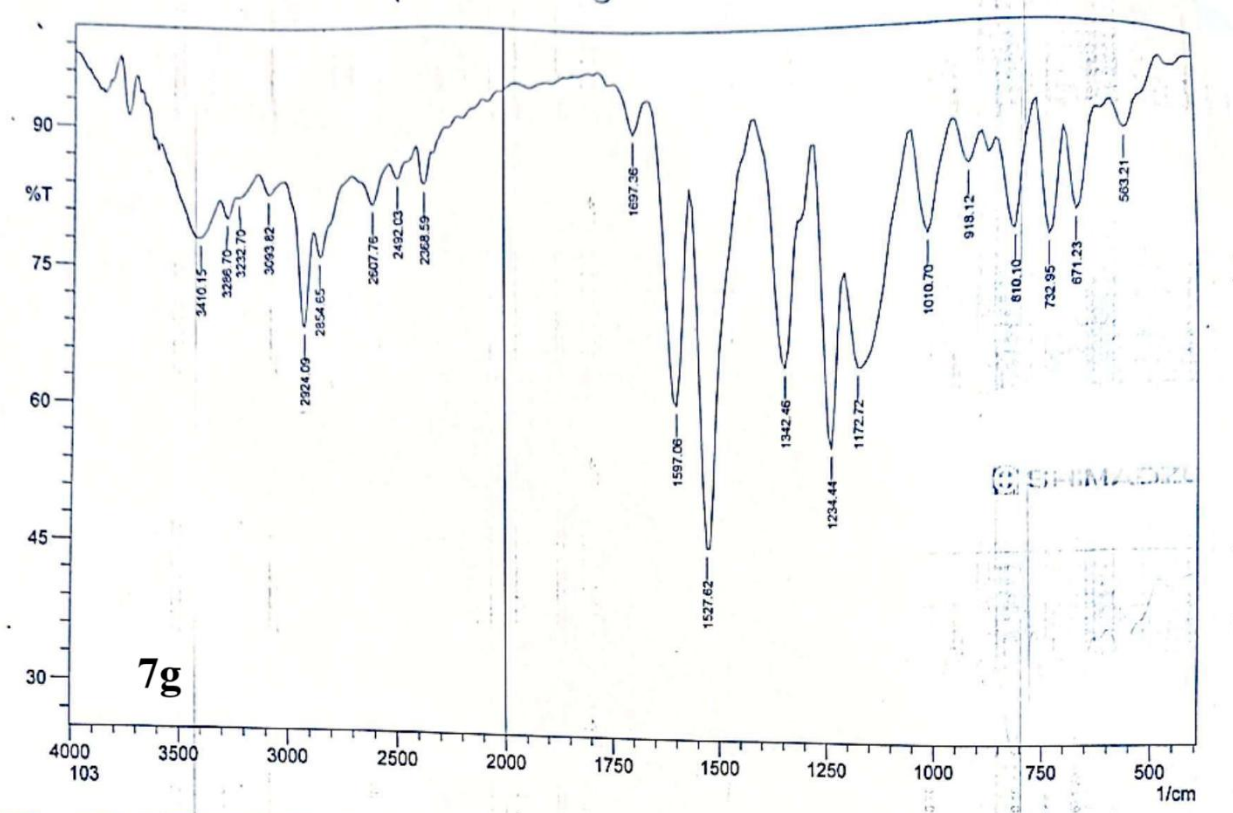

	Peak	Intensity	Corr. Intensity	Base (H)	Base (L)	Area	Corr. Area
1	563.21	89.508	4.384	594.08	478.35	3.698	1.178
2	671.23	81.14	8.608	694.37	632.65	4.223	1.484
3	732.95	78.747	12.377	763.81	702.09	4.767	2.219
4	810.1	79.559	10.702	848.68	771.53	5.469	2.099
5	918.12	86.365	3.739	956.69	887.26	3.722	0.605
6	1010.7	79.493	10.846	1049.28	956.69	6.582	2.55
7	1172.72	65.372	13.082	1203.58	1056.99	19.183	7.062
8	1234.44	56.764	23.052	1273.02	1211.3	10.5	4.653
9	1342.46	65.294	24.858	1411.89	1280.73	13.67	7.758
10	1527.62	45.583	38.787	1558.48	1419.61	21.661	13.318
11	1597.06	60.906	26.37	1658.78	1566.2	11.701	6.706
12	1697.36	89.903	4.411	1743.65	1666.5	2.532	0.631
13	2368.59	83.745	3.66	2399.45	2337.72	4.311	0.696
14	2492.03	84.306	2.226	2530.61	2407.16	8.206	0.705
15	2607.76	81.556	3.765	2700.34	2538.32	12.551	1.333
16	2854.65	75.949	3.392	2877.79	2708.06	15.823	1.038
17	2924.09	68.358	11.897	3016.67	2885.51	15.307	3.437
18	3093.82	82.764	1.893	3140.11	3024.38	8.983	0.546
19	3232.7	82.448	0.213	3240.41	3147.83	7.254	0.149
20	3286.7	80.237	2.061	3317.56	3248.13	6.267	0.405
21	3410.15	78.1	5.916	3595.31	3325.28	24.153	5.089

Figure S23. IR spectrum of compound **7g**

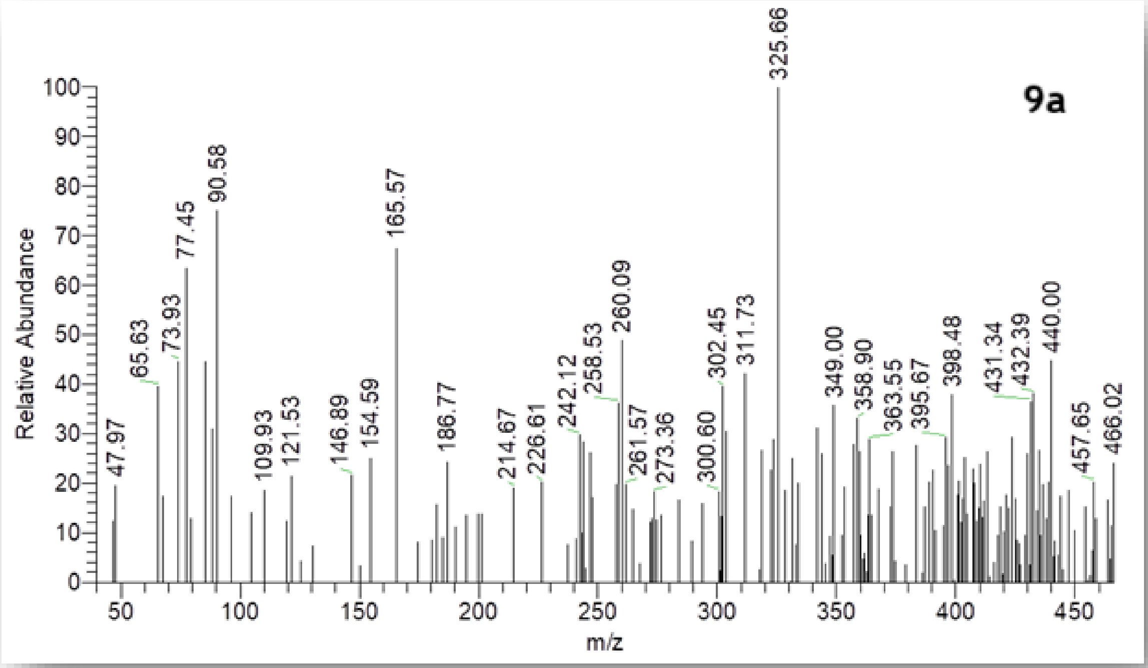

Figure S24. Mass spectrum of compound **9a**

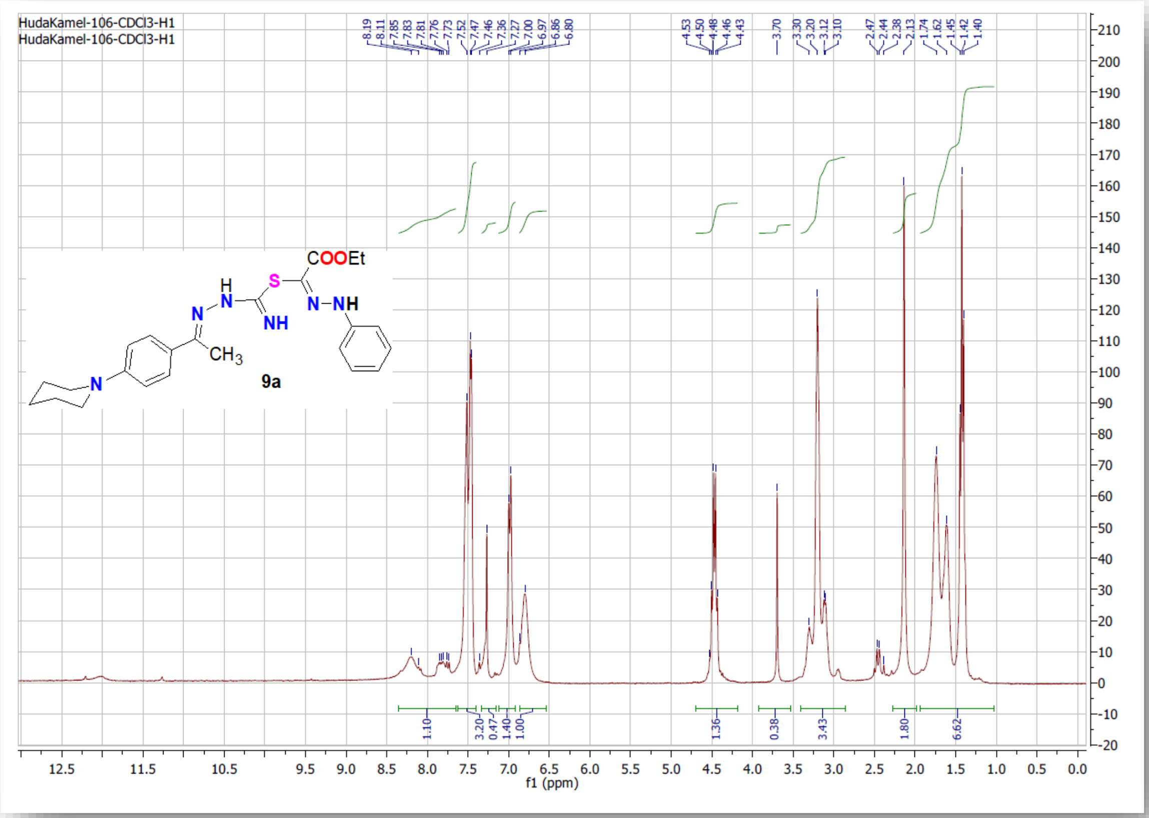

Figure S25. ^{1}H NMR spectrum of compound **9a**

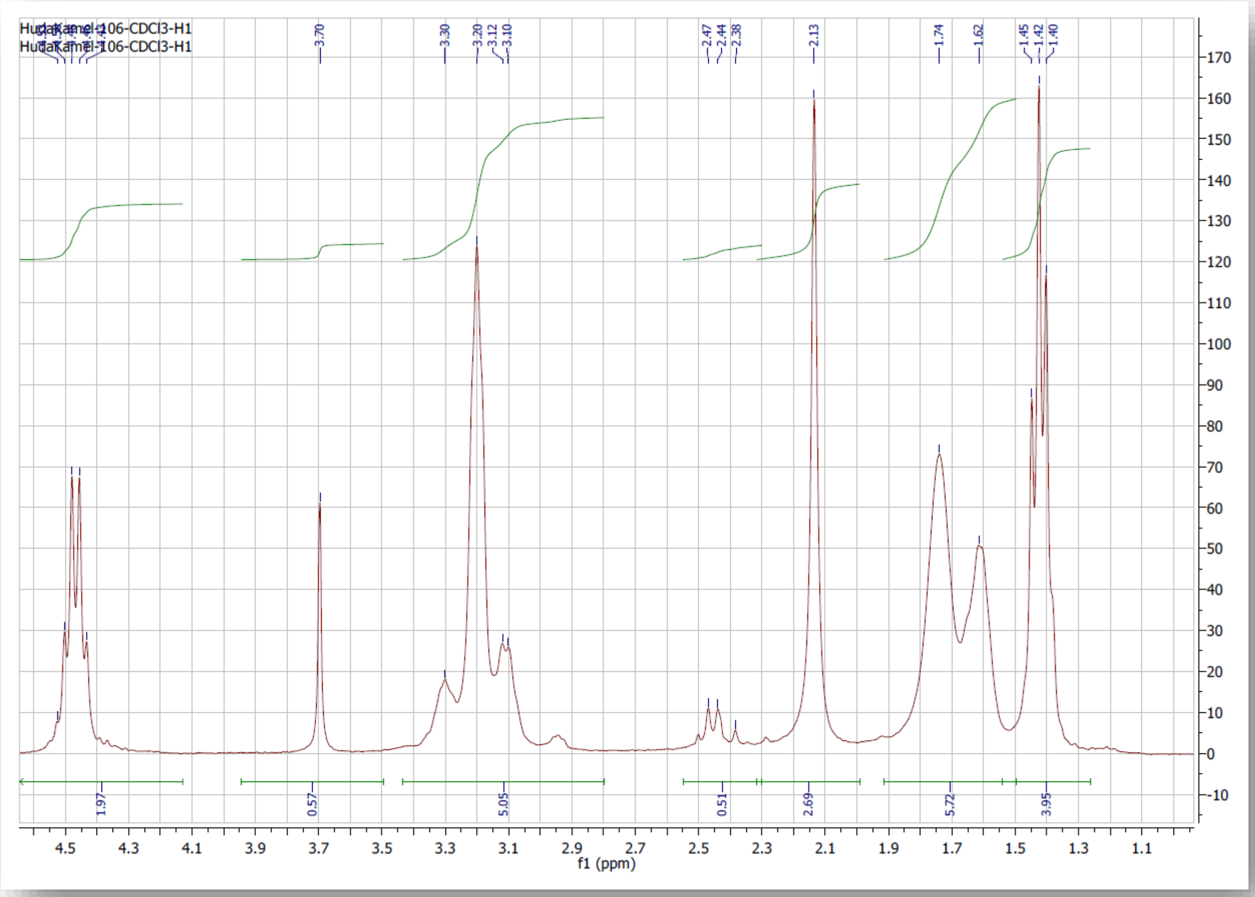

Figure S26. ^{1}H NMR spectrum of compound **9a**

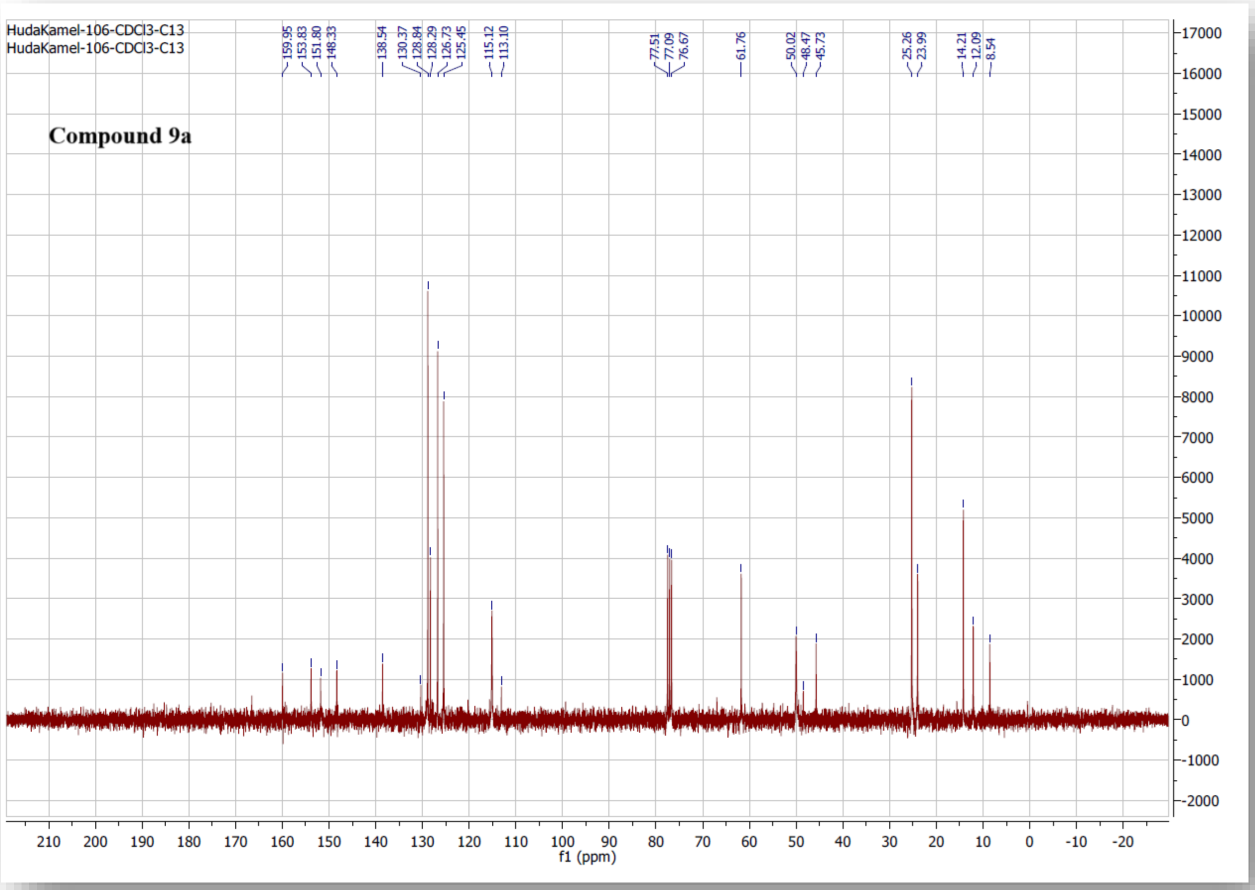

Figure S27. ^{13}C NMR spectrum of compound **9a**

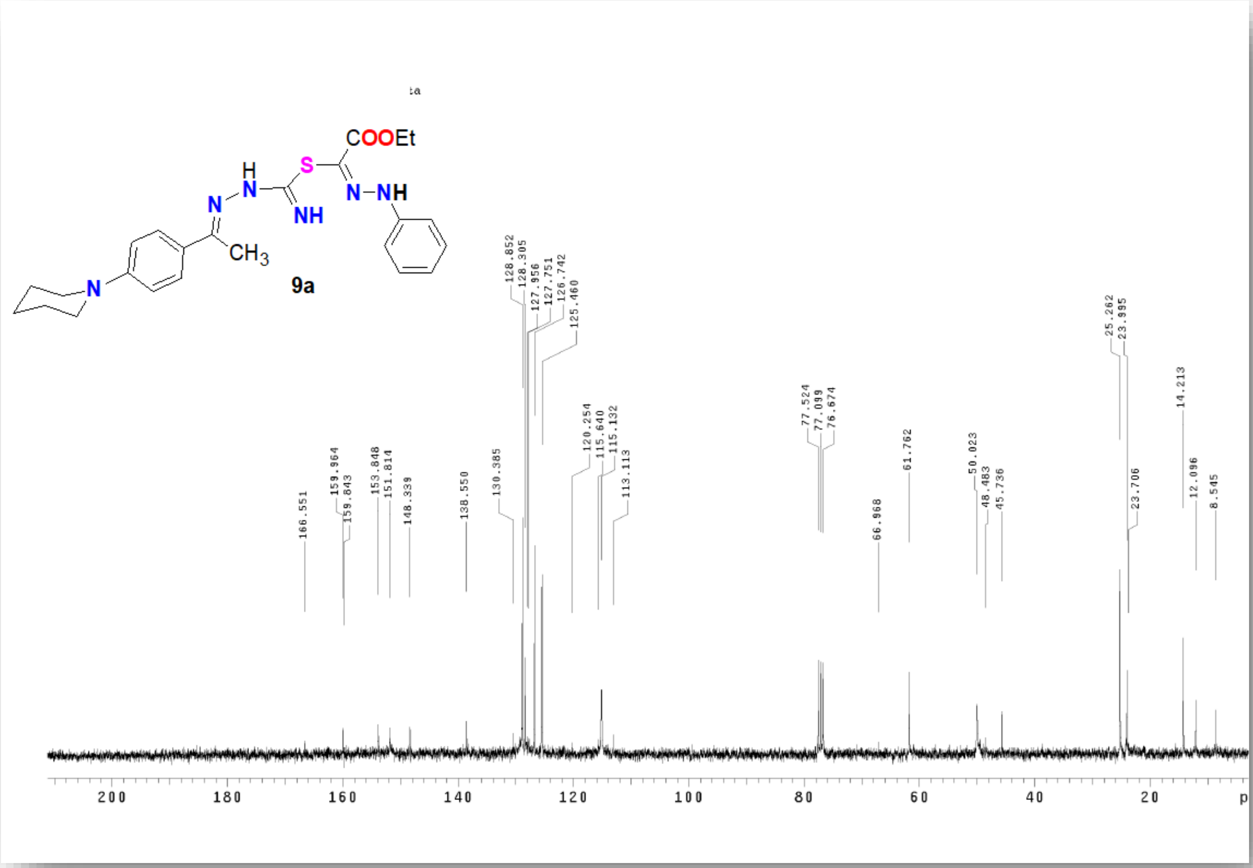

Figure S28. ^{1}H NMR spectrum of compound **9a**

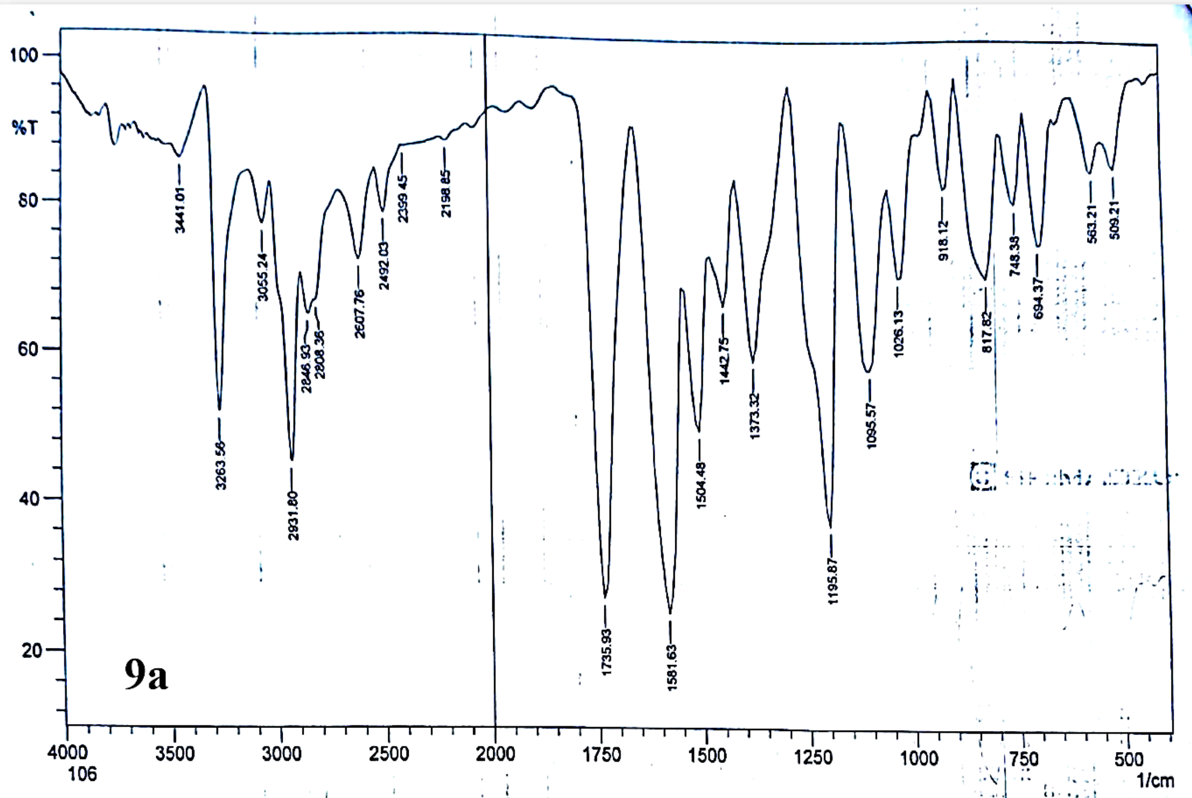

	Peak	Intensity	Corr. Intensity	Base (H)	Base (L)	Area	Corr. Area
1	509.21	86.215	6.765	540.07	447.49	3.068	0.741
2	563.21	85.602	6.171	617.22	540.07	3.381	0.922
3	694.37	75.617	15.073	717.52	663.51	4.91	2.669
4	748.38	81.262	11.4	786.96	725.23	3.959	1.811
5	817.82	71.226	21.04	879.54	786.96	9.899	6.875
6	918.12	83.514	14.19	948.98	887.26	2.854	2.231
7	1026.13	71.532	14.74	1049.28	987.55	6.263	2.705
8	1095.57	59.064	27.268	1149.57	1056.99	14.273	8.494
9	1195.87	37.728	55.75	1273.02	1157.29	24.889	21.658
10	1373.32	60.069	28.283	1411.89	1280.73	15.419	10.145
11	1442.75	67.439	11.617	1473.62	1419.61	7.53	1.983
12	1504.48	50.381	21.392	1535.34	1481.33	12.426	4.636
13	1581.63	25.6	51.904	1651.07	1543.05	36.297	25.304
14	1735.93	27.599	66.296	1836.23	1658.78	30.447	25.748
15	2198.85	88.969	0.799	2222	2106.27	5.27	0.195
16	2399.45	88.119	0.159	2407.16	2229.71	9.305	0.178
17	2492.03	78.94	6.996	2530.61	2407.16	9.526	1.843
18	2607.76	72.434	10.978	2700.34	2538.32	17.045	4.161
19	2808.36	66.842	1.065	2816.07	2708.06	12.679	0.09
20	2846.93	65.055	3.233	2877.79	2823.79	9.601	0.646
21	2931.8	45.434	28.775	3016.67	2885.51	28.038	12.018
22	3055.24	77.114	6.239	3116.97	3024.38	8.76	1.536
23	3263.56	51.981	40.741	3317.56	3124.68	24.79	15.768
24	3441.01	86.101	4.38	3495.01	3325.28	7.91	1.805

Figure S29. IR spectrum of compound **9a**

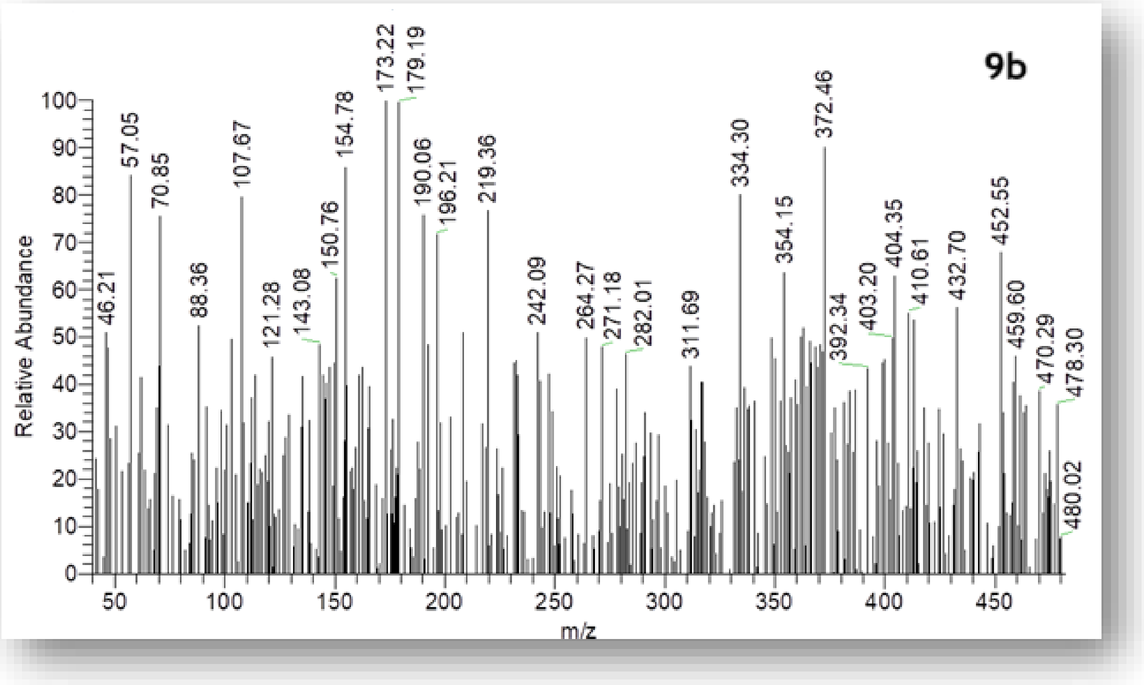

Figure S30. Mass spectrum of compound **9b**

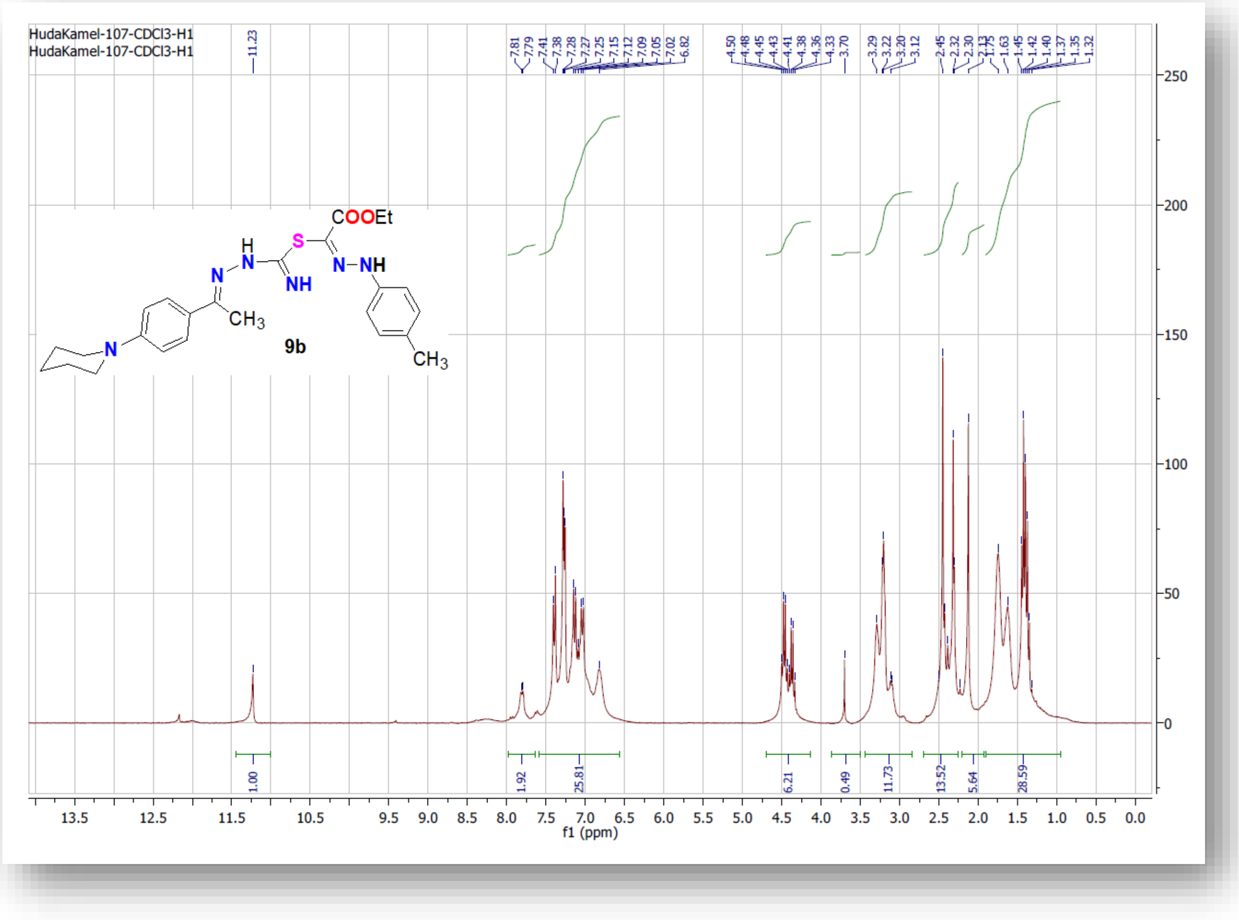

Figure S31. ^{1}H NMR spectrum of compound **9b**

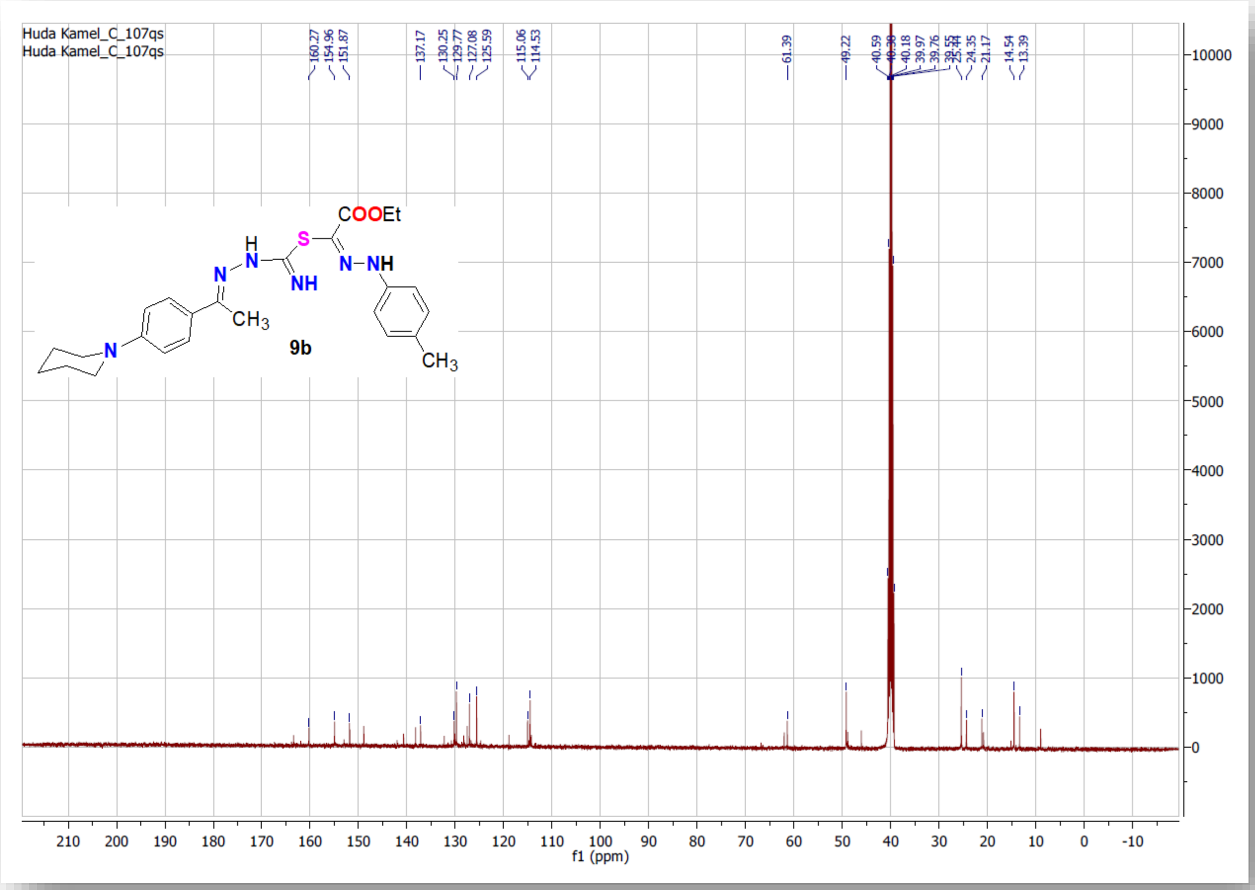

Figure S32. ^{13}C NMR spectrum of compound **9b**

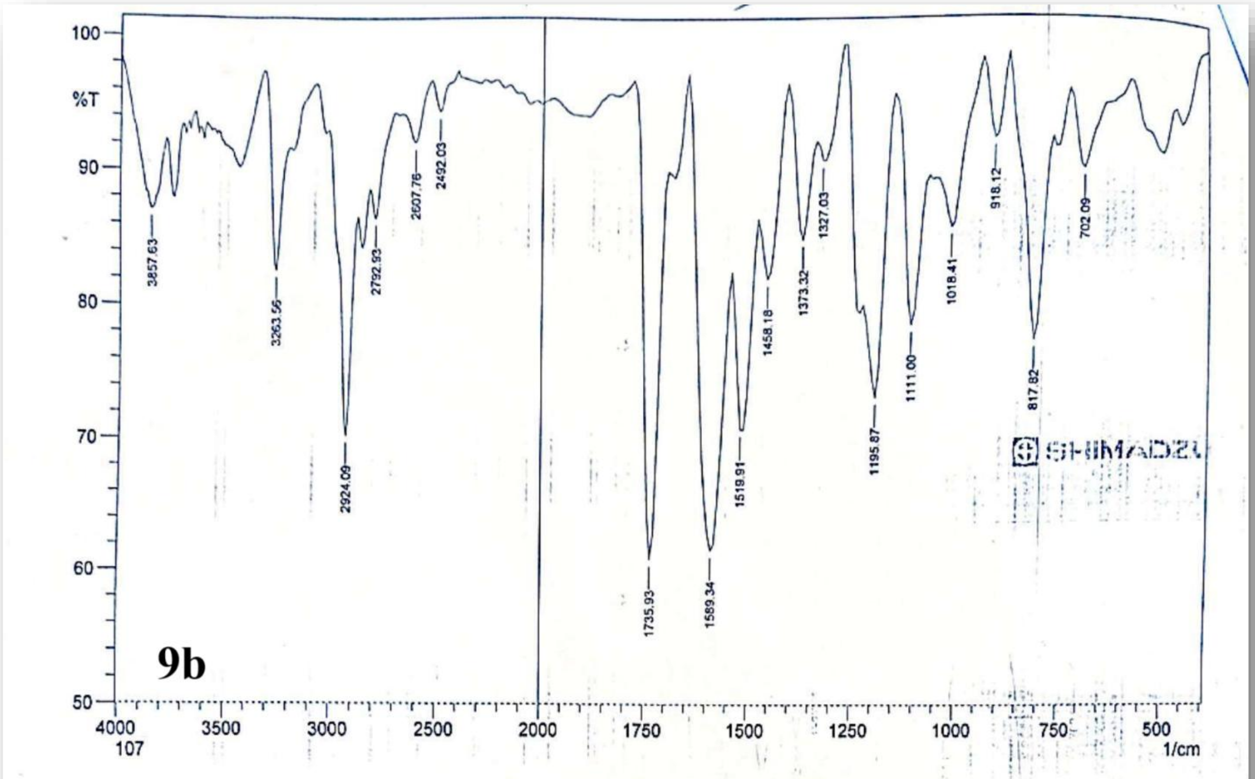

	Peak	Intensity	Corr. Intensity	Base (H)	Base (L)	Area	Corr. Area
1	702.09	90.188	6.01	740.67	594.08	4.011	1.665
2	817.82	77.507	16.143	887.26	786.96	6.147	3.777
3	918.12	92.473	6.05	948.98	887.26	1.308	0.909
4	1018.41	85.982	6.26	1049.28	948.98	3.923	1.309
5	1111	78.768	13.194	1149.57	1080.14	4.853	2.31
6	1195.87	73.294	13.817	1226.73	1157.29	6.093	2.419
7	1327.03	90.996	3.169	1342.46	1280.73	1.817	0.69
8	1373.32	85.091	9.161	1411.89	1342.46	3.204	1.486
9	1458.18	82.031	7.835	1481.33	1411.89	4.174	1.464
10	1519.91	70.685	13.296	1543.05	1481.33	7.033	2.586
11	1589.34	61.582	25.233	1651.07	1550.77	14.166	8.458
12	1735.93	60.865	32.204	1774.51	1697.36	9.29	6.757
13	2492.03	94.588	2.486	2530.61	2407.16	2.122	0.603
14	2607.76	92.245	3.221	2669.48	2530.61	3.573	0.857
15	2792.93	86.509	3.274	2816.07	2708.06	4.61	0.525
16	2924.09	70.075	18.023	3016.67	2885.51	12.167	5.709
17	3263.56	82.618	12.166	3317.56	3201.83	5.917	3.002
18	3857.63	87.06	7.141	4004.22	3788.19	8.479	3.79

Figure S33. IR spectrum of compound **9b**

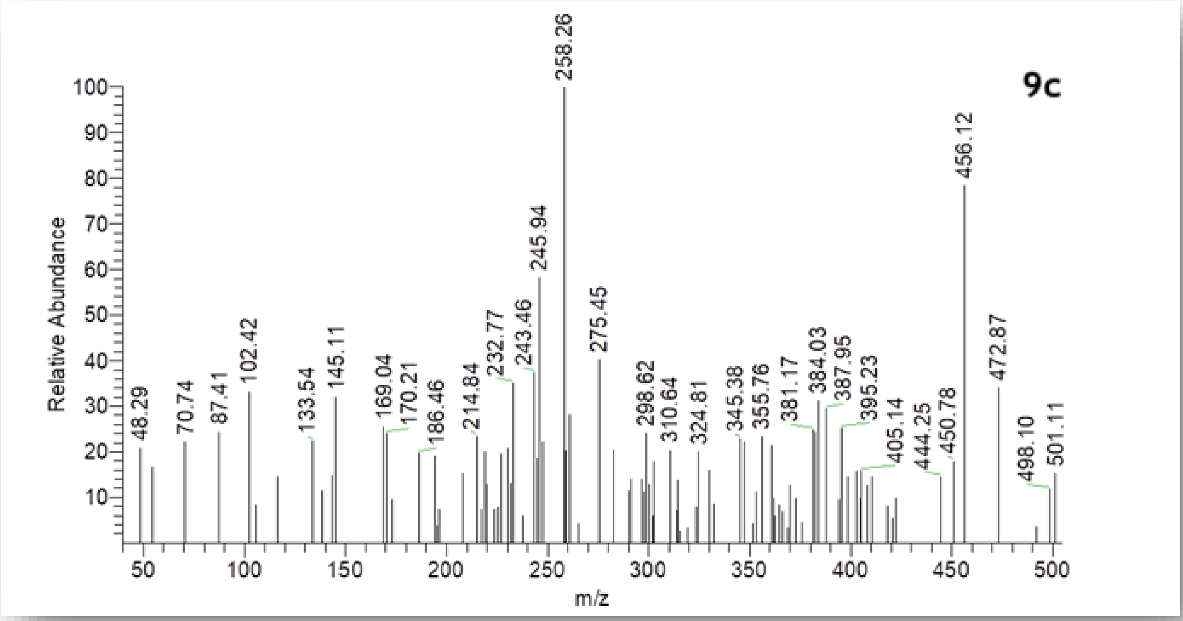

Figure S34. Mass spectrum of compound **9c**

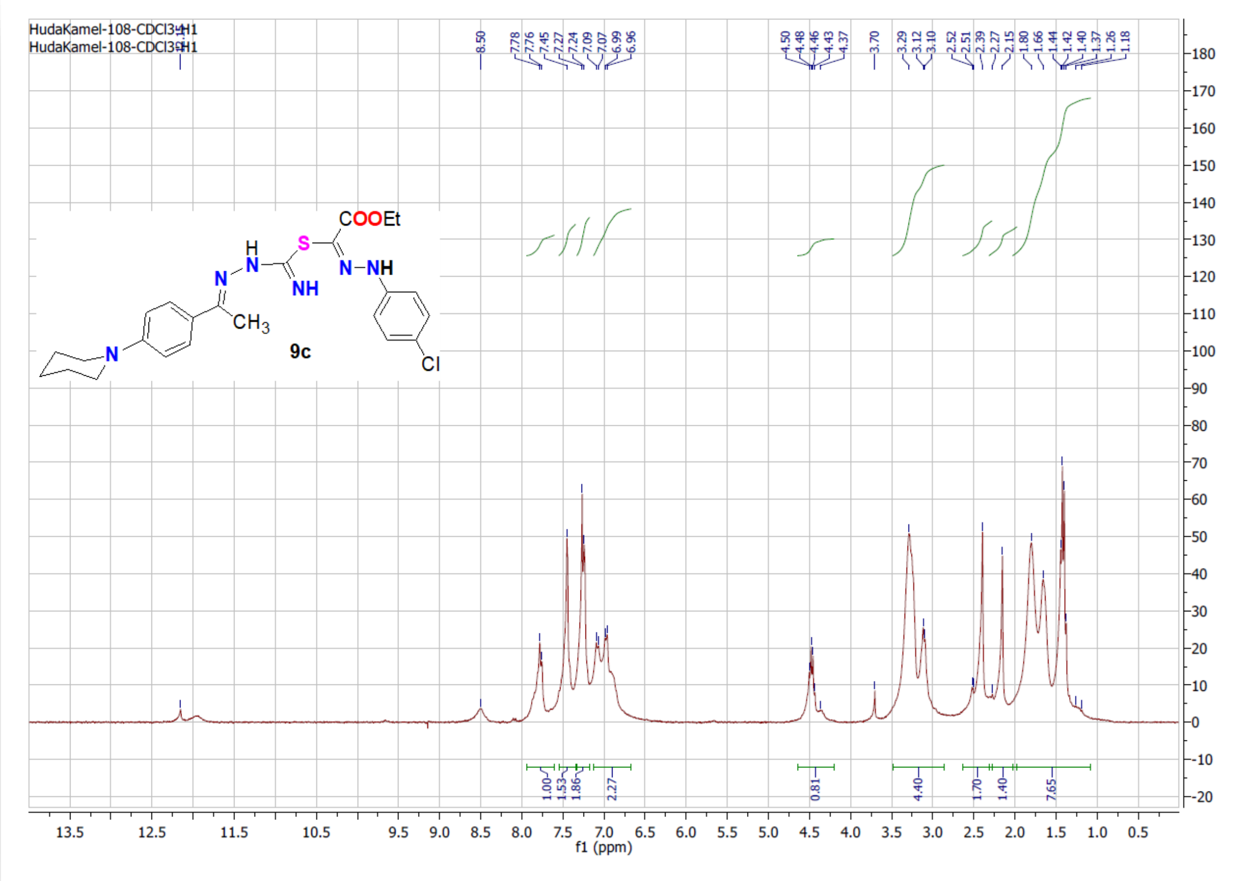

Figure S35. ^{1}H NMR spectrum of compound **9c**

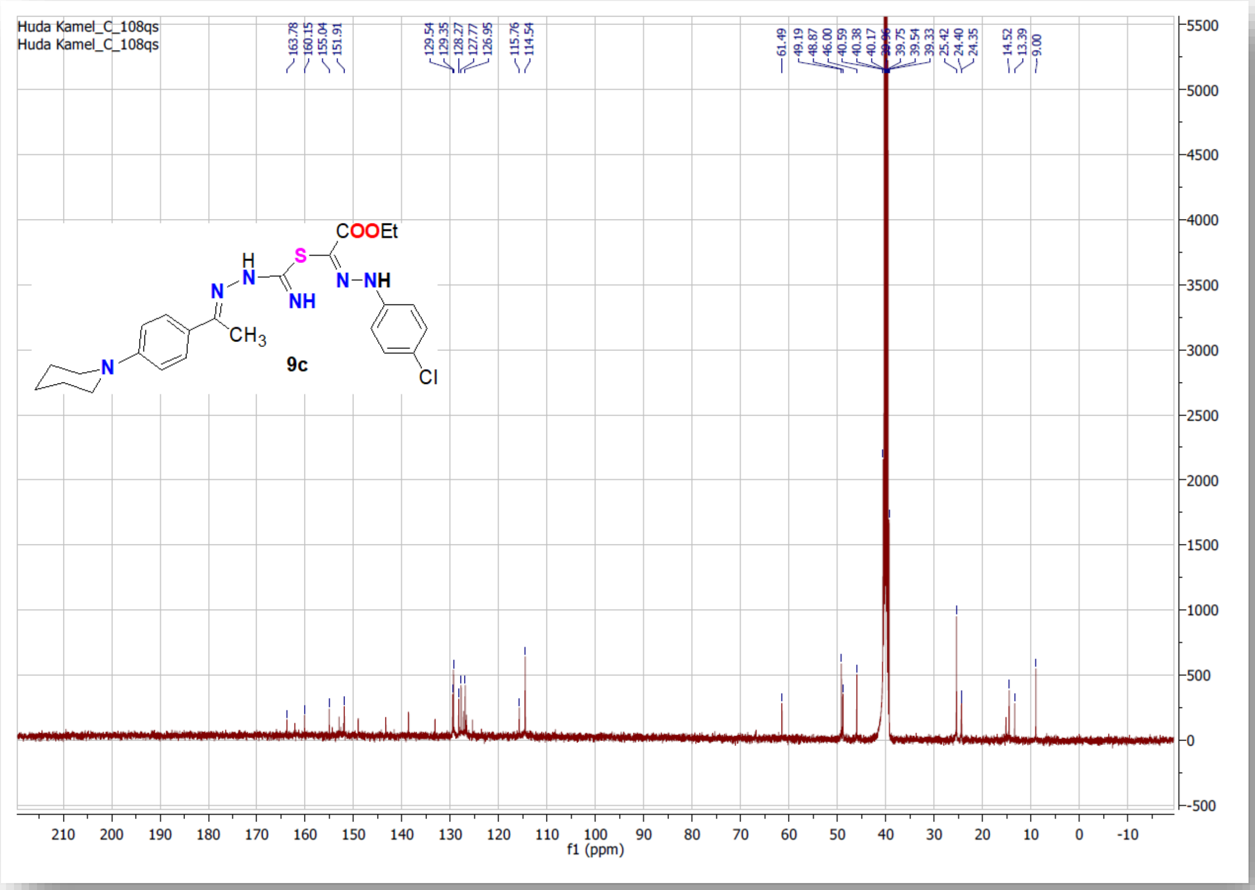

Figure S36. ^{13}C NMR spectrum of compound **9c**

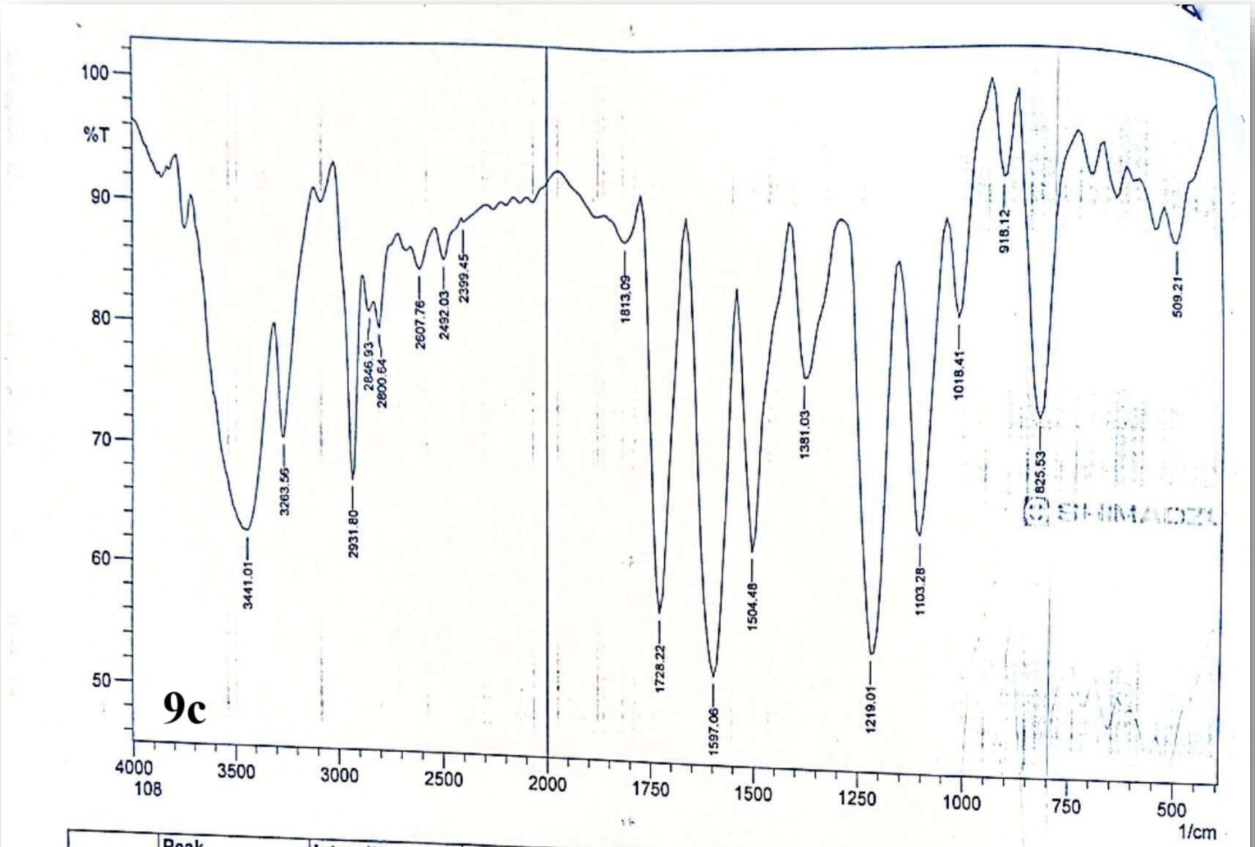

	Peak	Intensity	Corr. Intensity	Base (H)	Base (L)	Area	Corr. Area
1	509.21	88.274	4.752	540.07	393.48	3.889	1.24
2	825.53	74.018	24.265	887.26	748.38	8.668	7.46
3	918.12	93.02	7.062	948.98	887.26	1.016	1.037
4	1018.41	82.012	11.116	1049.28	948.98	4.275	2.056
5	1103.28	64.512	23.656	1157.29	1049.28	12.861	7.01
6	1219.01	54.79	32.933	1288.45	1165	19.199	12.234
7	1381.03	77.136	12.22	1411.89	1296.16	9.37	3.814
8	1504.48	62.793	23.011	1543.05	1419.61	14.579	7.091
9	1597.06	52.382	32.383	1666.5	1550.77	21.334	13.291
10	1728.22	57.364	32.417	1774.51	1674.21	14.483	9.646
11	1813.09	87.467	2.674	1859.38	1782.23	4.045	0.551
12	2399.45	88.538	0.425	2407.16	2299.15	5.325	0.111
13	2492.03	85.445	2.753	2530.61	2414.88	6.938	0.697
14	2607.76	84.593	2.348	2646.34	2538.32	7.022	0.61
15	2800.64	79.76	3.22	2823.79	2715.77	8.045	0.47
16	2846.93	81.124	1.411	2877.79	2831.5	4.066	0.248
17	2931.8	67.239	19.926	3016.67	2885.51	13.55	6.344
18	3263.56	70.598	12.176	3309.85	3124.68	17.494	5.02
19	3441.01	62.713	20.327	3711.04	3317.56	57.875	29.503

Figure S37. IR spectrum of compound **9c**

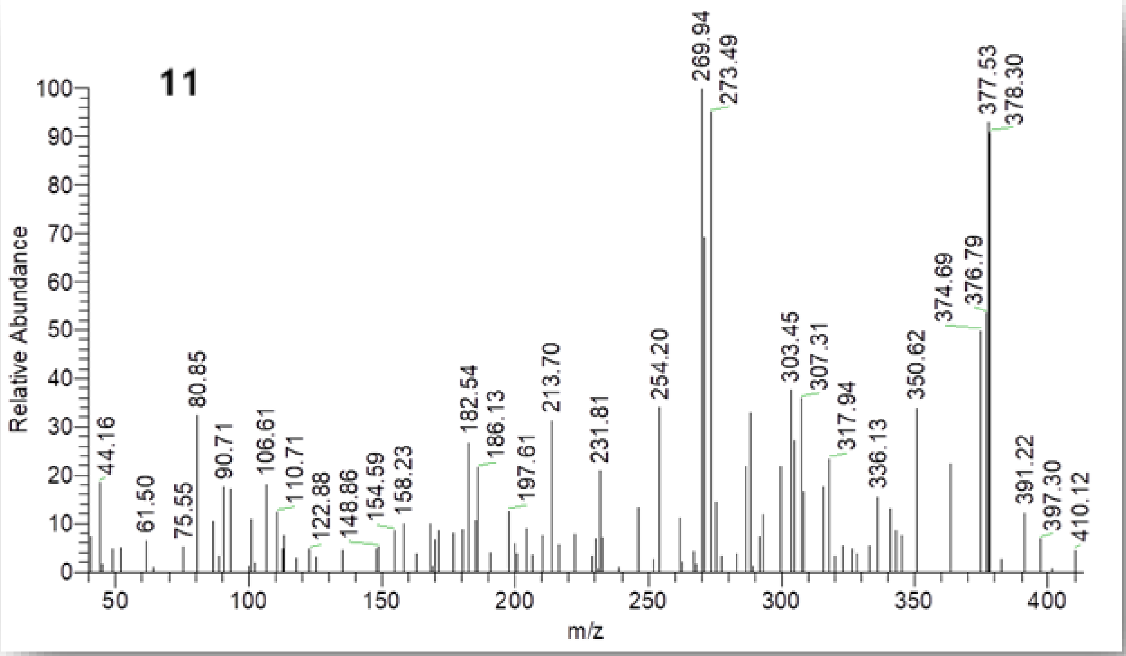

Figure S38. Mass spectrum of compound **11**

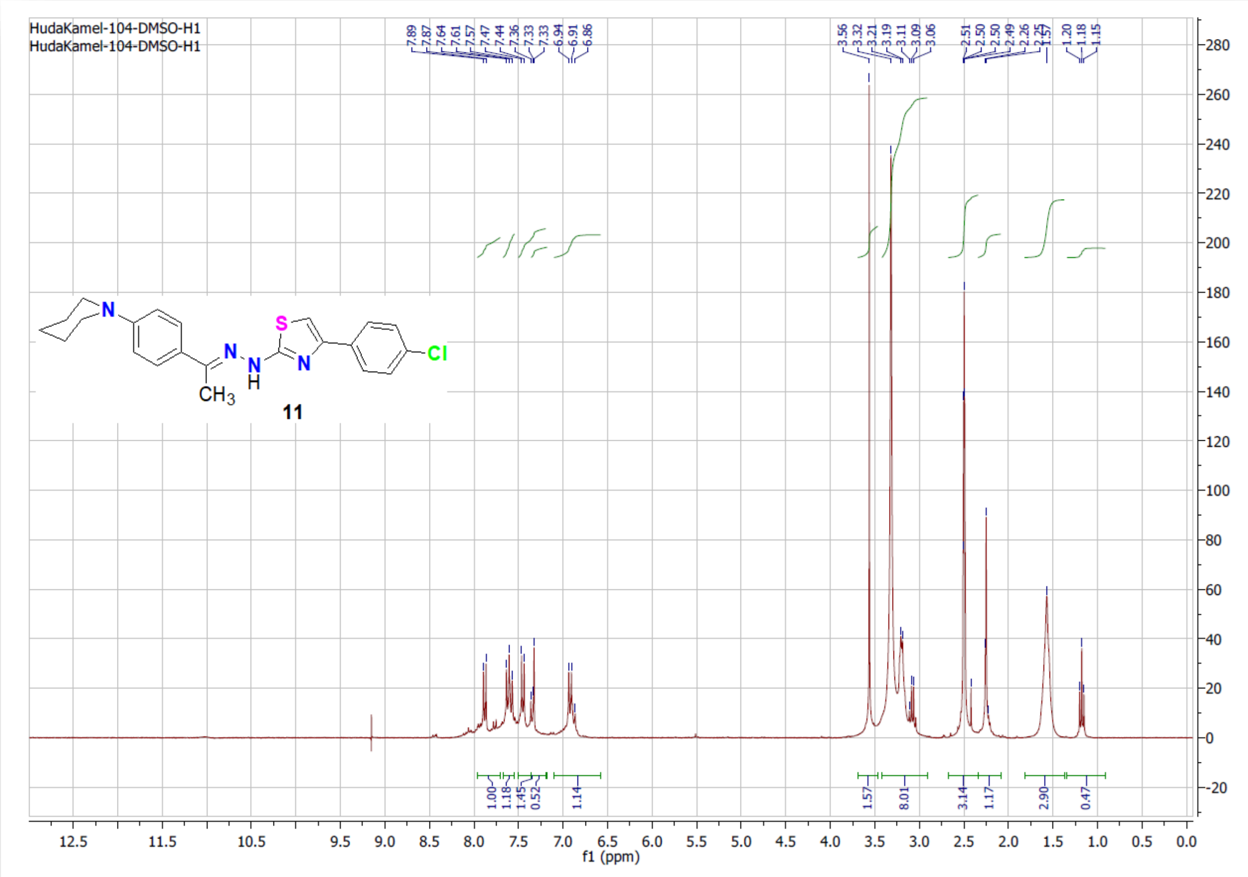

Figure S39. ^{1}H NMR spectrum of compound **11**

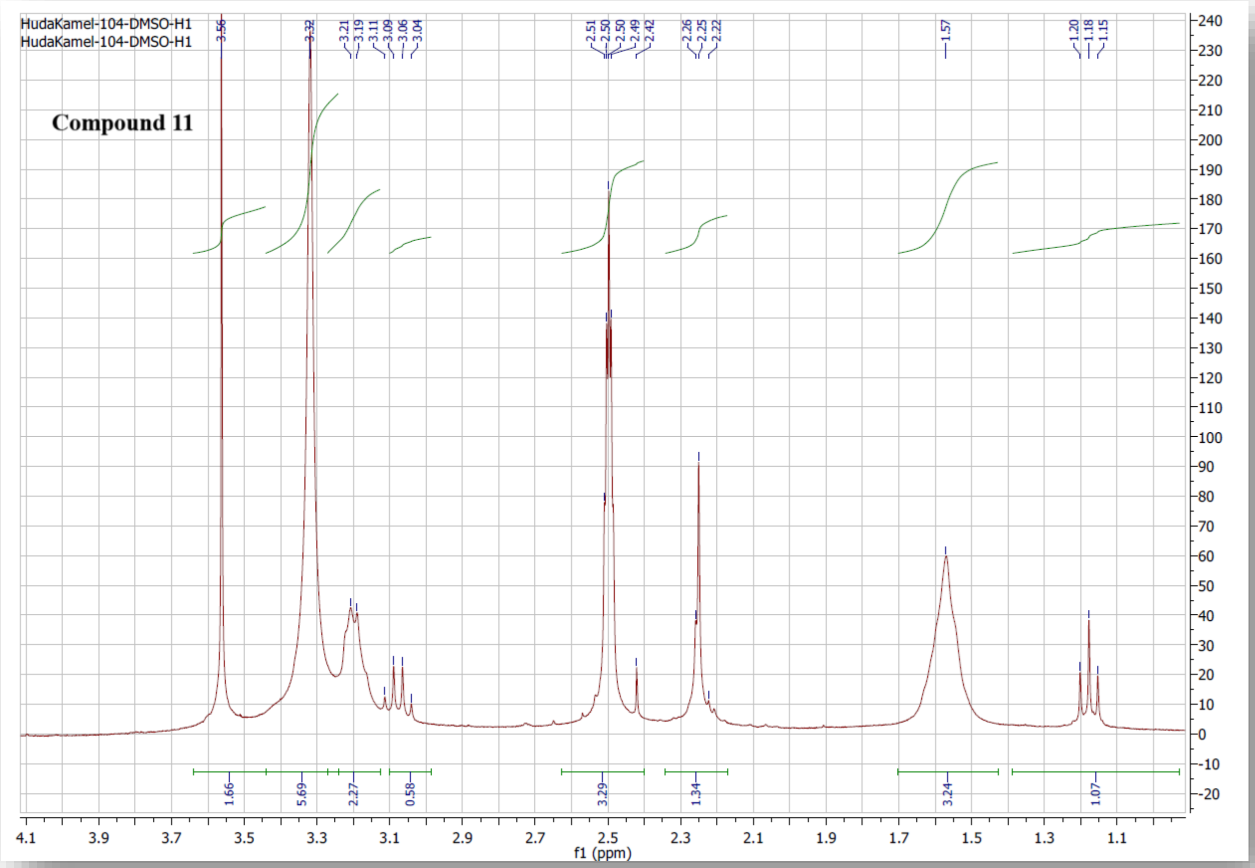

Figure S40. ^{1}H NMR spectrum of compound **11**

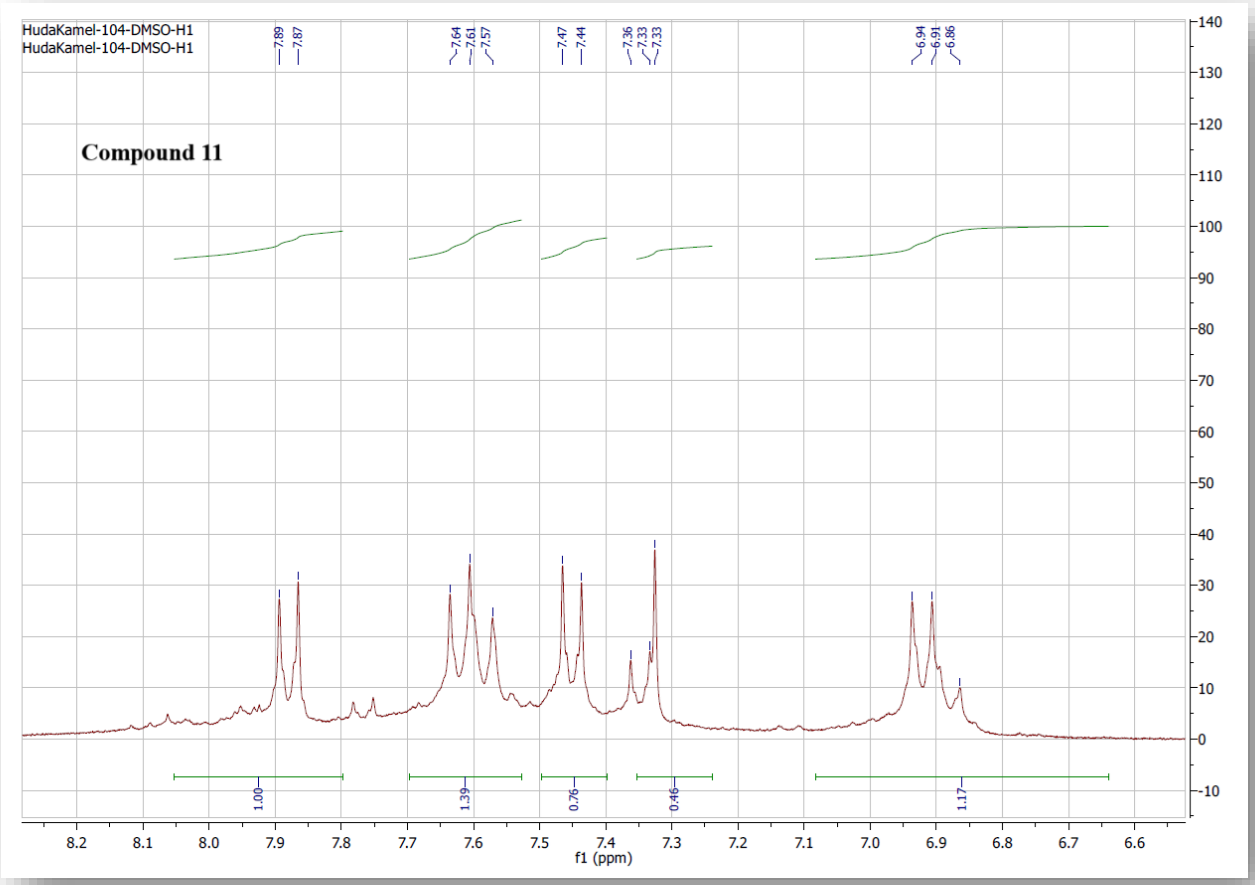

Figure S41. ^{1}H NMR spectrum of compound **11.**

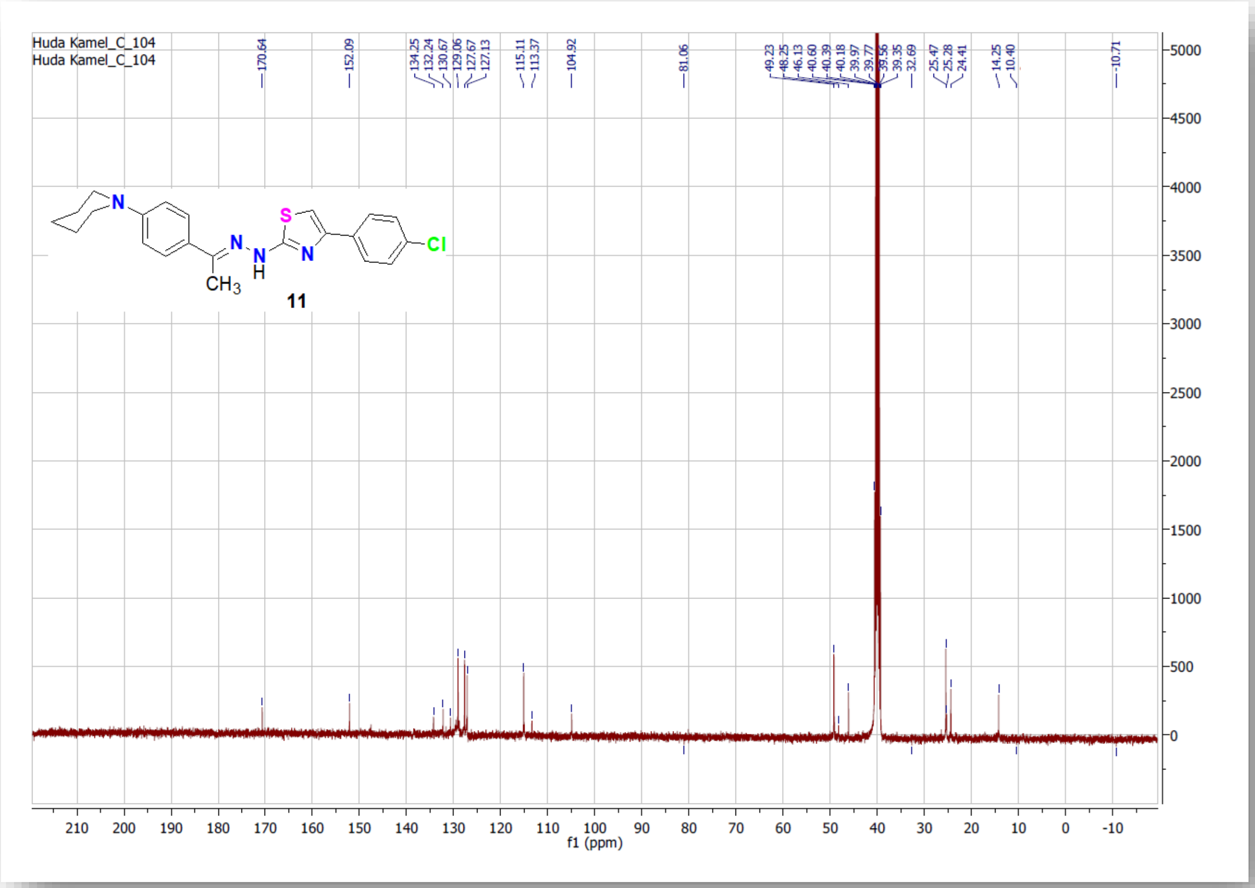

Figure S42. ^{13}C NMR spectrum of compound **11.**

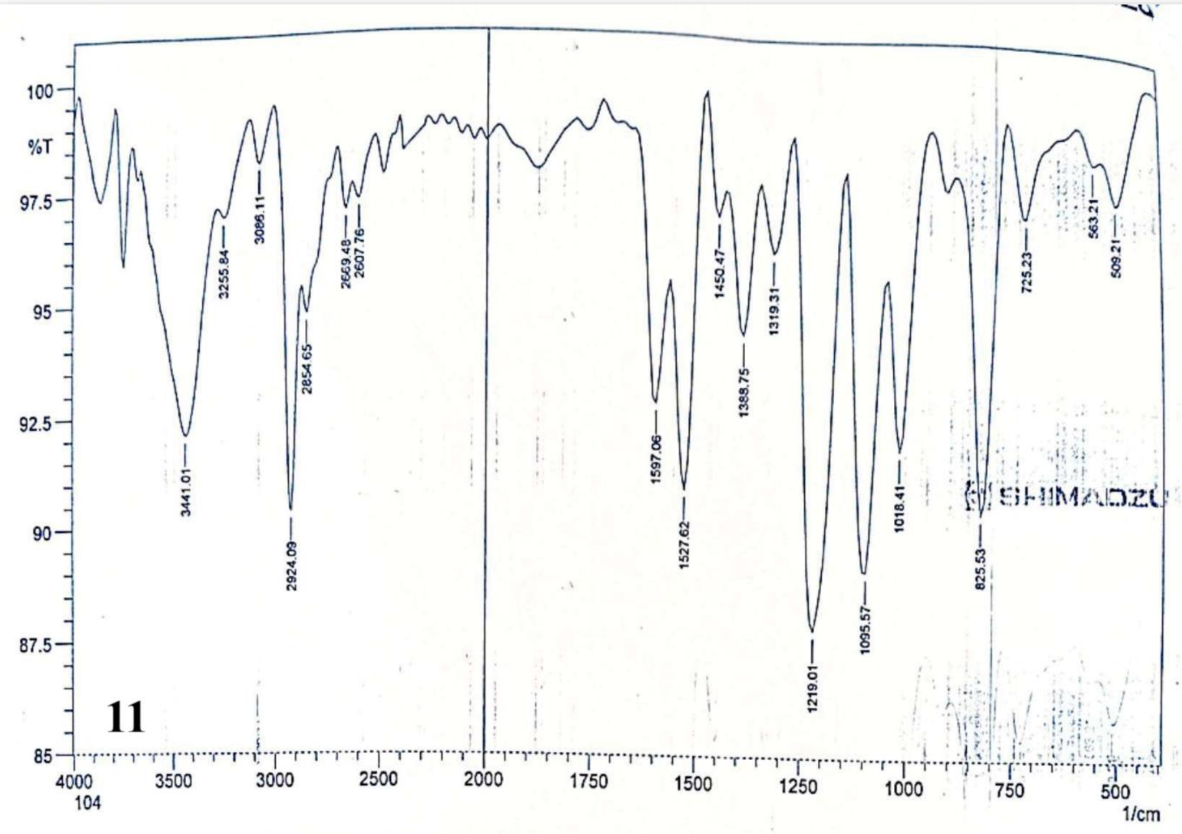

	Peak	Intensity	Corr. Intensity	Base (H)	Base (L)	Area	Corr. Area
1	509.21	97.549	1.43	540.07	416.62	0.645	0.341
2	563.21	98.43	0.366	601.79	540.07	0.338	0.039
3	725.23	97.106	2.149	771.53	601.79	1.125	0.576
4	825.53	90.489	8.217	894.97	771.53	2.646	1.904
5	1018.41	91.919	4.783	1049.28	948.98	2.063	0.884
6	1095.57	89.184	7.578	1149.57	1049.28	3.261	1.865
7	1219.01	87.863	10.478	1273.02	1157.29	4.139	3.261
8	1319.31	96.26	1.948	1350.17	1273.02	0.934	0.363
9	1388.75	94.427	3.275	1435.04	1350.17	1.441	0.581
10	1450.47	97.021	1.29	1481.33	1435.04	0.43	0.159
11	1527.62	90.977	6.489	1566.2	1481.33	2.072	1.189
12	1597.06	92.865	3.906	1651.07	1566.2	1.719	0.676
13	2607.76	97.325	0.631	2638.62	2530.61	0.981	0.117
14	2669.48	97.093	0.916	2708.06	2638.62	0.741	0.148
15	2854.65	94.791	1.008	2877.79	2708.06	2.588	0.268
16	2924.09	90.402	6.329	3016.67	2877.79	3.199	1.6
17	3086.11	98.151	1.101	3132.4	3016.67	0.639	0.276
18	3255.84	96.949	0.601	3286.7	3132.4	1.438	0.18
19	3441.01	92.109	5.418	3664.75	3286.7	8.93	4.957

Figure S43. IR spectrum of compound **11.**

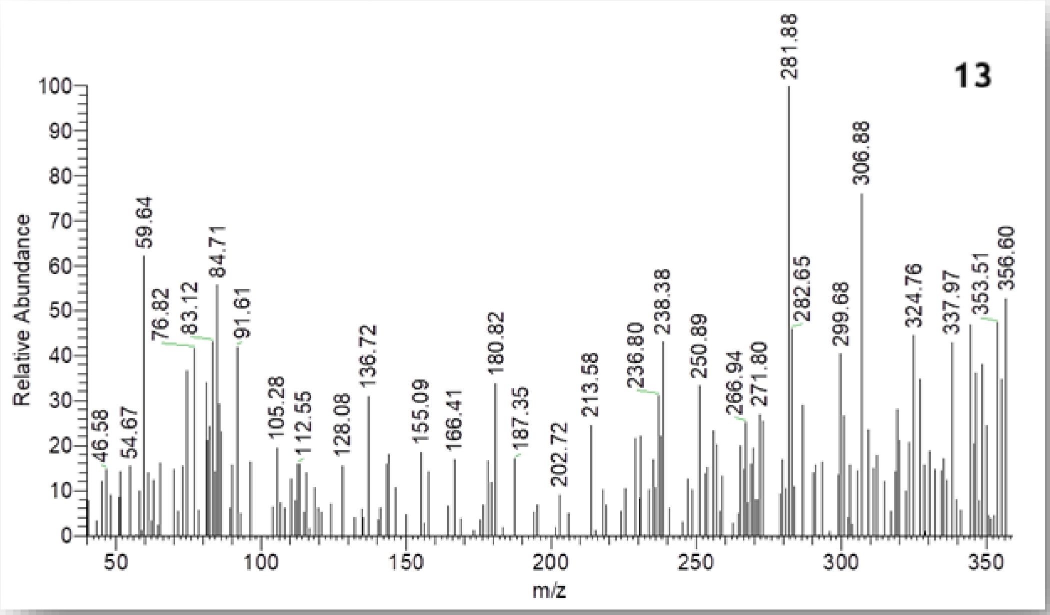

Figure S44. Mass spectrum of compound **13.**

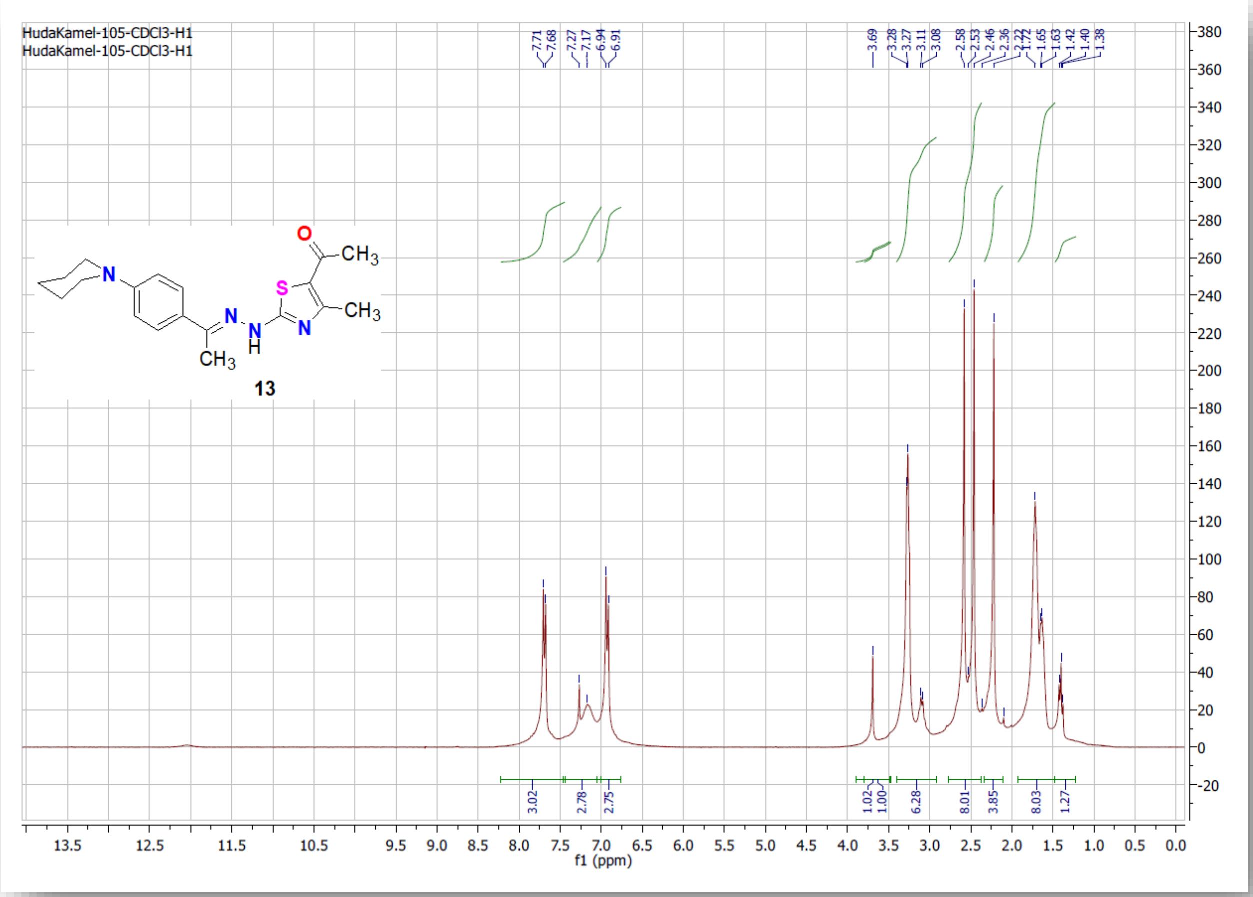

Figure S45. ^{1}H NMR spectrum of compound **13.**

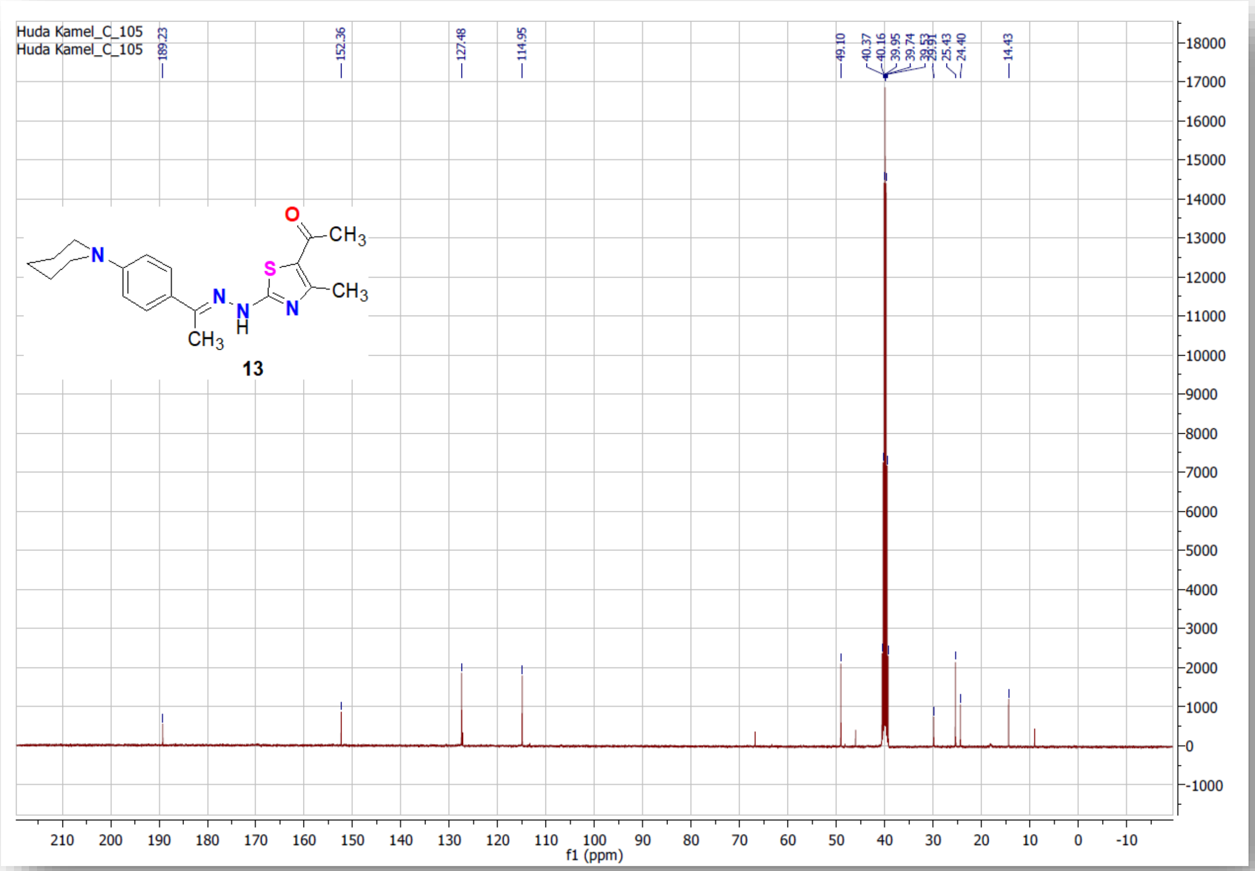

Figure S46. ^{13}C NMR spectrum of compound **13.**

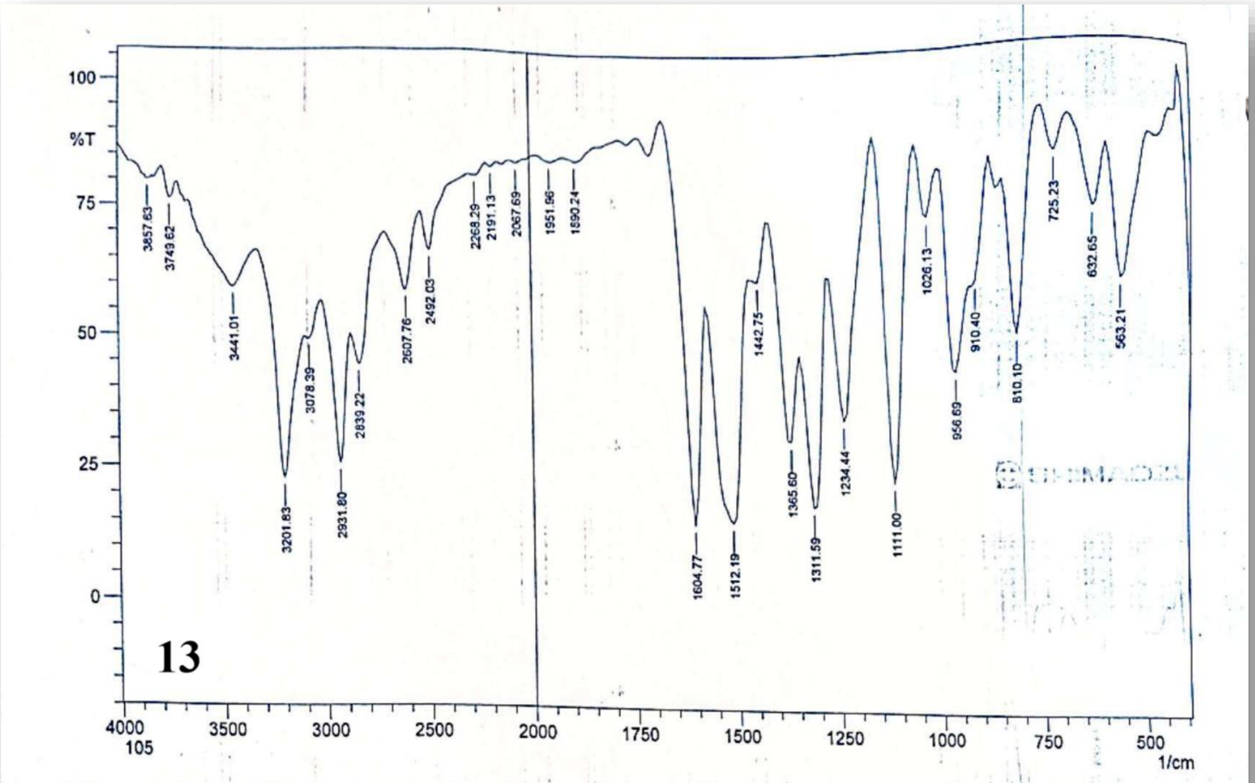

	Peak	Intensity	Corr. Intensity	Base (H)	Base (L)	Area	Corr. Area
1	563.21	62.854	25.034	601.79	501.49	12.399	6.912
2	632.65	75.917	11.183	686.66	609.51	6.391	2.162
3	725.23	86.127	7.34	763.81	694.37	3.119	1.108
4	810.1	53.45	33.932	848.68	763.81	11.98	7.397
5	910.4	62.552	5.085	918.12	887.26	4.42	0.49
6	956.69	46.635	25.455	995.27	925.83	16.971	6.775
7	1026.13	75.663	10.766	1056.99	1002.98	5.041	1.612
8	1111	24.851	62.416	1149.57	1064.71	25.189	20.138
9	1234.44	36.52	35.24	1265.3	1157.29	25.003	12.894
10	1311.59	19.665	32.725	1334.74	1273.02	29.694	13.752
11	1365.6	31.978	25.391	1411.89	1342.46	23.968	8.004
12	1442.75	62.98	4.918	1458.18	1419.61	6.658	0.673
13	1512.19	15.977	43.647	1566.2	1465.9	53.505	30.878
14	1604.77	15.523	53.95	1666.5	1573.91	33.332	20.031
15	1890.24	85.105	1.388	1913.39	1782.23	7.616	0.324
16	1951.96	84.766	1.134	1982.82	1921.1	4.279	0.202
17	2067.69	84.26	0.809	2098.55	1990.54	7.682	0.257
18	2191.13	83.275	0.785	2214.28	2160.27	4.173	0.112
19	2268.29	81.456	0.881	2291.43	2222	5.918	0.228
20	2492.03	66.721	8.644	2530.61	2299.15	27.186	2.491
21	2607.76	59.026	13.189	2700.34	2538.32	29.285	6.15
22	2839.22	44.717	8.843	2870.08	2708.06	39.213	3.917
23	2931.8	25.872	26.932	3008.95	2877.79	52.46	16.374
24	3078.39	49.382	1.673	3093.82	3016.67	21.596	0.624
25	3201.83	23.005	34.032	3325.28	3101.54	82.667	28.122
26	3441.01	59.111	10.175	3657.04	3332.99	61.559	12.549
27	3749.62	76.179	4.374	3788.19	3718.76	7.414	0.886
28	3857.63	79.865	1.23	3934.78	3834.49	9.053	0.294

Figure S47. IR spectrum of compound **13.**

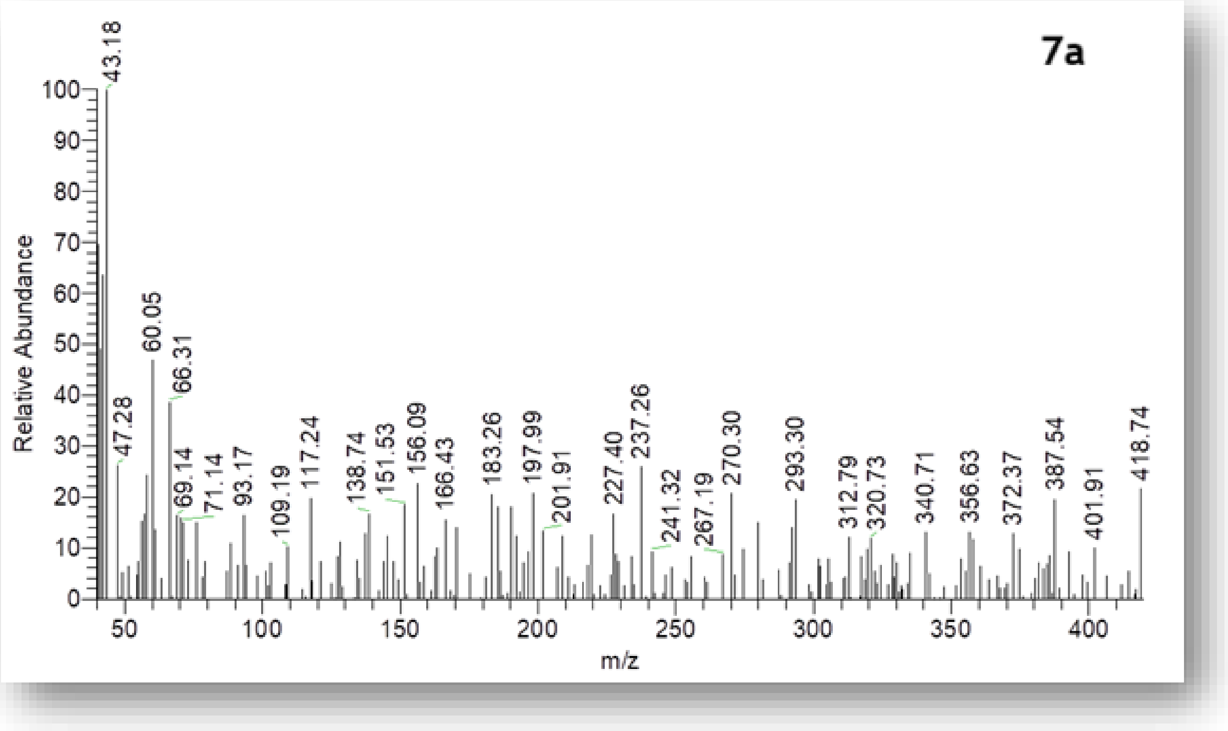

Figure S48. Mass spectrum of compound **7a.**

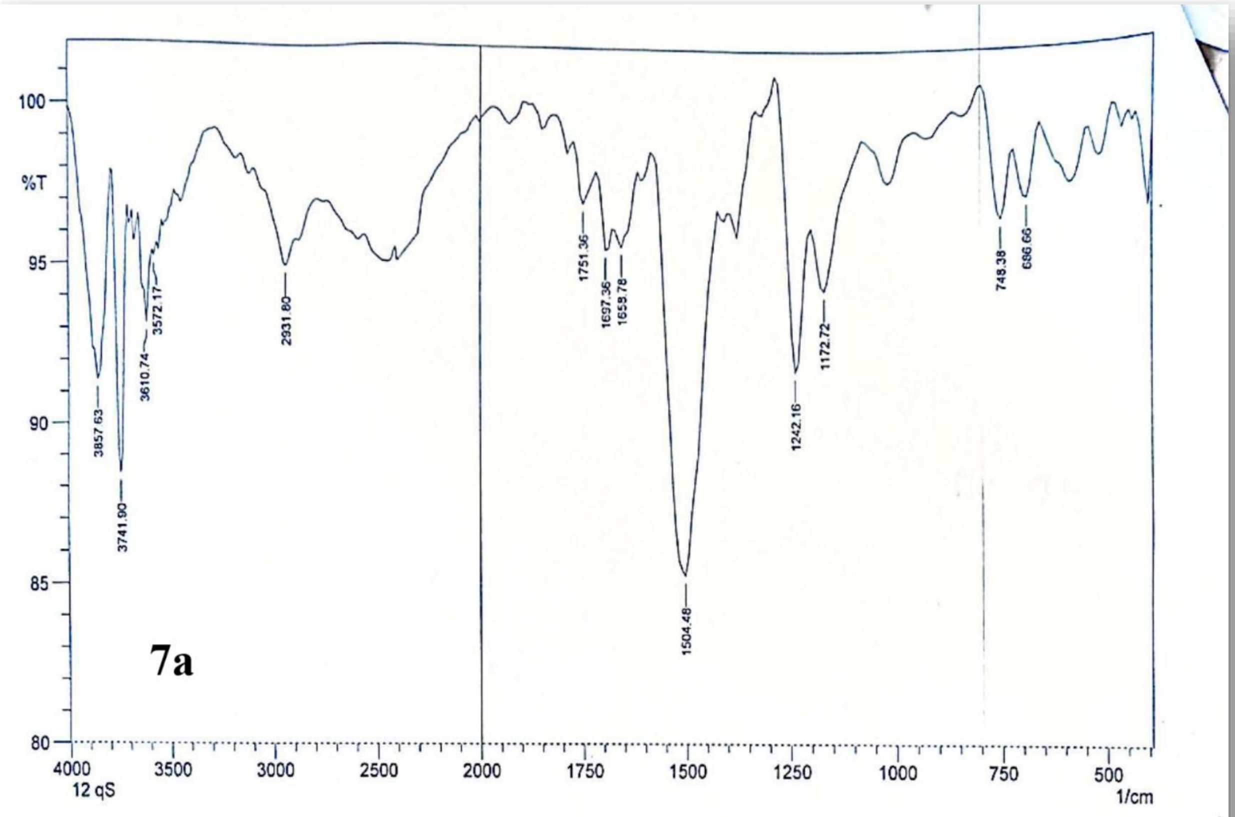

	Peak	Intensity	Corr. Intensity	Base (H)	Base (L)	Area	Corr. Area
1	686.66	97.242	1.914	717.52	655.8	0.511	0.283
2	748.38	96.569	2.985	794.67	717.52	0.585	0.501
3	1172.72	94.177	2.718	1203.58	1080.14	1.916	0.617
4	1242.16	91.596	6.817	1288.45	1203.58	1.664	1.147
5	1504.48	85.244	12.336	1581.63	1427.32	6.486	4.811
6	1658.78	95.5	1.263	1681.93	1620.21	1.022	0.202
7	1697.36	95.408	1.397	1720.5	1681.93	0.612	0.099
8	1751.36	96.887	1.572	1774.51	1720.5	0.575	0.194
9	2931.8	94.803	1.343	3093.82	2885.51	3.442	0.463
10	3572.17	95.139	0.241	3579.88	3556.74	0.484	0.013
11	3610.74	93.072	2.733	3649.32	3579.88	1.705	0.432
12	3741.9	88.405	8.804	3788.19	3703.33	2.668	1.644
13	3857.63	91.353	7.122	4004.22	3788.19	4.599	3.496

Figure S49. IR spectrum of compound **7a.**

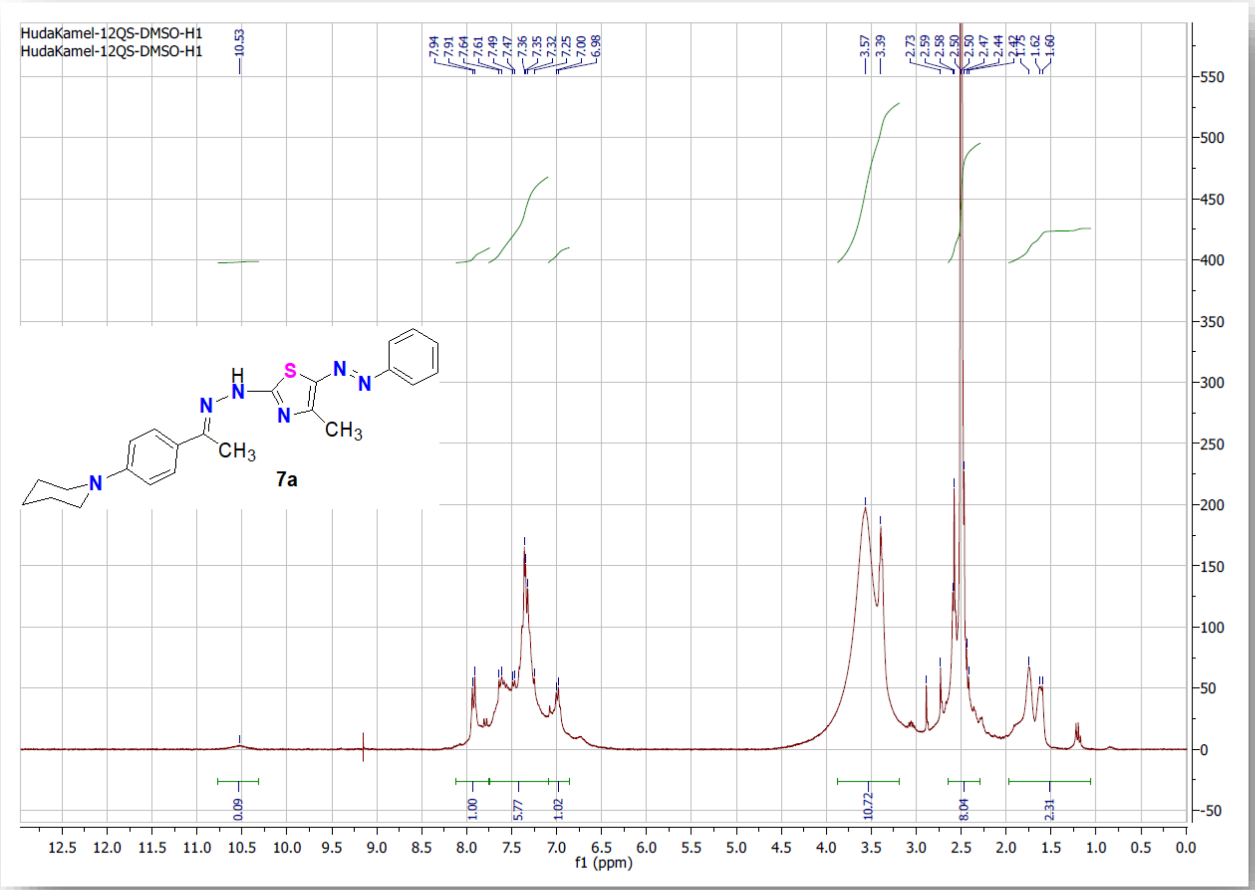

Figure S50. ^{1}H NMR spectrum of compound **7a.**

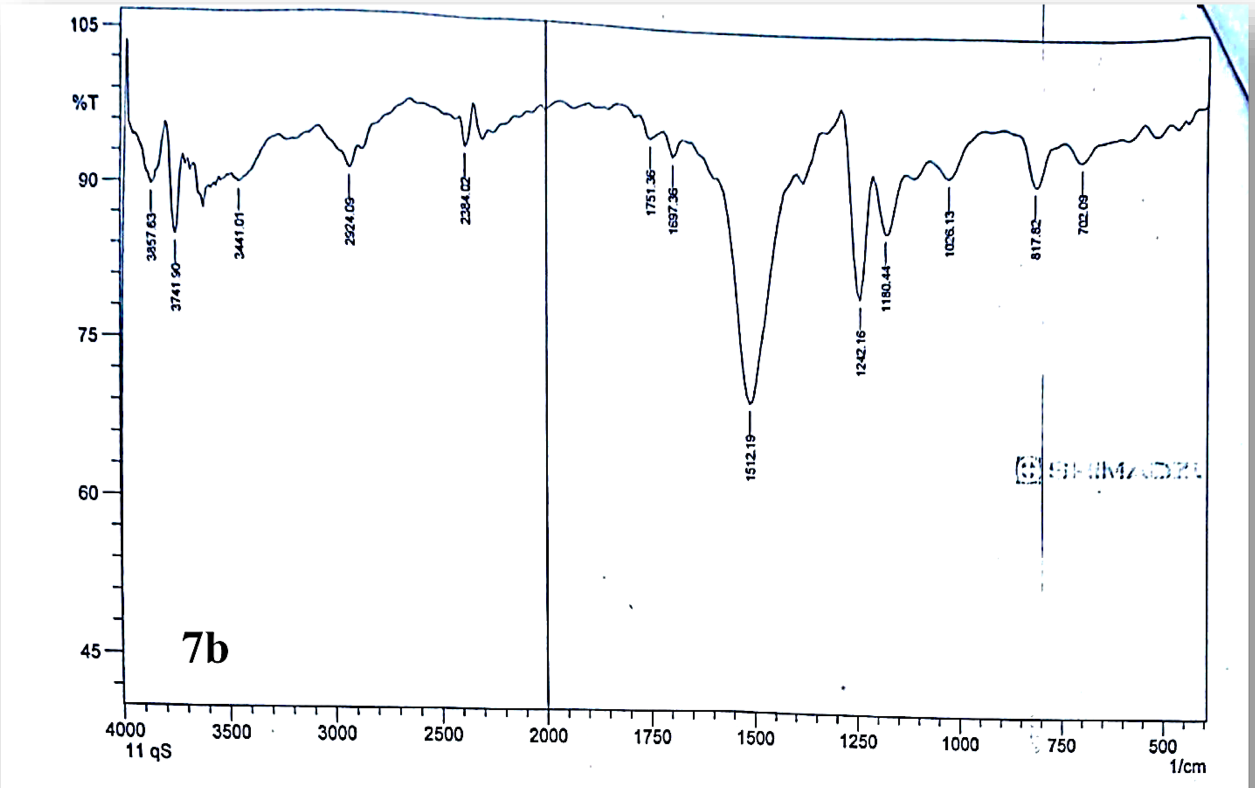

	Peak	Intensity	Corr. Intensity	Base (H)	Base (L)	Area	Corr. Area
1	702.09	94.512	2.121	748.38	601.79	2.6	0.495
2	817.82	92.132	4.901	894.97	748.38	2.929	1.032
3	1026.13	93.121	2.769	1072.42	941.26	2.794	0.639
4	1180.44	87.674	5.68	1211.3	1134.14	3.372	1.076
5	1242.16	81.159	14.664	1280.73	1211.3	3.926	2.626
6	1512.19	70.332	23.867	1666.5	1404.18	17.588	11.019
7	1697.36	94.119	2.161	1720.5	1674.21	0.965	0.202
8	1751.36	95.746	1.585	1782.23	1720.5	0.913	0.188
9	2384.02	93.955	3.273	2407.16	2345.44	1.248	0.541
10	2924.09	91.541	2.401	3062.96	2885.51	5.296	0.908
11	3441.01	89.913	1.228	3471.87	3255.84	7.796	0.601
12	3741.9	84.848	8.967	3788.19	3703.33	4.077	1.775
13	3857.63	89.659	5.279	3927.07	3788.19	5.041	1.928

Figure S51. IR spectrum of compound **7b.**

Lab Report

Table S1. In vitro assessment of target compounds against VEGFR2 enzyme.

Compound code	MW g/mol	VEGFR2 IC50 uM
**7b=11qs**	432.59	**0.134**±0.004
**7a=12qs**	418.56	**1.03**±0.032
**7c =13qs**	453.01	**0.073**±0.002
**7d = 17qs**	463.56	**0.13**±0.004
**7e=101qs**	432.59	**0.762**±0.024
**7f=102qs**	453.01	**1.868**±0.058
**7g=103qs**	463.56	**0.279**±0.009
**11=104qs**	410.96	**0.858**±0.027
**13-=105qs**	356.49	**0.181**±0.006
**9a=106qs**	466.60	**0.6**±0.019
**9b=107qs**	480.63	**0.049**±0.002
**9c=108qs**	501.05	**0.093**±0.003
**Sunitinib**	398.474	**0.118**±0.003

Detailed Results

VEGFR2

code	IC50	conc	log	%inh	T2	T1	ΔT	RFU2	RFU1	ΔRFU	slope	K.Activity
11qs		100	2	93	30	0	30	6402	0	6402	3052	8.3906
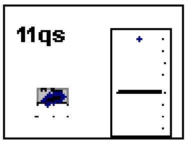		10	1	82.8	30	0	30	15749	0	15749	3052	20.641
		1	0	70	30	0	30	27505	0	27505	3052	36.048
		0.1	-1	49.9	30	0	30	45882	0	45882	3052	60.134
		0.01	-2	26.6	30	0	30	67189	0	67189	3052	88.059
EC				0	30	0	30	91557	0	91557	3052	120
code	IC50	conc	log	%inh	T2	T1	ΔT	RFU2	RFU1	ΔRFU	slope	K.Activity
12qs		100	2	89.2	30	0	30	9932	0	9932	3052	13.017
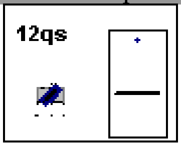		10	1	75.1	30	0	30	22826	0	22826	3052	29.916
		1	0	47.2	30	0	30	48361	0	48361	3052	63.383
		0.1	-1	26.9	30	0	30	66959	0	66959	3052	87.758
		0.01	-2	10.4	30	0	30	82039	0	82039	3052	107.52
EC				0	30	0	30	91557	0	91557	3052	120
code	IC50	conc	log	%inh	T2	T1	ΔT	RFU2	RFU1	ΔRFU	slope	K.Activity
13qs		100	2	96	30	0	30	3661	0	3661	3052	4.7982
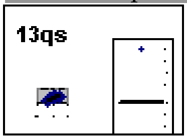		10	1	87.4	30	0	30	11575	0	11575	3052	15.17
		1	0	71.6	30	0	30	26038	0	26038	3052	34.126
		0.1	-1	56.6	30	0	30	39777	0	39777	3052	52.132
		0.01	-2	30.5	30	0	30	63594	0	63594	3052	83.347
EC				0	30	0	30	91557	0	91557	3052	120
code	IC50	conc	log	%inh	T2	T1	ΔT	RFU2	RFU1	ΔRFU	slope	K.Activity
17qs		100	2	95.2	30	0	30	4395	0	4395	3052	5.7602
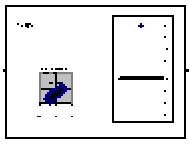		10	1	85.6	30	0	30	13227	0	13227	3052	17.336
		1	0	68.6	30	0	30	28742	0	28742	3052	37.67

		0.1	-1	50.5	30	0	30	45339	0	45339	3052	59.422
		0.01	-2	26.5	30	0	30	67283	0	67283	3052	88.182
EC				0	30	0	30	91557	0	91557	3052	120
	[redacted]		[redacted]		[redacted]		[redacted]		[redacted]			[redacted]
code	IC50	conc	log	%inh	T2	T1	ΔT	RFU2	RFU1	ΔRFU	slope	K.Activity
101qs		100	2	92.5	30	0	30	6852	0	6852	3052	8.9803
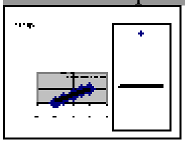		10	1	78.8	30	0	30	19437	0	19437	3052	25.474
		1	0	57.8	30	0	30	38622	0	38622	3052	50.619
		0.1	-1	28.7	30	0	30	65294	0	65294	3052	85.575
		0.01	-2	5.44	30	0	30	86579	0	86579	3052	113.47
EC				0	30	0	30	91557	0	91557	3052	120
	[redacted]		[redacted]		[redacted]		[redacted]		[redacted]			[redacted]
code	IC50	conc	log	%inh	T2	T1	ΔT	RFU2	RFU1	ΔRFU	slope	K.Activity
102qs		100	2	87.6	30	0	30	11386	0	11386	3052	14.923
		10	1	70.3	30	0	30	27227	0	27227	3052	35.684
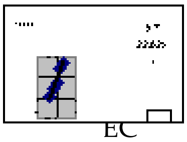		1	0	39.5	30	0	30	55394	0	55394	3052	72.6
		0.1	-1	20.4	30	0	30	72909	0	72909	3052	95.556
		0.01	-2	2.41	30	0	30	89351	0	89351	3052	117.1
EC				0	30	0	30	91557	0	91557	3052	120
	[redacted]				[redacted]		[redacted]		[redacted]			
code	IC50	conc	log	%inh	T2	T1	ΔT	RFU2	RFU1	ΔRFU	slope	K.Activity
103qs		100	2	95.9	30	0	30	3742	0	3742	3052	4.9043
		10	1	83.5	30	0	30	15066	0	15066	3052	19.746
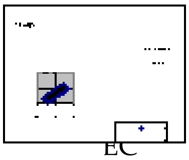		1	0	67.6	30	0	30	29648	0	29648	3052	38.857
		0.1	-1	36.3	30	0	30	58352	0	58352	3052	76.477
		0.01	-2	21.2	30	0	30	72118	0	72118	3052	94.519
EC				0	30	0	30	91557	0	91557	3052	120
	[redacted]		[redacted]		[redacted]		[redacted]		[redacted]			[redacted]
code	IC50	conc	log	%inh	T2	T1	ΔT	RFU2	RFU1	ΔRFU	slope	K.Activity
104qs		100	2	94	30	0	30	5537	0	5537	3052	7.2569
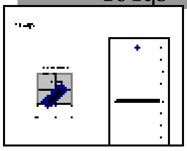		10	1	78.8	30	0	30	19428	0	19428	3052	25.463
		1	0	51.5	30	0	30	44376	0	44376	3052	58.16
		0.1	-1	25.4	30	0	30	68307	0	68307	3052	89.524
		0.01	-2	7.86	30	0	30	84364	0	84364	3052	110.57
EC				0	30	0	30	91557	0	91557	3052	120
	[redacted]		[redacted]		[redacted]		[redacted]		[redacted]			[redacted]
code	IC50	conc	log	%inh	T2	T1	ΔT	RFU2	RFU1	ΔRFU	slope	K.Activity
105qs		100	2	95.8	30	0	30	3882	0	3882	3052	5.0878
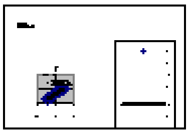		10	1	86.4	30	0	30	12453	0	12453	3052	16.321
		1	0	70.5	30	0	30	27037	0	27037	3052	35.435
		0.1	-1	41.1	30	0	30	53934	0	53934	3052	70.687
		0.01	-2	25.4	30	0	30	68331	0	68331	3052	89.556
EC				0	30	0	30	91557	0	91557	3052	120
	[redacted]		[redacted]		[redacted]		[redacted]		[redacted]			[redacted]
code	IC50	conc	log	%inh	T2	T1	ΔT	RFU2	RFU1	ΔRFU	slope	K.Activity
106qs		100	2	92.6	30	0	30	6744	0	6744	3052	8.8388
		10	1	83.8	30	0	30	14869	0	14869	3052	19.488
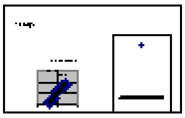		1	0	61.7	30	0	30	35025	0	35025	3052	45.904
		0.1	-1	30.9	30	0	30	63287	0	63287	3052	82.945
		0.01	-2	6.04	30	0	30	86033	0	86033	3052	112.76
EC				0	30	0	30	91557	0	91557	3052	120
	[redacted]		[redacted]		[redacted]		[redacted]		[redacted]			[redacted]
code	IC50	conc	log	%inh	T2	T1	ΔT	RFU2	RFU1	ΔRFU	slope	K.Activity

107qs		100	2	96.7	30	0	30	2996	0	2996	3052	3.9266
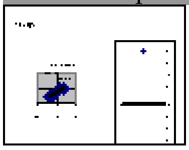		10	1	89.2	30	0	30	9872	0	9872	3052	12.938
		1	0	75.1	30	0	30	22818	0	22818	3052	29.906
		0.1	-1	53.2	30	0	30	42837	0	42837	3052	56.143
		0.01	-2	37.3	30	0	30	57382	0	57382	3052	75.206
EC				0	30	0	30	91557	0	91557	3052	120
	[redacted]		[redacted]		[redacted]				[redacted]	[redacted]		[redacted]

code	IC50	conc	log	%inh	T2	T1	ΔT	RFU2	RFU1	ΔRFU	slope	K.Activity
108qs		100	2	95.3	30	0	30	4285	0	4285	3052	5.616
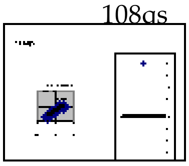		10	1	87.2	30	0	30	11763	0	11763	3052	15.417
		1	0	71.3	30	0	30	26237	0	26237	3052	34.387
		0.1	-1	54.3	30	0	30	41886	0	41886	3052	54.896
		0.01	-2	28.2	30	0	30	65697	0	65697	3052	86.104
EC				0	30	0	30	91557	0	91557	3052	120
	[redacted]		[redacted]		[redacted]		[redacted]		[redacted]	[redacted]		[redacted]

code	IC50	conc	log	%inh	T2	T1	ΔT	RFU2	RFU1	ΔRFU	slope	K.Activity
Sunitinib		100	2	96	30	0	30	3647	0	3647	3052	4.7798
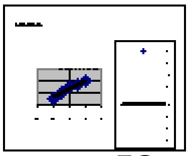		10	1	87.4	30	0	30	11559	0	11559	3052	15.149
		1	0	72.8	30	0	30	24882	0	24882	3052	32.611
		0.1	-1	49.5	30	0	30	46239	0	46239	3052	60.602
		0.01	-2	26.4	30	0	30	67384	0	67384	3052	88.315
EC				0	30	0	30	91557	0	91557	3052	120
	[redacted]		[redacted]		[redacted]		[redacted]		[redacted]	[redacted]		[redacted]

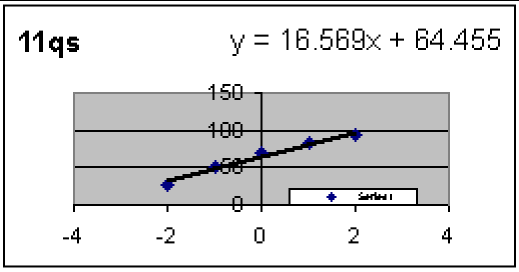

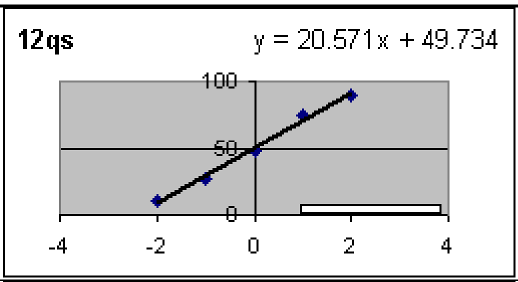

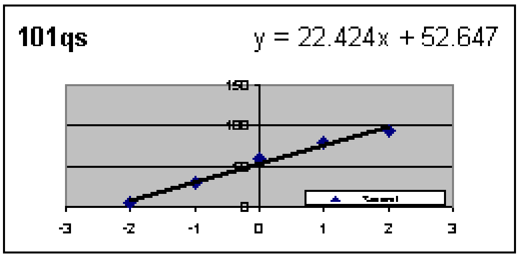

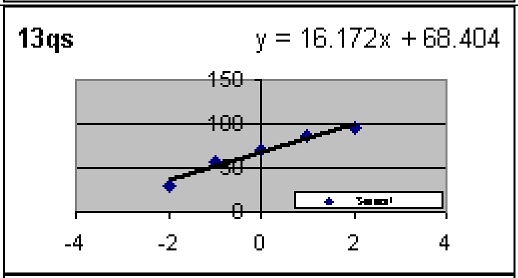

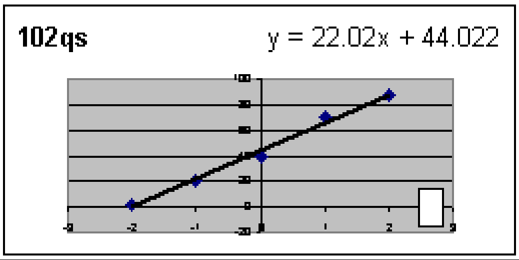

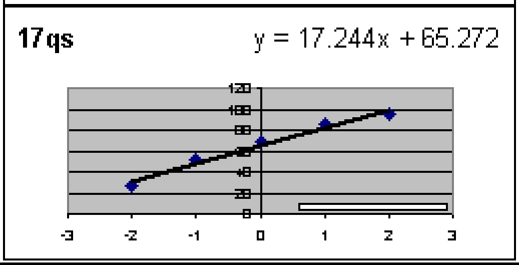

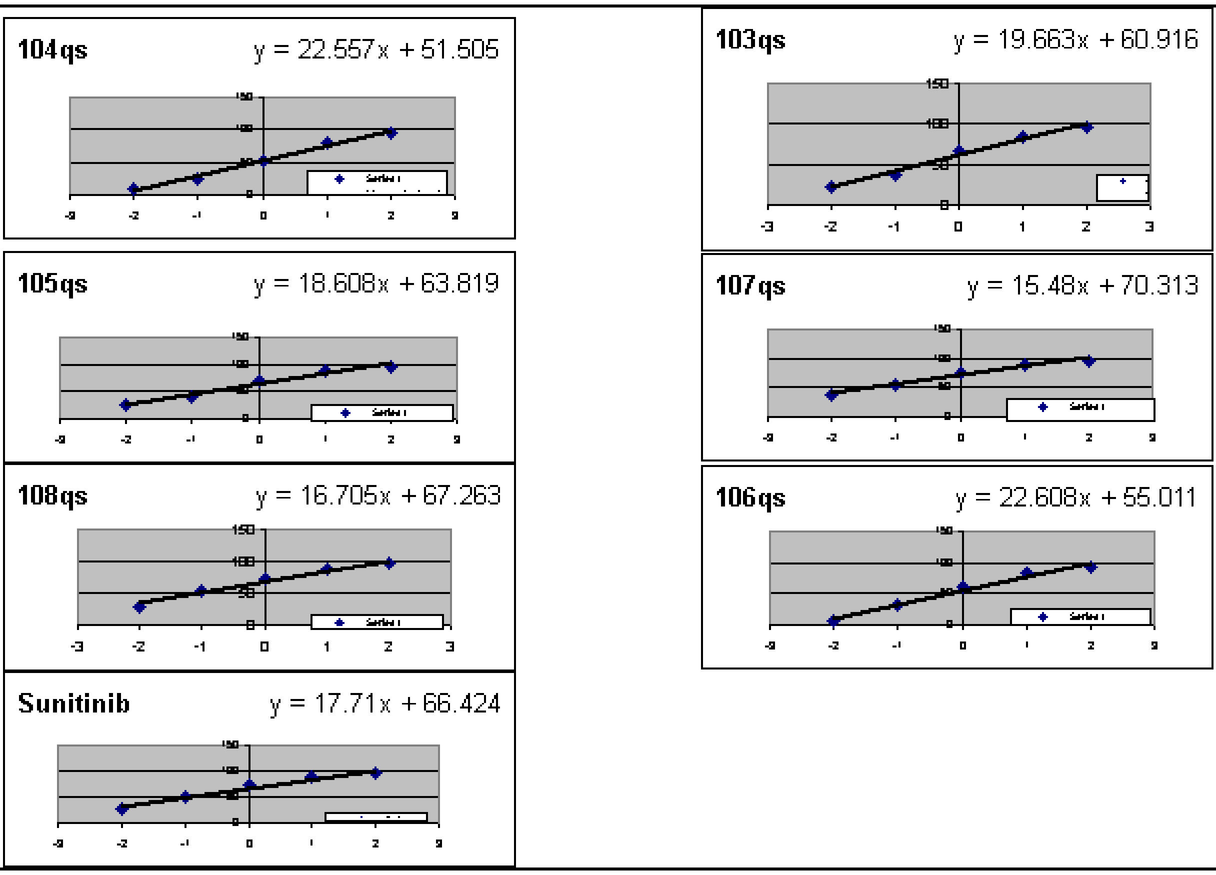

*Cytotoxicity results

Table S2. In vitro assessment of cytotoxicity.

Sample		Cytotoxicity IC50 uM	
**code**	**MW g/mol**	**A498**	WI38
**13qs**	**453.01**	**7.866±0.27**	**65.28±**2.31
**107qs**	**480.63**	**22.67±0.77**	---
**108qs**	**501.05**	**17.81±0.6**	---
**Sunitinib**	**398.474**	**2.955±0.1**	**24.93±**0.87

Detailed results

**Researcher**	**Assay**	**Date**		**Cells**
Dr.Hanan Gaber	MTT	16/02/2025		A498

**Blank**	**CC**	**Sample No.**			**13qs/A498**		**Sample No.**			**107qs/A498**	
B	C	100uM	25uM	6.3uM	1.6uM	0.4uM	100uM	25uM	6.3uM	1.6uM	0.4uM
B	C	100uM	25uM	6.3uM	1.6uM	0.4uM	100uM	25uM	6.3uM	1.6uM	0.4uM
B	C	100uM	25uM	6.3uM	1.6uM	0.4uM	100uM	25uM	6.3uM	1.6uM	0.4uM

ROBONIK P2000 Eia reader

WL 450 nm
Reference: 630 nm

	0.001	0.574	0.188	0.233	0.292	0.354	0.418	0.225	0.279	0.342	0.396	0.452
	0.001	0.561	0.176	0.241	0.303	0.363	0.425	0.237	0.281	0.339	0.379	0.441
	0.001	0.578	0.167	0.229	0.287	0.342	0.415	0.222	0.277	0.331	0.404	0.437
mean	4E-04	0.571	0.177	0.234	0.294	0.353	0.419	0.228	0.279	0.33733	0.393	0.44333
%		`	30.998	41.04	51.49	61.82	73.44	39.9299	48.8616	59.0776	68.82662	77.6416

13qs/A498

log conc.	% viability
2	31
1.398	41.04
0.796	51.49
0.193	61.82
-0.41	73.44

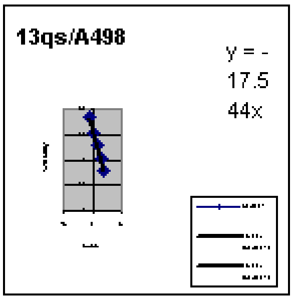

IC50=

107qs/A498

log conc.	% viability
2	39.9299
1.39794	48.8616
0.79588	59.0776
0.19312	68.8266
-0.40894	77.6416

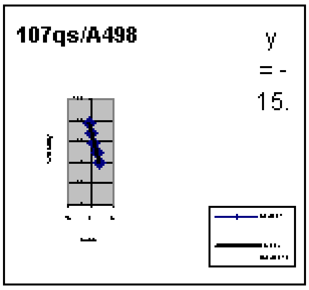

IC50=

	Blank	CC	Sample No.		108qs/A498			Sample No.		Sunitinib/A498	
	B	C	100uM	25uM	6.3uM	1.6uM	0.4uM	100uM	25uM	6.3uM	1.6uM	0.4uM
	B	C	100uM	25uM	6.3uM	1.6uM	0.4uM	100uM	25uM	6.3uM	1.6uM	0.4uM
	B	C	100uM	25uM	6.3uM	1.6uM	0.4uM	100uM	25uM	6.3uM	1.6uM	0.4uM

ROBONIK P2000 Eia reader
WL 450 nm
Reference: 630 nm

	0.001	0.593	0.229	0.275	0.351	0.435	0.507	0.188	0.225	0.286	0.321	0.376
	0.001	0.611	0.231	0.269	0.359	0.428	0.493	0.175	0.229	0.272	0.304	0.384
	0.001	0.602	0.207	0.264	0.338	0.444	0.512	0.184	0.231	0.265	0.325	0.366
mean	0.001	0.602	0.2223	0.269	0.349	0.436	0.504	0.18233	0.22833	0.27433	0.316667	0.37533
% viability			36.932	44.74	58.03	72.37	83.72	30.2879	37.9291	45.5703	52.60244	62.3477

108qs/A498

log conc.	% viability
2	36.93
1.398	44.74
0.796	58.03
0.193	72.37
-0.41	83.72

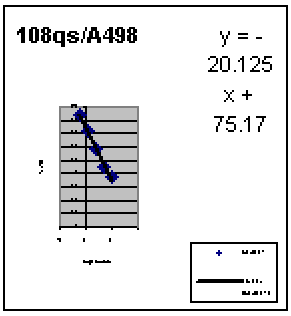

IC50=

Sunitinib/A498

log conc.	% viability
2	30.2879
1.39794	37.9291
0.79588	45.5703
0.19312	52.6024
-0.40894	62.3477

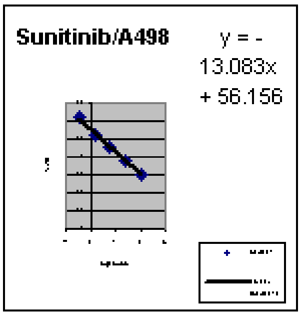

IC50=

	Blank	CC	Sample No. 13qs/WI38					Sample No. Sunitinib/WI38				
	B	C	100uM	25uM	6.3uM	1.6uM	0.4uM	100uM	25uM	6.3uM	1.6uM	0.4uM
	B	C	100uM	25uM	6.3uM	1.6uM	0.4uM	100uM	25uM	6.3uM	1.6uM	0.4uM
	B	C	100uM	25uM	6.3uM	1.6uM	0.4uM	100uM	25uM	6.3uM	1.6uM	0.4uM

ROBONIK P2000 Eia reader

WL 450 nm

Reference: 630 nm

	0.001	0.482	0.225	0.267	0.346	0.408	0.466	0.181	0.236	0.287	0.331	0.392
	0.003	0.466	0.219	0.281	0.337	0.419	0.472	0.177	0.236	0.284	0.339	0.404
	0.001	0.475	0.227	0.265	0.342	0.422	0.459	0.194	0.237	0.302	0.361	0.397
mean	0.002	0.474	0.2237	0.271	0.342	0.416	0.466	0.184	0.23633	0.291	0.343667	0.39767
% via-bility			47.154	57.13	72.03	87.77	98.17	38.7913	49.8243	61.3493	72.45257	83.837

13qs/WI38

2	47.15
1.398	57.13
0.796	72.03
0.193	87.77
-0.41	98.17

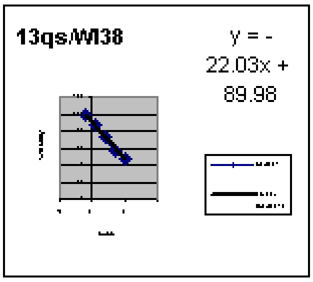

IC50=

Sunitinib/WI38

2	38.7913
1.39794	49.8243
0.79588	61.3493
0.19312	72.4526
-0.40894	83.837

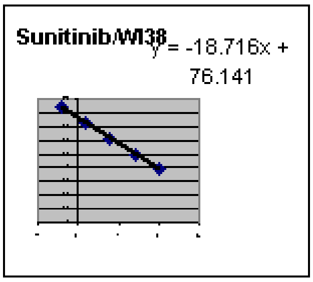

IC50=

Table S3. Cell cycle analysis of untreated and **7c**-treated A498 cells.

ser	Sample		DNA content			
	code	IC50 uM	%G0-G1	%S	%G2/M	Comment
1	**13qs /A498**	---	**88.15**	**8.88**	**2.97**	Cell cycle arrest@G1
2	**cont.A498**	---	**59.82**	**29.64**	**10.54**	---

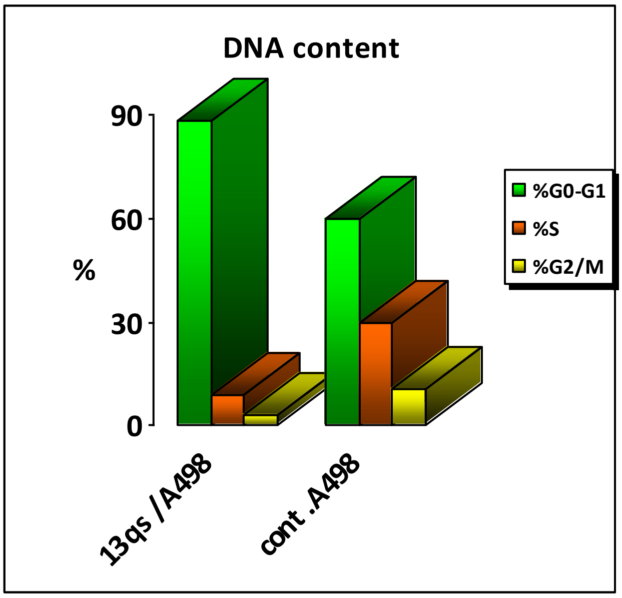

Figure S52. Cell cycle analyses of untreated and **7c**-treated A498 cells.

Table S4 Flow cytometric analysis of apoptosis and necrosis in untreated and **7c** -treated A498 cells

s	code	conc	Apoptosis			Necrosis
			Total	Early	Late	
1	13qs /A498	---	35.04	6.1	21.42	7.52
2	cont.A498	---	2.74	0.61	0.22	1.91

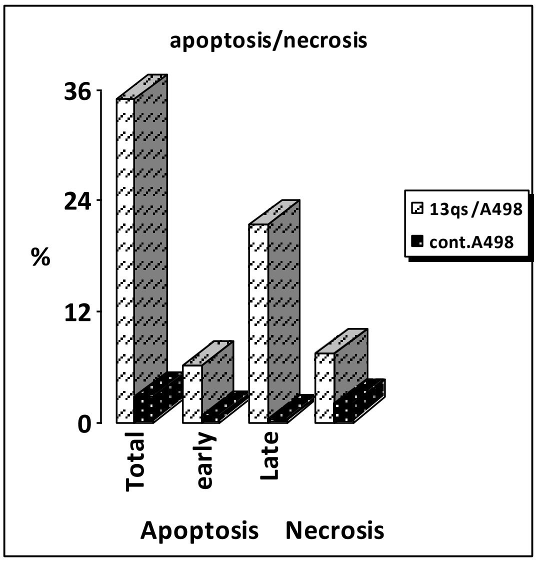

Figure S53. Flow cytometric analysis of apoptosis and necrosis in untreated and **7c** -treated A498 cells.
